# Ophthalmology in North America: Early Stories (1491-1801)

**DOI:** 10.1177/1179172117721902

**Published:** 2017-07-26

**Authors:** Christopher T Leffler, Stephen G Schwartz, Ricardo D Wainsztein, Adam Pflugrath, Eric Peterson

**Affiliations:** 1 Department of Ophthalmology, Virginia Commonwealth University, Richmond, VA, USA; 2 Department of Ophthalmology, Bascom Palmer Eye Institute, University of Miami Miller School of Medicine, Naples, FL, USA; 3 Instituto de la Visión, Buenos Aires, Argentina

**Keywords:** Ophthalmology history, cataract surgery

## Abstract

New World plants, such as tobacco, tomato, and chili, were held to have beneficial effects on the eyes. Indigenous healers rubbed or scraped the eyes or eyelids to treat inflammation, corneal opacities, and even eye irritation from smoke. European settlers used harsh treatments, such as bleeding and blistering, when the eyes were inflamed or had loss of vision with a normal appearance (gutta serena). In New Spain, surgery for corneal opacity was performed in 1601 and cataract couching in 1611. North American physicians knew of contralateral loss of vision after trauma or surgery (sympathetic ophthalmia), which they called “sympathy.” To date, the earliest identified cataract couching by a surgeon trained in the New World was performed in 1769 by John Bartlett of Rhode Island. The American Revolution negatively affected ophthalmology, as loyalist surgeons were expelled and others were consumed with wartime activities. After the war, cataract extraction was imported to America in earnest and academic development resumed. Charles F Bartlett, the son of John, performed cataract extraction but was also a “rapacious privateer.” In 1801, a doctor in the frontier territory of Kentucky observed anticholinergic poisoning by *Datura stramonium* (Jimsonweed) and suggested that this agent be applied topically to dilate the pupil before cataract extraction. John Warren at Harvard preferred couching in the 1790s, but, after his son returned from European training, recommended treating angle closure glaucoma by lens extraction. Other eye procedures described or advertised in America before the 19th century included enucleation, resection of conjunctival lesions or periocular tumors, treatment of lacrimal fistula, and fitting of prosthetic eyes.

## Introduction

The establishment of European colonies in the New World after 1492 brought about the proximity of indigenous, European, and African cultures, each with distinct approaches to medical care. Diseases such as smallpox, measles, scarlet fever, typhoid, cholera, diphtheria, and malaria were introduced to the Americas,^1^ where the native populations who lacked immunity suffered very high mortality rates. The Old World skills and techniques in every medical area, including ophthalmology, were gradually transferred to the Americas. In Europe, spectacles were used to correct refractive error and cataract couching was performed. European oculists and other surgeons traveled to the colonies, and early American settlers returned to Europe for training.

We present a review of some aspects of early North American ophthalmology not highlighted previously in the ophthalmology literature.^2–4^ This review discusses the following: (1) Native American cultures, (2) particular eye ailments and treatments, and (3) surgeon biographies and early American universities. We define North America to include the areas of present-day Canada, the United States, Mexico, the Caribbean, and Greenland.

A complete list of native American and European ophthalmic remedies during this period is beyond the scope of this work.^5^ The cases presented are merely illustrative examples of early American treatments.

We searched for terms such as eye, oculist, cataract, glaucoma, and retina in numerous databases: American newspapers,^6^ the Human Relations Area Files,^7^ the Colonial North American Project,^8^ American National Biography Online,^9^ the Dictionary of Canadian Biography,^10^ Hemeroteca Nacional Digital de México,^11^ Caribbean Newspapers (1718-1876),^12^ Early American Imprints (1639-1819),^13,14^ American Periodicals Series Online,^15^ Founders Online,^16^ Early English Books Online,^17^ and Gale Eighteenth Century Collections.^18^

## Native American Ophthalmology

### Overview

Some information about Native American understanding of the eye comes from the reports of European settlers. Of course, these observers wrote from their own perspectives and may not have always understood what they were seeing. The Native Americans may have been influenced by earlier European settlers. Moreover, some eye diseases treated by the Natives, such as smallpox and trachoma, were not even thought to have existed in pre-Columbian America. These limitations notwithstanding, the reports provide some idea of the types of treatments typical among indigenous peoples. In multiple areas, corneal opacities and eyelid ailments were treated by scraping the eyes or eyelids. In total, 9 of the 12 (75%) Native American healers we identified were women. We did not find evidence that Native cultures had identified the crystalline lens.

Native use of therapeutic phlebotomy (bloodletting) was not universal. The Nahuatl, Comanches, Maricopas, Montagnais, and tribes of Lower California performed therapeutic bleeding, but the Algonquins and certain Canadian tribes did not.^1^ In a few cases, the Native groups seemed to have learned of it from European settlers.^1^ When the Native Americans west of the Mississippi, did perform bleeding, “They seldom let blood in any considerable quantity, and never . . . until fainting is induced.”^19^

The mouth was one of the earliest medical tools, whether used to suck, lick, or blow. Some substances placed topically to treat eye disorders seem to be soothing, such as breast milk or coconut milk. Even today, the antimicrobial and anti-inflammatory properties of milk or saliva have been the subject of scientific investigation. In other cases of indigenous medicine, chemically or physically abrasive substances, such as peppers or pieces of shells, were applied to the eyes, often with some mechanical scraping to dislodge opacities or cause bleeding. These remedies, used for both inflammation and corneal opacities, were intermediate between medicine and surgery. Often, the soothing substances were applied to inflamed eyes, whereas the harsh treatments were used to remove opacities. However, this pattern did not apply universally.

### Mesoamerica and the Caribbean

The Nahuatl of Mesoamerica (which included the Aztecs) had anatomic terms for the eyelid (*ixquatolli*), eyebrow (*ixquamolli*), eye (*ixtelolotli*), and pupil (*tixtotouh*).^20^ Intraocular structures such as the crystalline lens were not described.^20^ Among the Aztecs of Mexico, wounds could be sutured and dressed.^5^

Francisco Hernández de Toledo was a physician who explored New Spain (in the region of present-day Mexico) from 1570 to 1577 and recorded the botanical life and its native uses. A variety of plants from New Spain and the Caribbean were applied to the eyes to treat inflammation. The tomato, a plant indigenous to the Americas, was considered by native healers to help with “blocked tear ducts and headaches.”^21^

Phlebotomy (bloodletting) was used for both religious and medical purposes among the Nahuatl.^5,22^ Therapeutic cupping was also performed.^5^ Cupping involves heating a cup placed on the skin. When the cup cools, a vacuum is created to draw blood to the surface. Cupping is performed throughout the world to this day.^23^ The Nahuatl often practiced wet cupping, which involves the same procedure along with skin incisions, to allow blood to collect in the cup.^5^

Women healers often treated eye diseases among the Nahuatl.^5,24^ A Nahuatl healer, María Salome, treated red eyes by irrigating with cold water, along with an incantation demanding that the “snakes” (conjunctival vessels) stop “mistreating” the “enchanted mirror” (the eyes).^5^

In the Americas, a variety of preparations were used to treat “spots” or “films” on the eyes. These terms generally referred to a corneal opacities. Most of the preparations, such as coconut milk, were used topically. The indigenous recipe of New Spain called for the Mexican poppy to be applied with an unusual solvent (breast milk):^21^ “The Milk, with a Womans Milk that bore a Female, dropt into the Eyes, Cures their Inflammations.”^25^

Among the Huichol of northern Mexico, traditional curanderos (healers) treated trachoma by a ceremony which concluded by sucking blood and pus from the eyes and then spitting this out along with a ceremonial “crystal” which represented the disease.^26^

The capsicum peppers (the active ingredient in modern pepper spray) include both spicy and sweet species native to Central and South America. The spicy varieties contain capsaicin and are also known by the Nahuatl (Aztec) name chilli. Hernández noted of the chilli pepper quauhchilli, that “The Twigs bark’d take off spots and marks from the Eyes.” This shrub “was planted by the Indian Kings in their Gardens.”^25^ One wonders whether the twigs would have been used to scrape the corneas. The 1615 publication based on Hernández manuscripts noted merely that “the cortex of the new sprouts, applied to the eyes [aplicada a los ojos] resolves the clouds [nuebes].”^27^ Even if the material was simply laid on the unanesthetized eye, the mechanical effect of forceful eyelid closure might have removed corneal opacities.

Henry Barham (circa 1670-1726) of England, who resided on Jamaica during the end of the 17th century, described “capsicum peppers” on Jamaica and reported that

Some punish their slaves by putting the juice of these peppers into their eyes . . . and yet . . . some Indians will put it into their eyes before they go to strike fish, to make them see clearer.^28^

Corneal opacities were treated by placing potentially abrasive substances on the eyes. The account of a French voyager to the Caribbean, initially published in 1658, described,

. . . the Eye-stone . . . so cleer, transparent, and smooth: Some of them have red or blewish veins, which give them a very delightful lustre. Being put under the eye-lid, they roll about the ball of the eye . . . they strengthen and cleer the sight, and force thence the motes, or trash which might have fallen into it.^29^

In 1650, on the island of Dominica, one “Mr. du Parquet” was on the verge of traveling to France for treatment of a film on his eyes when a Carib arranged to have his wives perform a cure.^30^ The women “washed his eyes with cotton dipped into the juice of certain herbs. Then they wiped his eyes with their tongues,” and several days later, the “speck” fell off his eyes.^30^

According to the cleric Hernando Ruiz de Alarcón, eye surgery was performed in 1629 by the Nahuatl in Atenango in the present-day area of Guerrero, southwest of Mexico City.^31^ For “dust and superfluities that obstruct my conjured crystal” (the eye), which was also called the “mirror,” the strong herb *tlachichinoa*, which means “incendiary” (and can correspond with *Tournefortia capitate*), was used to prick and rub the eyes until they bled.^5^

In Mesoamerica, liquid exuded by the mesquite or acacia tree, or water in which the shoots had been soaked, was placed in the eyes.^21^ The sap from the bark of the mesquite tree was used by a Nahuatl healer, Marta Mónica, to treat a painful inflammatory eye condition called “xoxouhqui coacihuiztli,” which has been translated as “blue-green pain.”^24^ The color has also been simply translated as “green.”^5^ The sap was collected on a “pearly head” which has been translated as the head of a pin^5^ or the index finger.^24^ The coated finger (or pin) was used to rub the patient’s eye until it bled.^5,24^ The eyelids were then anointed with tobacco, and chicken blood was placed in the eyes.^5,24^ Undoubtedly, some cases of eye pain would have been due to angle closure glaucoma. Did the characterization “xoxouhqui” imply a green pupil? The question is important because many cases of angle closure glaucoma appear to have a green pupil.^32–34^ However, the translations are not explicit on this point. The range of use of mesquite for eye ailments extended northward to the American Southwest, including the Mescalero Apaches, Pimas, and Maricopas.^1^

An early 16th century Franciscan missionary recorded that the Nahuatl doctors “cure well . . . eye illnesses and cut the fleshy growth [cortar la carnaza] from them.”^21^ He specified that “The cataracts [Las cataratas] of the eyes have to be scraped and abraded [se han de raspar y raer]” with a root.^21^ Presumably this condition actually represented ocular surface opacities, such as pterygium. Alternatively, one could “scrape the inside of the eyelids [rasparse lo interior de los parparos]” with an herb.^21^ Bleeding and purging were also used. A branching lesion on the eye (presumably a pterygium) was removed by lifting with a thorn [“Lo enramado de los ojos, se ha de procurar cortar la telilla alzandola con alguna espina”].^21^ Then, mother’s milk was applied to the eyes.

A 20th century Garifuna male doctor of Belize treated a woman with chronic eye inflammation and media opacity with “a plaster made out of egg, vinegar, flour, nutmeg, and alumn.”^35^ In general, for eye inflammation, he treated with eggshells finely ground.^35^

### Mid-continental indigenous ophthalmology

One early 17th century observer in New York declared that “It is somewhat strange that among these most barbarous people, there are few or none cross-eyed, blind, crippled, lame, hunch-backed or limping men.”^36^ However, the Meskwakis were reported to have used liverwort (*Hepatica acutiloba*), presumably orally, to treat esotropia.^1^

Poor vision represented a substantial handicap. In 1850, in the Missouri River valley, Thaddeus Culbertson, who described cases of apparent euthanasia among the natives, had heard of “. . . a blind Crow Indian having been taken to a battle in the hope that he might be killed . . .”^37^

**Figure 1.**
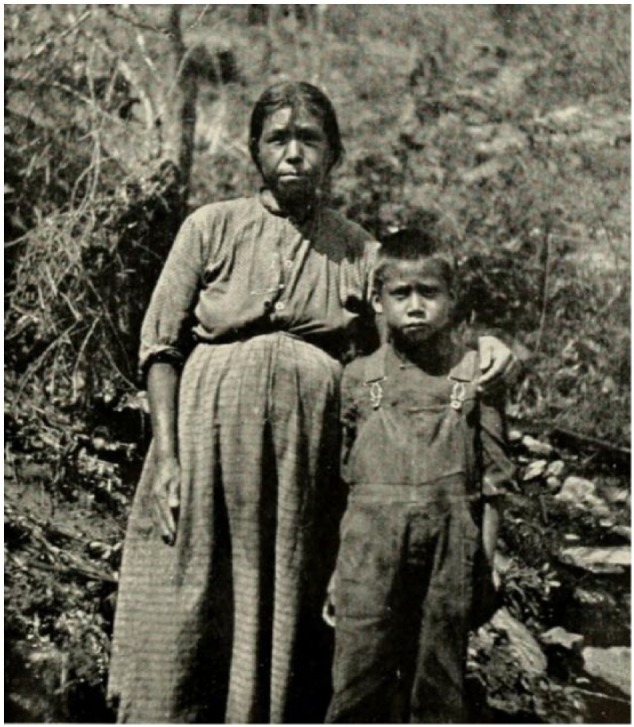
A Cherokee medicine woman with a boy.^38^

Women played prominent medical roles in this region (Figure 1).^38^ A woman’s breast milk was placed in the eyes by the Rappahannocks to treat sore eyes in children, granulated eyelids, or tearing eyes.^1^ The bark of the slippery elm (*Ulmus fulva*) was chewed by the Potawatomis and the product was placed on inflamed eyes.^1^

One observer noted of tribes west of the Mississippi “Affections of the eyes sometimes occur, but I have never known them to attempt cures by any manual operations. When highly inflamed, they blow decrepitated [roasted] salt into them . . .”^19^ Smoke from prairie fires frequently irritated the eyes. A “cold watery infusion for sore eyes” could be made with yellow root or sassafras.^19^

A Comanche woman described rubbing the palpebral conjunctiva until it bled with a blade of rye grass to treat trachoma:

You . . . double it up and turn the eye lid over and scrape it . . . until it bleeds . . . until it finally comes off. It’s the eye lid that causes cataracts because those things rub on the eye and make them sores.^39^

Among the Pawnee of the 19th century, a male doctor poured mother’s milk into a man’s eyes and then wrapped a white cloth around a bullet: “He then carefully scraped the eye, then used his mouth, tongue and suction to remove the cataract.”^40^ Presumably, a corneal opacity was treated.

The Paiute Tribe of the region which became the western United States retained “eye scrapers.”^41^ We do not know the full range of conditions treated, but to treat a man with a “sty,” one woman healer “scraped his eye.”^41^

Smoke was typically considered an eye irritant. The natives of the Great Plains were said to have conjunctival disorders from remaining in smoke-filled tipis.^1^ However, the Navajo placed herbs, including piñon, juniper, sage, and prickly pear cactus, over hot stones to create steam used to treat eye disorders.^1^

The Miami-Illinois natives of the Midwest used “For films on the eyes, a shell of all sorts of river shell fish, burned and pulverized, blown in the eye.”^42^ An additional case relating to both indigenous and European ophthalmology is noted below in the section on lacrimal fistula.

### Northern indigenous ophthalmology

According to a member of the Mi’kmaq of Eastern Canada, the native understanding involved the eye being hollow in the center. Any foreign body getting into the hollow portion would cause blindness. The eye was thought to be attached to the orbital bones by a posterior membrane and by a posterior muscle.^43^

For some eye diseases, the bridge of the nose was pierced without bleeding, somewhat akin to acupuncture.^44^ Father Gideon, an early 19th century missionary to Alaska, recorded that for some eye conditions, a sharpened bear bone was passed through the skin of the eyebrow and the nasal bridge, just missing the eye itself.^44^ The Chugach “Eskimos” treated eye inflammation by bleeding the temples.^44^

Gideon also noted that the Kodiak would fasten a live louse to a hair, lower the insect onto the eye, and “when the insect is observed to have attached itself securely to the film that has formed in the eye, it is then yanked out.” This procedure was repeated until the film was eliminated.^44^ Independent accounts confirm that this story circulated among the early Russian settlers,^44^ but we do not know whether it was true.

The Tlingit of Alaska used a more conventional treatment. Vines were heated, broken, and placed “close to a white spot in the eye” to remove the spot.^45^ Tlingit women sought and cultivated medicinal plants. One woman intended to heat deerberry leaves, cut them to size, and place them on her eye overnight because “in the morning the cataract comes right off.”^46^

Hans Egede, a missionary in Greenland, reported in 1741 that to treat pterygium or pseudopterygium, the Inuits “. . . make a little hook out of a sewing needle and thread it, and then with a knife rip skin or membranes off.”^47^ In 1752, an Inuit woman operated on her husband’s only eye, but the eye was penetrated, and he became totally blind.^47^

**Figure 2.**
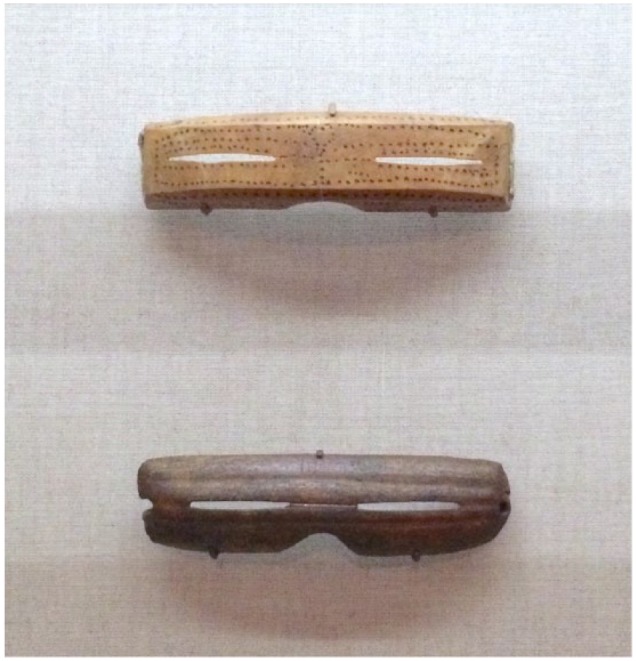
Inuit Snow goggles from Alaska. Made from carved wood, 1880-1890ce (top) and Caribou antler 1000-1800ce (bottom).^48^

Especially in the Northern latitudes, the environmental concerns of snow and smoke blindness were particularly important. Native tribes of Alaska constructed snow goggles to reduce exposure to sunlight (Figure 2).^48^ Among the Tlingit of Southeastern Alaska, snow blindness was common and was avoided by blackening the face or by a headband.^49^ Piercing of the skin without bleeding was used for snow blindness in parts of Alaska.^44^ On Nelson Island, Alaska, human milk was placed in the eyes for snow blindness.^44^ The Chandalar Kutchin and the Upper Tanana of Alaska cut the skin around the eyes for snow blindness.^44^ This procedure was usually performed by women.^44^ Some natives used a knife made of stone.^44^

Among the Pomo tribe of California, myopia was called *ho saha* (fire smoke) because it was attributed to irritation from the smoke of a sweat lodge.^50^ Therapy involved a “sucking doctor” who would suck the smoke from the eye.^50^

Gabriel Druilletes (1610-1681) of France served as a Jesuit missionary in New France.^51^ While on a hunting expedition with the native Montagnais (Inuu) in late 1643, at the age of 33 years, Father Druillettes’ eyes were bothered by the smoke in the lodges.^51^ He began stepping on the feet of others in the camp. Finally, he lost his sight altogether:

The Savages were surprised . . . when they saw that . . . he suffered such pain that his strength failed him. They consulted among themselves whether they should not wrap him up like a parcel, tie him on their sleds, and haul him like the rest of their baggage.

At this, Druillettes laughed and suggested they instead provide a child to guide him. Next,

They held an assembly concerning his disease . . . if he would submit to their remedies, he might be cured . . . Thereupon a woman who was selected to effect the cure, rose from her place and said to him: “Go out of the Cabin, my Father; open thine eyes, and look at the Sky.” . . . this fine oculist armed with a bit of knife blade, or of rusty iron, scraped his eyes till a little humor flowed from them. Never had the poor Father suffered so much. The hand of the operator was not as light as a feather, and she possessed no more skill than science.^51^

Druillettes prayed and asked the natives to do the same. In the middle of the Mass, “a bright ray suddenly opened the eyes of the poor blind man” and he henceforth experienced no further pain or vision loss.^51^ Druillettes’ attribution of his condition to the effect of smoke might be accurate, but given the time outdoors in the snow, a solar keratopathy might have contributed.

### Pacific Islands

In Hawaii, the treatment of a media opacity translated from the indigenous language as “cataracts” (but presumably actually corneal opacity) involved “gently scraping the surface of the cornea with a bit of soft *kapa*, soft *kukae pua’a* grass [*Digitaria pruriens*, itchy crabgrass], or soft sweet potato leaf which was twirled in the fingers across the eye.”^52^ Next, “juice from the *popolo* [*Solanum nigrum*, black nightshade] was dropped into the inflamed eye.”^52^ In addition, “masticated *kukae pua’a* might also be blown from the mouth across the eyeball.”^52^

In babies with “weak eyes,” mother’s milk was placed in the eyes or by blowing across the eyes the masticated leaf of a *lele* banana or the flower of an *‘ilima* [*Sida*].^52^ Coconut milk was also used as an eye wash.^52^ Alonzo Chapin, a missionary in Hawaii in the 1830s, recorded frequent purulent ophthalmia, with opaque corneas, in all ages.^52^

An illustration of the surgery performed in the Pacific comes from the voyage of James Cook to Tonga in 1777. Cook described an operation done by “the natives of these islands”:

. . . a woman was dressing the eyes of a young child, who seemed blind; the eyes being much inflamed, and a thin film spread over them. The instruments she used were two slender wooden probes, with which she had brushed the eye so as to make them bleed.^53^

## Eye Diseases and Treatments

### Return to Europe for care

Early European settlers sometimes returned to Europe for eye care. Christopher Columbus suffered from an eye ailment while in America. He was noted to have a disorder affecting the use of his legs on his first return from America in 1493. He experienced fever and vision loss on his second voyage to the Caribbean in 1494. On his third voyage in 1499, he experienced arthritis (“*gota*”) followed later while off the coast of Venezuela by bloodshot and painful eyes (which he described as “*rompieron de sangre y con tantos dolores*”).^54^ Columbus was bedridden during much of his time on Jamaica in 1503 and 1504 until his death in Spain in 1506.^54^ The modern differential diagnosis includes reactive arthritis with uveitis.^54^

Other early eye patients also returned to Europe. The oculist Dawbigney Turberville (1612-1696) of Salisbury, England, treated the eye condition of a woman who returned from Jamaica. Turberville had expertise:

In knowing when the connate Cataract is fit to be Couched, in having a steady Hand, and skill to perform that Operation . . . his Fame brought multitudes to him . . . even from America . . . a young Woman coming out of a Boat, who as soon as she had set foot on Land, kneeled down and said these words . . . Oh Lord God, I pray thee, that I may find Dr. Turbervile living, and not make this long Voyage in vain . . . .She went to Salisbury, and . . . was perfectly cured; but her Joy did not last long, for in her return to Jamaica, of which Island her Husband was one of the principal Inhabitants, she died of the Small-Pox in London.^55^

Other voyagers were more fortunate. The sovereigns of England and France held that their touch could cure the disease known as the “king’s evil,” which modern historians believe was caused by tuberculosis. Obviously, this cure was not available in America. An English writer of 1684 learned from one Mr Doublebrook that

a Woman who came from Virginia, whose Nose was almost eaten away with the Evil, and her Eyes consumed with that Humour, she being brought by him to the King to be Touched, immediately received benefit thereby, and returned to Virginia since, and as a token of Thanks to him, she sent him a Pair of Gloves, with a Letter, wherein she certified him that she was recovered from her Disease . . . .^56^

### Eye inflammation medical treatment

Before the invention of the ophthalmoscope and slit lamp, all eye inflammations were generally diagnosed as ophthalmia^57^ or colloquially “sore eyes.” “Sore eyes” constituted 3% of all medical diagnoses recorded by Thomas Reide, a British Army surgeon near the St. Lawrence River from 1776 to 1787, and with an average regimental strength of 511 soldiers,^58^ we calculate that approximately 18% of the soldiers would have experienced this condition during a given 11-year period of service.

Numerous systemic diseases could produce eye inflammation. Syphilis, even today a cause of uveitis, was probably introduced to Europe with the return of Columbus. Medical remedies from the New World, “Sassafras and Sarsaparilla” were used in Europe for “venereal disease.”^59^ Yellow fever epidemics were common through the end of the 18th century. This disorder involved “. . . head-ach . . . [in] the lower part of the forehead, the eye balls and their sockets” and “a remarkable inflammation in the tunica adnata [conjunctiva] . . .”^60^

Medical use of mother’s milk was not restricted to the indigenous cultures. According to physician John Tennent (circa 1700-1748) of Williamsburg, who wrote a collection of home remedies, “Common Sore Eyes may be cur’d by washing them with Breast Milk, warm Sage Tea, or with Rose Water . . .”^61^

Therapeutic phlebotomy was a mainstay of European medical treatment during this period. English physician Hans Sloane described multiple ophthalmic patients from his time on Jamaica in the 17th century. A 45-year-old cook “given to Drink” chronically had “inflam’d swell’d Eyes” which Sloane treated with “bleeding, purging, and blistering.”^24^ Sloane recounted that a turner cutting down a “Mansanillo Tree” had “the Milk spurted into his Eye” rendering it “sore and inflam’d” and “the Eyelids were so swell’d and glu’d together . . . that he could not open them.” Sloane ordered bleeding, purging and “order’d him to wet his Eye very often in cold water . . .” Irrigation remains a key part of the modern treatment for a chemical injury. The patient was “cured” in 3 days.^24^ Other authors agreed that if the “milk” of the “Manchenillo-Trees” fell “into the eye, it will cause an insupportable inflammation, and the party shall lose his sight for 9 days.^29^ The “Indians” of the Caribbean used the tree to “anoint their poison’d Arrows for Wars.”^62^

Tobacco (*Nicotiana*), a plant native to the Americas, was held by some to soothe the eyes. Limited medical use was documented among the natives. For instance, the Maya used tobacco to treat sore eyes.^1^ Many Native tribes, such as those in the Carolinas, smoked the plant.^1^ Some Native groups ingested tobacco or applied the juice or leaves externally.^1^ The European cultures were more enthusiastic about tobacco’s health benefits. One 1722 advertisement for “Cephalick and Opthalmick Tobacco” made from “the very best Virginia Tobacco” always

. . . Restores ancient Sight, & preserves young Eyes, that by the Use of it, Persons . . . never come to wear Spectacles . . . It brings away Rheums & Humors that cause . . . Sore, Weak, Watery, & Dim Eyes . . . .^63^

Tuberville of England also “generally prescribd to all, shaving their Heads and taking Tobacco, which he had often known to do much good, and never any harm to the Eyes.”^55^ But not everyone was convinced. A 1716 tract advised that tobacco from “warm Virginia, or Bermuda’s fields fits only for your slaves; rejects the bane, which hurts the eyes; and stupefies the brain.”^64^

Sloane wrote that in Jamaica “Sore Eyes, inflam’d, and painful, are very ordinary here.” He observed “sometimes a Cataract to begin with an Inflammation there.” He recommended bleeding, purging, and “blistering in the Neck.” This treatment was successful, unless the inflammation came from “much Venery,” which had to be cured by “Abstinence” and medicines, such as “I used outwardly to drop into the Eyes . . . Rose-water, Lapis Calaminaris, & Tutia.” These medicines were recommended by others during this period.^65^ He also applied “lime juice,” which was not a classic English recipe.^24^

Sloane maintained a lifelong interest in ophthalmic conditions. He eventually acquired and published the recipe for an “ointment for sore eyes,” consisting of “prepared tutty” (an oxide of zinc), “blood stone” (a gem spotted or streaked with red), “succotrine aloes in fine powder” (a plant originally from the island of Socotra, in modern Yemen), and “prepared pearl,” mixed with “viper’s fat.”^66^ This recipe continued to be published in America even at the end of the 18th century.^66^

Some native plants used medicinally by the indigenous peoples were adopted by the Europeans. A Dr Chisolm of Grenada in 1790 learned that the “Arrowawks” near Demerary in Dutch Guiana used a plant which the “Indians” called “Akuserunee” and which he called the “eye-root” was effective in “curing inflammations of the eyes.”^67^ One places one drop of the plant’s juice in the eye daily for 4 days. Chisolm used the remedy successfully in 2 patients in Grenada: “a porter negro, who had nearly lost the use of his eyes by the violence of the inflammation, the other a sailor . . .” Chisolm proposed the name *Bignonia Ophthalmica*.^67^

For fever with headache and “inflammation in the Eye,” Tennent advised to “bleed 10 Ounces . . . purge with the Decoction of Mallows, and 3 Spoonfuls of Syrrup of Peach Blossoms” and apply a “Poultis” of “Sage, Wormwood, and Rue” with nutmeg, and “Blister” near the site of the pain.^61^

### Corneal opacity: nonsurgical cures

Corneal opacities in this era undoubtedly had multiple causes, such as pterygium, corneal edema, band keratopathy, and corneal scarring, from trauma or prior infection.

Smallpox survivors were frequently left with scarring of the cornea or face. John Adams recorded the effect of smallpox:

There is a poor Man . . . is the most shocking sight . . . he is no more like a Man than he is like an Hog or an Horse—swelled to three times his size, black as bacon, blind as a stone.^68^

Barham on Jamaica knew of islanders who successfully used “a sort of thyme” locally called “eye-bright,” “to take off the spots or films on the eyes that have come after the small-pox . . . by only dropping the milky juice into them.”^28^ It is not clear whether this plant was the same as *Euphrasia*, also called eyebright, which was a long-standing eye remedy in Europe.^69^ The traditional eyebright (*Euphrasia*) was discussed as a remedy for eye inflammation in the *Medicina Britannica*, republished in Philadelphia in 1751 by Benjamin Franklin. The editor of this American edition, botanist John Bartram of Philadelphia, noted that he had never seen *Euphrasia* growing in America, however, he had tried to sow it, and others had erroneously called other American plants by this name.^70^ Bartram might have had a particular interest in this type of medicine, as he wrote to Franklin, “My eye sight fails me very much,” and that he was turning over matters to his son for that reason in 1771.^71^ Franklin responded by mailing spectacles to Bartram.^72^ Whether *Euphrasia* really grew locally or not, medicines based on the plant were advertised. For instance, in the 1770s, physician John Sparhawk (1730-1803) of Philadelphia sold “Essentia euphragiæ,” to be given topically, which “. . . removes all Specks, Films, Mists, and Suffusions, cures all Runnings of watery and foul Humours . . .”^73^ Sparhawk advised in cases of “. . . Inflammation or Redness in the Eyes, to take off 10 or 12 Ounces of Blood.”^73^

Tennent, the Virginia physician, recommended,

In case a film shou’d grow over the Sight of the Eye, occasioned by a Blow, a sharp Humour, or other Accident, you may take it off . . . Dry Humane Dung in the Sun . . . and having reduced it to a very fine Powder, blow it thro’ a Quil Two or Three Times a Day into the Eye . . . .^61^

We learn more about corneal pathology from the settlement established when Lutherans from Salzburger emigrated to establish Ebenezer, Georgia, in 1734. One-third of the settlers died in the first 2 years.^74^ Two 20-year-old “blind boys” born in the early days of the settlement cooked, fetched water, and cleaned dishes under the supervision of a preceptor.^75^ In 1749, a “rich and well-known planter” near Charleston with poor vision for 2 years requested *essentia dulcis ad oculos*, an expensive eye ointment containing gold dust, which had already helped him to recover vision in one eye. The settlement pastor, Johann Martin Boltzius (1703-1765), declined to provide further ointment and suggested that the planter travel to visit the settlement’s doctor or surgeon.^76^

Boltzius himself suffered a variety of symptoms which we might propose as coming from at least 2 conditions. Historians have long believed that Boltzius developed malaria at some point. As early as October 1736, Boltzius suffered from “severe dizziness and cramps.”^77^ At that time, he also wrote of “the fever that has befallen all of us.”^77^ He used “essentiam antihypochondriaca or spleen opening essence,” “pillulae purgantes,” and “balsamus cephalicus nervus” for these conditions.^77^ The medication for the spleen suggests that perhaps he already had malaria.

Malaria can result in retinal hemorrhages and edema,^78^ but we believe that Boltzius suffered from an unrelated eye condition, possibly ocular rosacea. In the Winter of 1753, Boltzius began to get “inflammation” in the left eye.^75^ The eye “inflammation” was initially accompanied with “a head cold” in which “the head was full of fluid.” By May of 1754, the inflammation had subsided, and Boltzius described his left eye as having “a thin film over the eyeball,” and later as “covered with a white film.”^75^ Boltzius wrote that the worse eye was “just as sensitive as the healthy one,” perhaps to indicate that light perception was still present. Unfortunately, his surgeon Johann Ludwig Mayer (1715–1763) was too “timid” to operate.^75,77^ Boltzius was unable to write in his journal for some time.^75^ By the spring of 1755, medicines and “glasses” had been sent from Europe to help the pastor cope with his eye condition.^75^

By 1759, symptoms consistent with cerebral malaria were prominent: he had “dizziness,” both eyes were “cloudy,” he felt “pressure in them,” with his head “full of fluid.”^75^ He had occasional hearing loss, with “ringing and roaring of the ears” (tinnitus).^75^ His physician Christian Ernst Thilo (1708-1765), trained in Halle, recommended exercise and moderation.^77^ Boltzius submitted to bloodletting 4 times yearly. Thilo judged his blood to be “proper consistency, but every time a whitish film, like a powder, covers over what is collected in the bowl against the edge”^75^ (possibly due to leukocytosis). As the intended delivery of medicines to the settlement had not arrived for several years, they again requested medicines, including *essentia dulcis*.^75^ Thilo deemed treatment futile, and Boltzius feared that medicines might damage the right eye. His right eye was normally “clear” but became “cloudy like a glaucoma” when he had “catarrh” in his “nasal passages.”^75^ Glaucoma in much of the European ophthalmology literature of the 18th century had begun to specifically represent angle closure glaucoma.^32–33^ However, in a colloquial sense, Boltzius probably simply meant that the cornea appeared lighter. He also noted “rosiness or redness” of the face.^75^ In September 1762, he had an episode of fatigue, nose bleeding, fever, coughing.^75^ Herpetic keratitis is in the differential diagnosis and is known to be associated with malaria, although it usually occurs unilaterally. Rosacea could explain the facial skin changes, conjunctival injection followed by a corneal pannus over the left eye, and intermittent keratitis in the right eye. Cerebral malaria might account for the leukocytosis, vertigo, and tinnitus.

### Corneal and conjunctival surgery

Surgery apparently for corneal opacity was performed in early New Spain (Table 1). Fray (Father) Lope de Cuellar had poor vision after a fall at the Temple of Yanhuitlán in Oaxaca in 1601.^79^ One eye had “a cloud which covered the entire pupil [una nube, que le cubría toda la niña],” perhaps a traumatic cataract, and was deemed untreatable.^80^ The other eye was found to have “a fleshy or raised opacity [una carnosidad o catarata levadiza],” which might have been a preexisting corneal lesion, such as a pterygium. The surgeon of the monastery picked at the fleshy material with a needle until Lope exclaimed that he could see the Rosary and thanked God. The surgeon said that he had missed a little tissue and continued to use his instrument. Unfortunately, he ruptured the eye (“reventó el ojo”). Although the patient lost all vision, he again thanked God with equanimity.^80^ Even centuries ago, the perfect was the enemy of the good.

**Table 1.** Surgeons who treated corneal opacities.

Year	Surgeon: comment
1611	Francisco Drago in Mexico “removed pterygia [extirpar carnosidades]”^4^
1761	“Doctor Hugh Tomb . . . lately arrived from Ireland . . . was educated at the university of Edenburgh, and has practised in Physick, Surgery & Midwifry, for three years in Ireland . . . he engages to cure . . . filtrims on the eyes, commonly call’d Pearls . . .” Philadelphia^82^
1771	James Graham: A woman from Bound Brook, New Jersey described Graham “curing my son of a film over his right eye, occasioned by the small-pox, so that he has now recovered his sight perfectly”^83^
1773	Francis Mercier. “. . . undertakes to cure . . . Purls, Redness . . .”^84^
1774	Anthony Yeldall: “An Eye Powder . . . for taking off specks, films, webs, &c. (if not mixt with the horny or outward coat) of the eye”^85^
1776	Frederick William Jericho: “Remedy for the Gravel, . . . Films, Spots, Weakness of the Eyes”^86^
1778	Frederick Carle Pflag (Friedrich Carl Pflug) Philadelphia, New York^87,88^
1791	Peter Degravers: Jamaica^89^
1793	Mason Fitch Cogswell: Hartford. One Miss Williams of Brooklyn, New York requested “the removal of a film or an opaque tumor from the eyes”^90^
1799	“Dr. G. W. Adlersterren, from Lancaster, Formerly Surgeon in the Hospitals of the Emperor of Germany . . . He undertakes to cure . . . Film over the Eye . . . He practices Surgery and Midwifery.” Alexandria, Virginia^91^
	William Baynham: Essex, Virginia. “I had operated on your Servant Tom’s Eyes . . . The tumor in the left Eye is . . . incurable; and a growing film in the right threatens to overspread the transparent Cornea”^92^
1801	Charles F Bartlett, Newport^93^: “. . . an experienced operator in the eyes, by removing blindness occasioned by cataracts, films, &c”^94^

**Figure 3.**
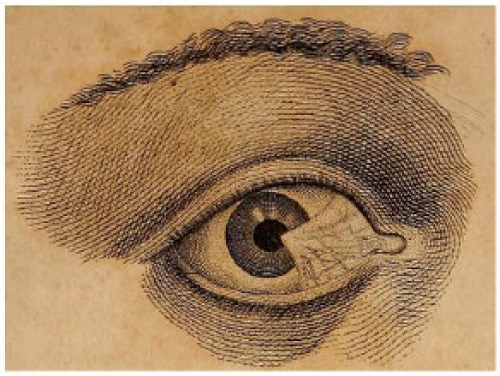
Pterygium (or unguis), in *Elements of Surgery* by John Syng Dorsey (1813), which contained many of the teachings of Philip Syng Physick.^81^

Well-established oculists might have performed corneal surgeries in the English colonies (Figure 3, Table 1).^81–94^ Anthony Yeldall of England published the following testimonial in 1775:

. . . I, Mary Irons, of Queen’s county, Maryland, was afflicted with blindness for many years . . . Doctor Yeldall . . . brought me to the sight of one eye in a minute’s time, by taking off the film . . . .^85^

A New York advertisement from 1778 read,

Frederick Carle Pflag, Surgeon of the Hessian Grenadier Battallion De Lengercke . . . is ready to serve . . . as an Oculist . . . Those who have the misfortune to be wall-eyed, he will relieve by operation, by taking out the skin of the dark eye, in the newest and best fashion, without any pain, so that they may have immediately their sight again . . . He may be found in the camp of said battalion near Haerlem . . . .^88^

At the time, a “wall-eye” often referred to having one light and one dark eye. As Pflag proposed operating on the darker eye, perhaps he was resecting a conjunctival nevus or melanoma.

Peter Degravers was a colorful surgeon who advertised in Jamaica in 1791.^89^ His ophthalmology text noted, “The disorders of the cornea are very common. The albugo and spots which grow on this membrane are produced by inflammations very ill attended to.”^95^ Degravers noted that smallpox was a frequent cause of corneal scarring. He continued, “The Pterygium is a rising, fleshy membrane, which . . . extends slowly from the conjunctiva to the cornea . . .”^95^ He added, “The pterygium, as well as an infinity of tumors, which grown under the eye-lids, may be extracted without the least danger to the organ . . .”^95^

### Angle closure glaucoma

Angle closure glaucoma was probably more common historically when cataract surgery was not widely available. In the pre-ophthalmoscopic era, we might suspect that patients were having an attack of angle closure when they experienced acute or subacute loss of vision, pain, a red eye, mydriasis, and an anteriorly prominent lens. Indigenous Mesoamerican healers might have treated angle closure glaucoma, as described above.

Sloane recounted the 1688 story from Jamaica of “Dr Rook’s Wife,” 35 years old, who

lost entirely the sight of one of her Eyes, and with the other could very hardly perceive any thing . . . The Pupil of one stood always wide open, and that of the other on looking at distance or near Objects, scarce alter’d, contracted or dilated its self . . .

Sloane “order’d her to be Bled by Cupping with Scarification in the Shoulders, to be blister’d in the Neck, to be purg’d . . .” He also ordered thyme, sage, rosemary, and millipedes, all by mouth. The use of millpedes was a favorite therapy of Robert Boyle, a friend of Sloane’s. Sloane reported that she eventually could “read Bibles of the smallest Print.”^24^ We cannot be certain of the diagnosis in this case of asymmetric myrdriasis. The vision loss seems more extreme than would be expected with an Adie’s tonic pupil. Sloane does not describe the pain and redness anticipated in angle closure glaucoma, but the description could be incomplete.

James Graham was the only practitioner who published the term “glaucoma” in the region during the 18th century. He lectured on glaucoma at the College of Philadelphia in 1773. Glaucoma had transitioned from the nonspecific concept of the light-colored eye of the ancient Greek authors by the 18th century to more specifically represent, in many works, the light green or gray pupil sometimes seen in angle closure glaucoma.^32–34^

For surgeon Peter Degravers, “the glaucoma is an opacity of the vitreous body” which turns “a green color.”^95^ His description of a separate vitreous disorder (which he did not name) resembles angle closure glaucoma. The patients have eye pain, with “the pupil more dilated than ordinary, contracting but very little,” severe vision loss, occurring in both eyes simultaneously or sequentially.^95^ This pathology eventually could be observed “terminating always in an incurable glaucoma.”^95^ He did note that the expanding vitreous “brings the crystalline lens forward, and presses on the posterior part of the iris.”^95^ Degravers noted that the disorder could sometimes be cured, but he did not indicate how.^95^

John Collins Warren at Harvard Medical School performed lens extraction and even a crude limited vitrectomy for what he called “Dislocation of the crystalline lens.”^96^ (His middle name is written to distinguish him from his father, also John Warren of Harvard.) These cases are consistent with angle closure glaucoma. Cataract extraction for angle closure glaucoma was performed by William Mackenzie of Scotland in 1833.^33^ Thus, lensectomy for angle closure glaucoma was performed in America well before the publication of Mackenzie’s text.

John Collins Warren wrote, “About the year 1806, there occurred . . . the first case I had ever noticed, or seen described, of spontaneous dislocation of the crystalline lens.”^97^ The case involved “. . . a man of about forty, who was suddenly attacked with violent pain in his right eye, without any external cause.”^96^ Later, Warren added to the description of

. . . a gentleman who had a very severe inflammation of one eye . . . the pupil neither dilated nor contracted . . . the slow progress of the disease, the opacity of the crystalline lens, its prominent position, and the semi-globular projection of the eye, led me to suspect that the crystalline lens was misplaced, and made a pressure on the iris.^97^

He also described the lens position as “near to the cornea”^96^ and recounted,

With the advice and aid of my father, I performed the operation of extraction by first opening the cornea, enlarging the aperture with the scissors, and then dissecting the crystalline lens from its adhesion to the iris. The patient was thus relieved from suffering, but, of course, could not recover his vision.^97^

John Collins Warren described a number of cases of “lens dislocation” in 1816. One “Mr. G. was attacked with a severe pain in one of his eyes.”^96^ Warren noted “an immoveable pupil, in which lay the crystalline lens having its edge projected through the opening.”^96^ Warren offered lens extraction, but the patient declined, and was chronically troubled with pain until his death.^96^

Also under the heading of “Dislocation of the Crystalline Lens,” Warren added that a lady with “inflammation of the eye” had an “immoveable, and slightly dilated” pupil, with monthly recurrences of eye pain.^96^ Warren judged her symptoms to be too mild to offer lens extraction.^96^

In another case under this heading, Warren described a woman with sudden right eye pain, vision loss, inflammation, a tender eye, and a fixed dilated pupil. Warren “made a puncture in the cornea, introduced a probe, and found the crystalline lens pressing . . . on the cornea.”^96^ Warren then enlarged the wound with scissors and extracted the lens. This maneuver provided only temporary relief, but when the pain returned, Warren noted “the vitreous humour was projecting through the wound in the cornea.”^96^ When removing the prolapsed vitreous did not relieve the pain, Warren “passed a cataract knife into the cornea made a large crucial incision, gave vent to a considerable part of the discoloured vitreous humour and thus relieved her.”^96^ Warren’s description is consistent with a successful vitrectomy for malignant glaucoma.

Later, Warren added,

A number of years ago, I was called to a lady who had spontaneous dislocation of both lenses, known by dilatation and immobility of the pupil, prominence of the iris and cornea, and the severity of pain . . . I proposed the operation of extraction.^97^

The patient’s friends sought a second opinion. A second surgeon managed the case conservatively, and the patient lost both eyes.

Of course, true dislocation of the lens can occur, with trauma, Marfan syndrome, pseudoexfoliation, or other conditions. But this large number of spontaneous cases in Warren’s practice, and the specific clinical descriptions, suggest angle closure glaucoma.

The lectures of Philip Syng Physick, who was appointed to the medical staff of the Pennsylvania Hospital in 1794, noted, “Cataract . . . is produced by an opacity of the crystalline lens or its capsule. It is sometimes accompanied with pain over the eye . . .”^98^ Pain accompanying cataract can be due to angle closure glaucoma, chronic uveitis, and phacolytic or phacoanaphylactic glaucoma.

Under the heading “Tapping the Eye” (paracentesis), Physick’s nephew John Syng Dorsey recorded,

Dropsy of the eye, or hydrophthalmy, sometimes renders it necessary to evacuate the aqueous humour, accumulated in too great quantity . . . if the eye-ball continues to augment in volume, and protrude from the socket, it becomes necessary to open the eye by a surgical operation . . . In several cases I have punctured the cornea by inserting the point of a sharp cataract knife through it, the evacuation of the aqueous humour although not sudden, was amply sufficient.^81^

The cases with protrusion could be an anterior staphyloma or descemetocele which could have originated with an infection which weakened the cornea or cases of angle closure or other types of glaucoma which had been poorly treated.

Dorsey continued, “Dr. Physick from an idea that gutta serena is in some cases occasioned by pressure upon the retina and optic nerve from an over secretion of aqueous humour has punctured the eye with a view to relieve this affection.”^81^ The procedure had mixed results. The term “gutta serena” suggests that the paracentesis was performed for elevated intraocular pressure in a normal appearing eye.

### Lacrimal fistula

In the era before antibiotics and effective surgery, dacryocystitis would frequently produce a lacrimal fistula. The condition could be chronic, or associated with cellulitis, and other severe complications. Available remedies were rudimentary (Table 2).^99–100^

**Table 2.** Management of oculoplastic conditions, including lacrimal fistula.

Year	Surgeon and disorder
1771	James Graham, an itinerant. Patient Daniel Turner of Newark acknowledged “the cure . . . of a fistula lacrymalis of my left eye . . .”^83^
1774	Samuel Clossey, in New York, lectured on anatomy relevant to lacrimal fistula
	Francis Mercier, an itinerant. “Undertakes to cure . . . Fistula Lacrimalis”^84^
1776	Frederick William Jericho, an itinerant. (Likely medical) remedy for “Fistula Lacrimalis.”^86^ Performed an enucleation (see text)
1783	“Samuel Prentice [Prentiss] . . . in . . . Worcester . . . Cutting for the Fistula Lachrymalis”^99^
1787	“Doctor Stoddard” resected an “atheroma,” possibly a dermoid cyst, in Hudson, New York^100^
1791	Peter Degravers. “. . . a safe operator for . . . Fistula lacrymalis”^89^
1795	John Warren lectured on surgery for lacrimal fistula in Cambridge

Some idea of the severe disability in this era comes from the 1663 letter of Abigale Montague of Hadley, Massachusetts to John Winthrop, the Younger (1606-1676), Governor of the Connecticut Colony. Montague’s 7-year-old daughter had “a thistilow [fistula] just by the corner of her eye by ye side of her nose.”^101^ (Fistula was sometimes written “thistolow.”)^102^ For the first year, she had “a clear watery rume [discharge], twickling out of her eye, and it was so hot that it would make the skin to come off.”^101^ Eventually, the discharge thickened, and the dacryocystitis occluded the eye: “it gathered betwixt her nose and her eye to a great swelling in so much that her eye was blinded up,” followed by facial cellulitis:

all that side of face & head was so swelled & in great pain that we were afraid she would have died: & then it broke on the side of her nose by her eye & run a pretty deal.

She eventually developed “ye purples [purpura],” possibly from sepsis. They dressed the opening of the fistula and after they placed a sponge on the fistula, it bled. They placed water in the fistula and “ye water came out at her nose & run down her throat in so much that it had liked to have strangled her.” Her overall condition deteriorated: “She was formerly very full but now lean & poor . . .” They placed “bees wax & butter” on the fistula, but it “would not stick” so they had to secure a cloth over the opening. The girl did her best to cope: “She is naturally very lively although she undergoes a great deal of misery.” Her mother requested to know if “sisapharilla [sarsaparilla] root” or “English barley boiled with cole herbs be good for her.”^101^

Sarsaparilla indicates *Aralia nudicaulis* but has been occasionally used to describe other American plants.^1^ Some native tribes made a lotion of the root to treat sore eyes.^1^ It might seem odd that the mother would ask the colonial governor a medical question. However, Winthrop wrote 2 scientific articles and mentioned the “Bark of a Tree” in Nova Scotia and New England, with “a liquid matter like Turpentine . . . of a very sanative nature, as I am informed by those, who . . . have often tried it.”^103^

**Figure 4.**
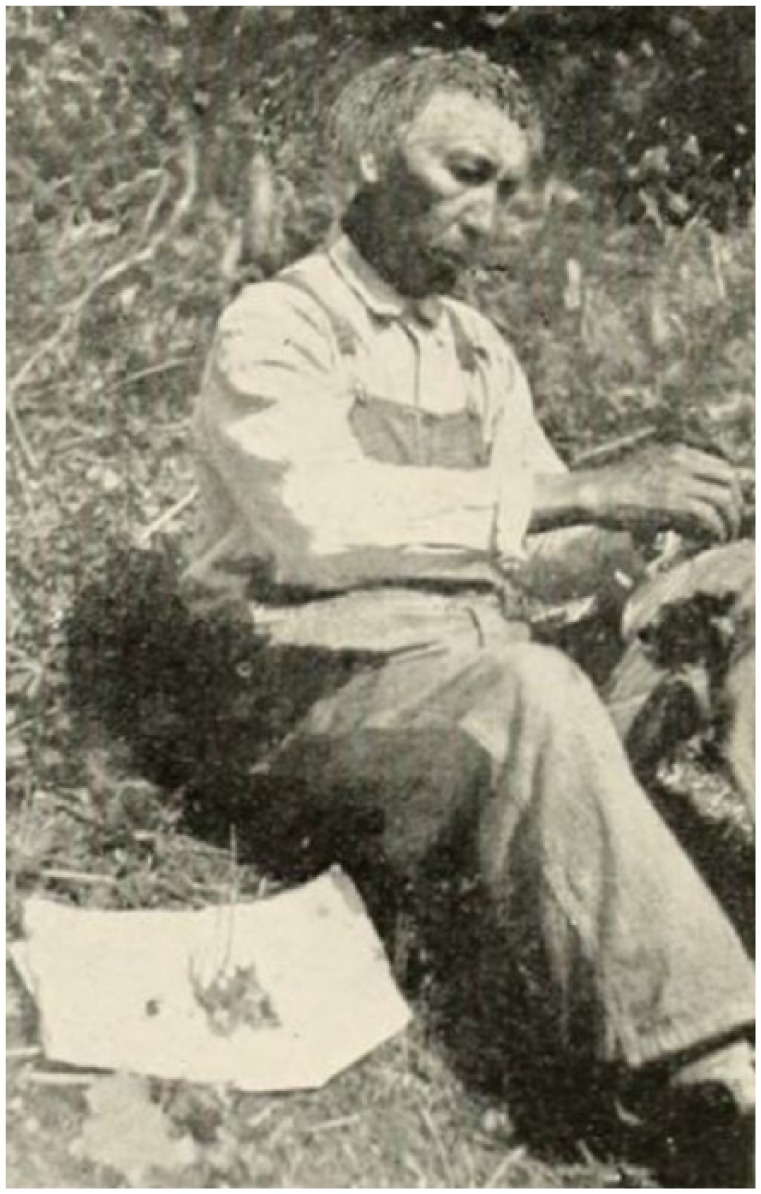
A Cherokee medicine man wrapping his “simples” (medicines) in a white cloth.^38^

Antoine-Simon Le Page du Pratz (1695-1775) of France recorded his life in the early 1720s near Fort Rosalie, in the Louisiana territory. He experienced a “fistula lacrymalis . . . which discharged an humour, when pressed . . .”^104^ The French surgeon M St. Hilaire, who had practiced for a dozen years at the Hôtel Dieu at Paris, recommended cautery (“use the fire for it”) before the nasal bone “would become carious.”^104^ Before du Pratz could decide whether to have the operation, the Natchez leader (“Great Sun”) happened to see the swelling on the eye and sent a native healer. This physician pressed his “simples” (medicines) together in a ball, and placed the ball in water, in which du Pratz soaked his eye twice daily for 8 to 10 days (Figure 4).^38^ The “fistula” resolved completely.^104^

In 1791, Peter Degravers advertised in Kingston, Jamaica, “Doctor Degravers is a safe operator for the Cataract and Fistula lacrymalis.”^89^ His 1788 treatise clarified that the term “fistula lacrymalis” was used in this period for any dacryocystitis (even those that had not yet developed a true fistula to the skin surface), whereas those with a true fistula were described as “a complete fistula lacrymalis.”^95^ For simple cases of dacryocystitis, Degravers recommended nasolacrimal massage (“press on the bag”) and irrigation (“make several luke-warm injections through one of the lachrymal points”).^95^ Second, Degravers mentioned the “silver probe” of Anel, which was introduced through the “superior lacrymal point” (upper canaliculus) into the nose.^95^ When learning to find the probe entering the nose, he advised the operator “to begin on dead bodies.”^95^ He also described passing the probe into the nose, through the “inferior orifice of the ductus ad nasum” (the valve of Hasner) and into the lacrimal sac.^95^ When probing and injections fail, the next step is “opening the bag by an incision.”^95^

At Harvard University, a student of (the elder) John Warren recorded in 1794 that

The operation for the fistula lachrymalis is performed upon the os unguis, this being a bone no thicker than a wafer—Often the instrument has passed a bougie [a flexible surgical probe] inserted for 6 or 8 weeks which prevents the bone from closing & filling up the aperture.^105^

This language is similar to that of English surgeon Samuel Sharp.^106^

### Orbital pathology and enucleation

**Figure 5.**
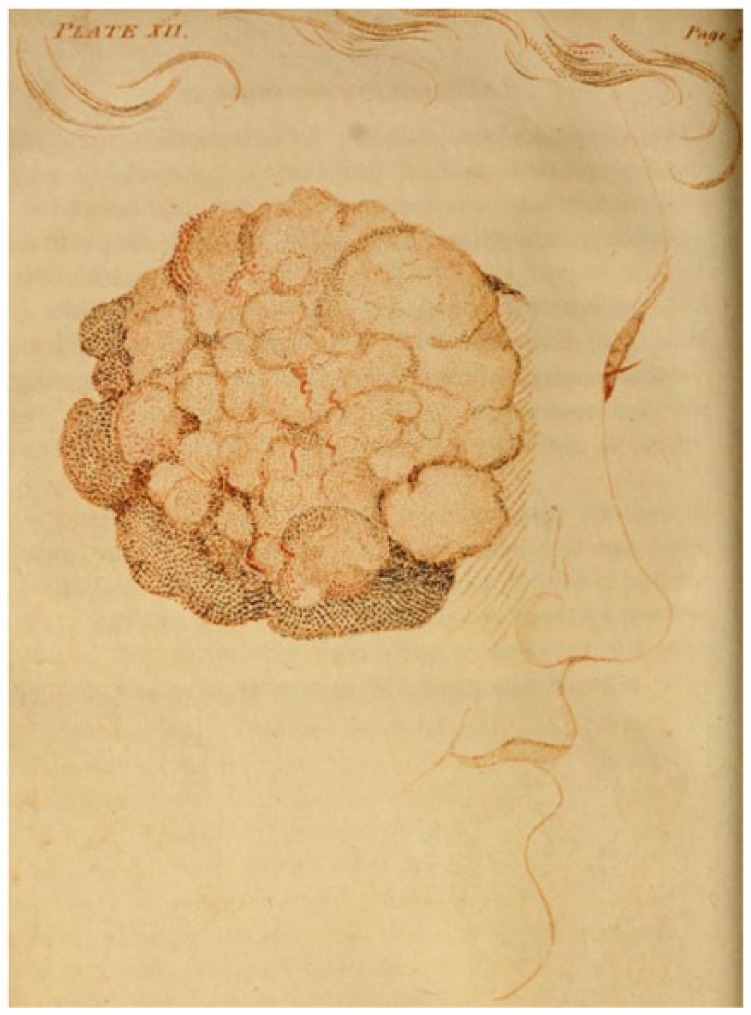
Carcinoma of the eye or fungous tumor.^81^

Some orbital masses were treated surgically in the New World (Figure 5).^81^ After his return to England in October 1776, surgeon Frederick William Jericho advertised several cases of orbital pathology he treated in the Caribbean. Given the times we know Jericho to be in Europe, these treatments would have been performed between 1771 and 1776. This is the first surgical enucleation identified in the New World (Table 2):

. . . Robert Dyett, Esq; of Montserrat, who had an Aneurism in his right eye, which extended the globe to such a degree, that he was in convulsions and the most excrutiating pain day and night . . . Doctor Jericho, who took out the whole globe of the eye, and in three weeks time made a perfect cure of him . . . .^107^

In another case, Jericho’s method of cure is unknown:

. . . in the Island of Antigua, he performed a very surprising cure on a Lady of a fungus cancer, which had afflicted the right side of her nose and eye-lids; the disorder had been of ten years standing; she had been under the care of many of the faculty, without receiving any benefit, and was thought incurable . . . .^107^

In 1787, a “Doctor Stoddard” resected a congenital “incysted tumour called . . . an atheroma, from the matter contained in the cystis, or bag, resembling and being of the consistence of a soft pudding.”^100^ The mass was “cut out of his [the patient’s] forehead, right eye-brow and lid” and was “covering the orb of the eye and part of the temporal artery, both of which he [Stoddard] avoided cutting, though the adhesion was considerable.”^100^ The description is consistent with a dermoid cyst. The 41-year-old patient, John Brassee, of Livingston, New York, had refrained “from having it extirpated from a belief that it would be attended with sudden death.”^100^

### Gutta serena

Before the invention of the ophthalmoscope in 1850, many posterior segment conditions resulted in loss of vision which did not respond to couching and in which the eyes appeared normal. These conditions would include primary open angle glaucoma, macular diseases, retinal detachment, retinal vascular diseases, optic neuritis, etc, and were sometimes known by the Greek terms amaurosis, if severe, or amblyopia, if mild.^33,57^ However, in the English colonies, the Latin term *gutta serena* seemed to prevail through the 18th century.

Sloane recounted that “Henry, a Negro, Overseer . . . much given to Venery [sex]” developed poor vision at near and far. His eyes appeared normal. Sloane recommended he be “very Chast” and “cup’d and scarified in the Shoulders, blister’d in the Neck.” He also prescribed millipedes by mouth. Eventually, the Overseer recovered his sight.^24^

Following a “belly ache,” another overseer became “usually blind.” Sloane treated by bleeding, purging, and blistering “to remove the Obstruction of the Optic nerve . . . his Eyes having no outward visible Disease.”^24^

One 55-year-old man with jaundice, belly pain, and mental status changes “lost his Sight quite . . . although his Eyes look’d well and without blemish, for which I order’d him to be bled. I blister’d him in the Neck likewise . . .” Sloane noted that “This sort of Gutta Serena goes off in some Days, and they recover their Sight . . .”^24^

In areas where couching of cataracts was unavailable, whether one believed a case could be cured by couching was unimportant, and the decision to label a case “gutta serena” was not critical. Therefore, before couching entered the mainland colonies, “gutta serena” was sometimes used simply to mean poor eyesight. In the 1750s, the Pennsylvania Hospital reported cases in which both “Eyes disordered” and “Gutta Serena” were cured.^108^

James Graham understood that gutta serena was classically viewed as incurable, but he claimed to be able to cure it. Patient Theophilus Pierson of Newark testified,

after being afflicted with a severe and constant headach, and almost total blindness (my case being the gutta serena) above six years, I have . . . recovered my sight, and am perfectly cured of my headach, thro’ the means that Dr. Graham has applied . . . .^83^

### Eye trauma

The penalty for causing eye injury in the American colonies depended on the status of the injured party. The Massachusetts law of 1672 specified that “if any man smite out the Eye or Tooth of his Man-servant or Maid-servant . . . he shall let them go free from his service . . .”^109^ In 1718, Pennsylvania law stipulated that anyone who “put out an Eye . . . of any of the King’s Subjects” with malice and premeditation “shall suffer Death.”^110^ In 1742, South Carolina law stipulated that if one “put out the Eye . . . or deprive any Slave of any Limb,” the slaveholder must “forfeit the Sum of One Hundred Pounds.”^111^

Barham, the English settler in Jamaica, recorded the story of

captain Pickering . . . had a stick with fire at the end of it darted at him, which happened to come just under the brow of his eye, and seemed to turn his eye out . . . an old negro man . . . took of this herb that hath the bluish or purple flower, and washed it, reduced the eye as well as he could to its place, and then laid on the bruised herbe, bound it up . . .

The next day, a surgeon “found it healed up to admiration.”^28^ The herb was known as Prunella, Alheal, or Pickering’s herb.^24,28,62^

In 1744, physician Cadwallader Colden of New York wrote about witch hazel (*Hamamelis virginiana*):

I learned of the use of the *Hamamelis* from a Minister of the Church of England who officiates among the Mohawk Indians. He saw an almost total blindness occasioned by a blow cured by receiving the Warm Steam of a Decoction of the Bark of this Shrub through a Funnel upon this place. This was done by direction of a Mohawk Indian after other means had for a considerable time proved ineffectual. I have since experienced the benefit of it used in the same manner in an Inflammation of the eye from a blow.^1^

Many soldiers injured or lost an eye during the American wars of the 18th century, including the Seven Years’ War, known in America as the French and Indian War (1756-1763). Thomas Webb (1725-1796) of England served British General James Wolfe as a young lieutenant on July 31, 1759 at the Battle of Montmorency, near the city of Quebec.^112^ Webb saw a flash of light as a musket ball passed through one of his right orbital bones, “burst the eyeball,”^112^ and then passed through his palate, and was swallowed. Believing Webb was dead, his fellow soldier said, “He needs no help; he is dead enough.” Webb found the strength to reply “No, I am not dead.” After his recovery, Webb wore a green patch over his missing right eye. He became a Methodist clergyman and returned to England in 1778.^112^ When his remains were moved in 1972, his identity was confirmed by the green eye patch.^112^

During the American Revolution, officers known to lose an eye included Simeon Thayer and John Lamb. However, we have not found evidence that local surgeons were developing innovative treatments for eye trauma during this period. For instance, we are unaware of any American surgeons of this period suturing ruptured globes.

### Sympathetic ophthalmia

The understanding that injury to one eye can later result in inflammation in the other eye is said to date from antiquity.^113^ We see this understanding reflected in the writings of early New World physicians.

On the Madeira Islands (on his journey to Jamaica), Sloane, the English physician, examined a 35- or 40-year-old clergyman who had been shot with “small Shot, which lighted about his Temples.” The patient subsequently lost vision completely in one eye, which had “a Cataract (which I saw) grown in it. The other Eye (which is usual, when one is hurt) decaying so much that he could scarce see any thing with it.” Sloane understood that eye trauma could cause contralateral loss of vision. Sloane postulated that the shot “in all likelihood had weakened the Eye, made a small breach or (lying near it) Compression of the Optick Nerve.” Sloane treated by bleeding and with millipedes, and “white vitriol water, outwardly dropt on the Eye, to eat away the Films as much as might be.”^24^

In 1778, John Jay, an attorney (and, later, the first Chief Justice of the US Supreme Court), described an ophthalmic case of James Jay, his physician brother:

About five years ago [in 1773], a blind Frenchman, who had been maintained several years by the parish of Rye [New York], was brought to my brother. He had lost one eye fourteen, and the other five or six years. The sight of both eyes was equally opaque, and both equally useless. My brother chose to operate only on one at a time . . . He opened the one which had been blind fourteen years. The man recovered the sight of that eye, and requested the like operation on the other, but my brother declined it, on account of the connection, or sympathy, which he said subsisted between the two.^114^

The phrase “opened the eye” sounds like extraction, but was not typically used to specify extraction, and was often used to describe improving literal or figurative vision. As the attorney does not describe delivery of the lens, we suspect that this was a lay description of couching.

Hall Jackson of New Hampshire, who performed couching, wrote in a 1791 letter that he would not operate if either eye had vision, “but when wholly blind, nothing can be feared. The inflammation that sometimes happens after the operation in a diseased eye, might so effect the one not diseased as to deprive it of sight.”^58^

In May 1801, Peter Foissin, a student of “Physic” in Charleston wrote of a 25-year-old “negro man” who received a traumatic cataract in the right eye after “a blow with a whip.”^115^ The left eye “became affected with amaurosis, to the utter extinction of sight.” Foissin attributed the contralateral vision loss “Either from sympathy, effusion, or the excessive action of the rays of light.”^115^ Foissin wrote that the patient had an increase in “his venereal appetite” so that 3 women did not satisfy him.^115^ Also, the patient could sense objects near his body without touching them and had such a sharpening of his mental faculties that without sight he could now navigate the city and accomplish all his tasks well.

Thus, physicians in early America knew that trauma (or surgery) to one eye could cause contralateral vision loss, and from the cases of Jay and Foissin, we see that this process was sometimes described as “sympathy.”

### Artificial (prosthetic) eyes

A handful of practitioners advertised prosthetic eyes in the American colonies (Table 3).^116–120^ Naturally, those interested in beauty and aesthetics, such as surgeon James Graham, would be inclined to offer artificial eyes. The first advertisement after the Revolution came in 1790, in Charleston, from “Mons. De La Volatile . . . His artificial eyes are of a very beautiful assortment, brilliant black, languishing blue . . . with or without eyebrows.”^118^ De La Volatile also offered “bosoms of the most lovely constructions, with other inviting prominencies after nature, by which additions elderly ladies may pass for belles of five and twenty.”^118^

**Table 3.** Artificial (prosthetic) eyes.

Year	Oculist name and comment
1738	“. . . artificial Eyes . . . Supply’d . . . by Capt. Joseph Prince, at his House in Milk Street Boston”^116^
1772	James Graham: Philadelphia^117^
1790	“Mons. De La Volatile . . . His artificial eyes are of a very beautiful assortment, brilliant black, languishing blue. . . with or without eyebrows.”^118^ In Charleston
1791	Richard Cortlandt Skinner (d. 1834). “Dr. Skinner. . . Surgeon, Dentist, and Oculist,” claimed to be “the only operator in America that substitutes or sets Artificial Eyes.”^119^ He practiced in Philadelphia (1788), New York (1791-1795), Hartford (1795), Springfield (1806)
1792	Raymond Frederick: New York and Baltimore. “puts in artificial eyes resembling the natural, and which give no pain nor lay the patients under any constraint”^120^

Richard Cortlandt Skinner (d. 1834) trained in dentistry in London under Bartholomew Ruspini. After arriving in Philadelphia in 1788, Skinner asked Benjamin Franklin for a $20 loan to help get established.^121^ In 1791, he advertised in New York “Skinner, Surgeon-Dentist . . . Mr. Skinner substitutes artificial eyes in such a curious manner as to hide the deformity occasioned by the loss of an eye.”^122^ He charged 3 guineas to fit an artificial eye.^123^ In 1806, he wrote “Dr. Skinner . . . sets artificial Eyes . . . he will gratify curiosity by setting one in an orbit of his own Cranium or head” and claimed to have “the recommendations of several of the professors of the Columbia College.”^124^ Skinner’s work “*A Treatise on the Human Teeth*” is said to be the earliest American work on dentistry.^123,125^

### Albinism

A case of oculocutaneous albinism from America was presented in England: “In 1744, the child of a Negroe and a Negress about 5 years old, and born in Macondi in America, was brought before the academy.” The child

was perfectly white, with all the features of a black, all to his wooly hair and eye-brows, which were white: his eyes likewise constantly rolled in their sockets, and by exposing them to the light in a certain manner, the pupil appeared of a bright red, as did the choroides . . . he is weak-sighted . . . .^126^

### Cataract couching

In North Carolina, it was reported of alligators that “The Gall [bile] is of excellent use in taking away the Cataract and Web growing in the eyes.”^127^ The earliest advertisement for cataract treatment in the English colonies was the 1754 notice of Margaret Powell of New York, who “undertakes the Cure of all Rhumatic Pains . . . and Cataracts of the Eyes . . .”^128^ We suspect that the treatment was nonsurgical. This advertisement is consistent with the prominent role of women oculists noted in the 17th and early 18th centuries in England.^65^

In Mexico City, cataract couching was practiced as early as 1611 by Francisco Drago.^4^ We covered early cataract surgery in Latin America in a previous review.^4^ In the English-speaking colonies, surgeon John Morphy performed a couching on Montserrat in 1751 (Table 4).^3,87-89,129-133^ Because the operation caused the patient “anguish,” he ran through the streets, and the cataract rose again. When the cataract spontaneously fell back into the vitreous a year later, the patient could finally see.^3^

**Table 4.** Itinerant oculists and cataract surgeons.

Years	Name: comment
1751	John Morphy: Montserrat
1760–1764	William Stork, Jamaica: Annapolis, Philadelphia, New York, Boston
1770–1774	James Graham: Williamsburg, Annapolis, Baltimore, Philadelphia, New York.
1770–1781	Anthony Yeldall: Philadelphia, New York.
1771–1791	Frederick William Jericho: Extraction. Lesser Antilles, 1771–1776. Philadelphia, Baltimore, Richmond, Norfolk, Charleston, Boston, New York, 1783–1785. Jamaica 1785–1791
1772–1776	Stephen Little of Portsmouth New Hampshire. Couching in New York in 1772
1773–1777	Francis Mercier (Louis DeBuke): Baltimore, Philadelphia, New York, Boston, 1773–1777
1774–1783	“Mr. Dastugue Physician and Surgeon, lately arrived from France . . . propose[s] that the Physicians and Surgeons in this Town . . . form a Corps to build an Amphitheatre . . . to teach Osteology, Truss, Physiology . . .” in Boston 1774–1775.^87^ “Richard Durfort Dusturge. Surgeon from Europe . . . Occulist.”^129^ Mentioned practice as an oculist only in 1777 in New York.^87^ Studied at Charity Hospital and Hotel Dieu in Paris^130^
1775–1784	“Dr. Ludwig . . . from Germany, practices medicine and surgery, dentist and oculist.” Treats “eye trouble” Philadelphia (1775),^87^ Yorktown (1776–1783), Baltimore (1783–4).^131^ No mention of the eye after 1775
1778	Friedrich Carl Pflug. Philadelphia and New York.^87,88^ “Surgeon of the Hessian Grenadiers Battalion . . . operation for the cataract”^87^
1779	“Deui Cheau” (possibly mistranscribed) in Baltimore, Philadelphia 1779.^132^ “A French doctor and surgeon.” “Oculist, is skilled in Midwifery, . . . He also has had great success at Baltimore”^133^
1790–1798	George Kiesselbach: Extraction (probable). Charleston, Norfolk, Boston, Newport, New York, Philadelphia (1790–1798)
1791	Peter Degravers, Jamaica^89^: Extraction
1792–1795	Bildad Beech, Whitestown in New York and Cheshire, Connecticut
1793–1799	Jean Devèze, of France. In Philadelphia. Had come from Santo Domingo. Operated for cataract in 1795
1801–1806	Charles F Bartlett. Extraction. Newport, New York, Hartford. Darien

During the later half of the 18th century, we found 40 surgeons known to have performed or advertised cataract surgery and additional oculists or surgeons who might have performed the procedure (Tables 4 to 8).^129–180^ Additional evidence of cataract surgery in America comes from advertisements for couching instruments after 1763 (Tables 5 to 7) and aphakic spectacles after 1793 (Table 6).

**Table 5.** Cataract surgery and oculists in Philadelphia.

Year	Surgeon: Comment
1763–1792	Couching instruments: “Smith and Harris . . . Surgeons Instruments . . . Eye probes” 1763,^134^ Nathaniel Tweedy, 1764, 1766, 1768.^135–137^ John Sparhawk, 1772–1773, 1781.^138–140^ Pennsylvania Packet printer. “couching instruments,” 1780.^141^ “William Smith . . . instruments . . . couching,” 1782–1783.^142–143^ Oliver C. Hull, 1792.^144^ Goldthwait and Baldwin, 1792^145^
1768	John Flemor: “. . . knowledge . . . acquired in some of the most eminent hospitals in Europe . . . cures most disorders in the eyes, particularly the cataract, by a new operation of couching”^146^
	Charles Henry Forget: “Doctor Forget . . . Skill as an Oculist”^147–148^
1774	“Doctor Adams, Oculist . . . pearls, films, rheums, or dull sight, without operation”^149^
1779–1808	William Shippen, Jr. acquired couching instruments, 1779^150^
1784–1796	John Foulke, MD (1757–1796): Had cataract instruments in his estate^151^
1785–1794	Joseph Goss: Advertised cataract surgery in 1785^152^
1789–1818	Caspar Wistar couched Humphrey Marshall in 1793
1793–1794	“Andrew Girinzer, German Physician and Surgeon . . . diseases incidental to the eyes . . . practiced . . . in Paris”^153^ Served Emperor Joseph in the “war against the Turks”^153^
1795–1837	Philip Syng Physick: Extraction

**Table 6.** Cataract surgery and oculists in New York and New Jersey.

Years	Surgeon: region
1754	Margaret Powell: Treated cataract (likely nonsurgical)^128^
1762–1773	John Levine: “Oculist . . . Cures all Disorders in the Eyes.” 1762-1767.^154–156^ From Ireland. Had lived in England^157^
1768	Couching instruments: William Shipman, 1768.^158^ Joel and Jotham Post. “Surgeons’ instruments for . . . couching.” 1794^159^
1773	James Jay: Rye, New York
1783	Richard Bayley: Extraction
1784	Charles McKnight (1750-1791): “Died . . . as a surgeon and oculist, perhaps unequalled in this country.”^160^ (Date began eye surgeries estimated)
1788	Charles Crooke (1764–1788): Poughkeepsie.^161^ Estate sale: “Surgeon’s Instruments . . . Couching”^162^
1792	“. . . an operation, . . . performed by Doctor William Stillwell, surgeon, . . . upon an eye, that had been blind for three years past . . . he now sees the distance of 20 or 30 rods . . .”^163^ Middletown, New Jersey
1793	Aphakic spectacles. “James Rivington has . . . for the accommodation of persons, with couched & weak eyes . . . spectacles.”^164^ 1793-1794.^165^ Joel Benson. . . Optician. . . spectacles for cataract eyes. 1798^166^

All practitioners in New York City unless another location specified.

**Table 7.** Cataract surgery in New England.

Year	Surgeon: regions
1767	James Thomson: Hartford. Cases of hypopyon and mydriasis.^167^
1769	John Bartlett: Charlestown, Rhode Island^168^
1770	Hall Jackson: Portsmouth, New Hampshire
1773	Benjamin Church^169^
1774	Couching instruments. In Boston. Nathaniel Kidder (1774).^170^ Thomas Bartlett (1797)^171^
1782	Lewis Leprilete. Extraction. Providence, Norton, Franklin
1788	John Warren, in Cambridge
1791	Gustavus Baylies in Newport, Bristol 1791 to 1803.^172–173^ “Couching the Cataract . . .”^174^
1792	Nathaniel Miller: In Franklin. Extraction
1793	Eldad Lewis: Lenox Massachusetts
1798	Nathan Smith: Cornish
1801	Mason Fitch Cogswell: Hartford
	Horace Senter: Extraction. Newport

**Table 8.** Cataract surgeons and oculists in the South.

Year	Surgeon: regions
1768	Dr De Lacoudre: Norfolk. He was trained in France by Drs Guerin and Morant. He has a certificate from King George III. If you cannot travel to see him, you can send your urine, and he will analyze it, and return relevant medical information^175–176^
1779	John Allard: Charleston. “Expert in Surgery Dentist, Oculist . . .”^87,177^
1785	William Baynham (1749-1814): Essex, Virginia
1786	John Tyler (1763-1841): Frederick, Maryland
1793	Gabriel N Phillips: Edenton, North Carolina. Trained in New York^178^
1797	Nathan Brownson died in Riceboro, Georgia. His estate had couching instruments^179^
1798	Joseph Brevitt (1769-1839): Extraction. Baltimore
1799	“R. C. Murray (Occulist). . . performs the different operations on the eye, has been successful in restoring a number of persons to sight that had been blind many years’’^180^: Baltimore. Died 1801

The method of couching was described in the lecture notes of John Warren’s student at Harvard University in 1794:

Couching . . . by passing the instruments into the sclerotica about 1/6 part of an inch behind the cornea, and then looking for the point of the needle till it is observed immediately above the lens. The needle is then to be turned on its flat side downwards, and the lens to be depressed till it does not rise. The instrument then is to be withdrawn in the same manner as it was introduced—The wound then to be dressed superficially.^105^

Placing the couching needle 1/6 in (4.2 mm) posterior to the limbus results in pars plana entry.^181^ As the needle is observed just anterior to the lens, rather than the iris, the needle passed posterior to the iris. Thus, the general approach to couching had not changed since described by Celsus in the first century.^181^

**Figure 6.**
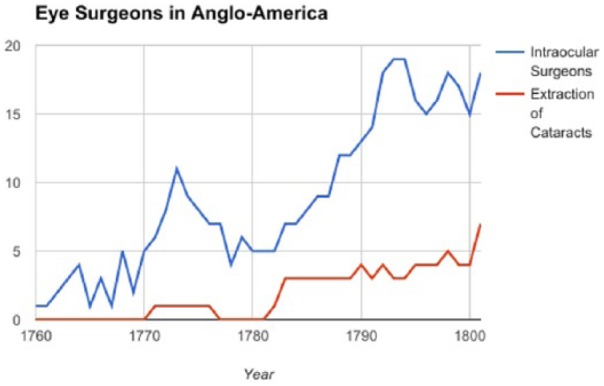
Number of identified cataract surgeons, total (couching plus extraction, top line), and those who preferred extraction (bottom line), by year. Each shopkeeper who advertised couching instruments in a given year was counted as one surgeon, with the assumption that the shopkeeper likely sold at least one set of instruments to a surgeon who did not advertise.

The American Revolution from 1776 to 1783 was associated with a reduction in the number of eye surgeons and oculists in the colonies (Figure 6). Even at the turn of the 19th century, only about one-third of the surgeons performed cataract extraction, as opposed to couching (Figure 6).

### Cataract extraction

Cataract extraction, especially of soft cataracts, had been occasionally performed since antiquity.^182^ In the early 1700s, John Thomas Woolhouse, Charles de Saint-Yves, and others, extracted lenses which accidentally subluxed into the anterior chamber during couching.^183^ But the popularization of planned cataract extraction in the modern era began with the presentation of Jacques Daviel in Paris in 1752.^3^

American physicians first learned of cataract extraction from books. In July 1766, the New Jersey Medical Society established a fee schedule (adopted formally in 1784) which set the price for “Couching or extracting the cataract [£3] . . . Cutting the Iris [£3] . . . Fistula Lachrymalis [£1, 10s].”^184^ The language of the entire fee schedule, including the order of the operations and the spelling, was taken from the 1761 surgical text of Samuel Sharp, from Guy’s Hospital in London.^106^ We doubt that American surgeons were actually performing cataract extraction or iridectomy in 1766.

**Figure 7.**
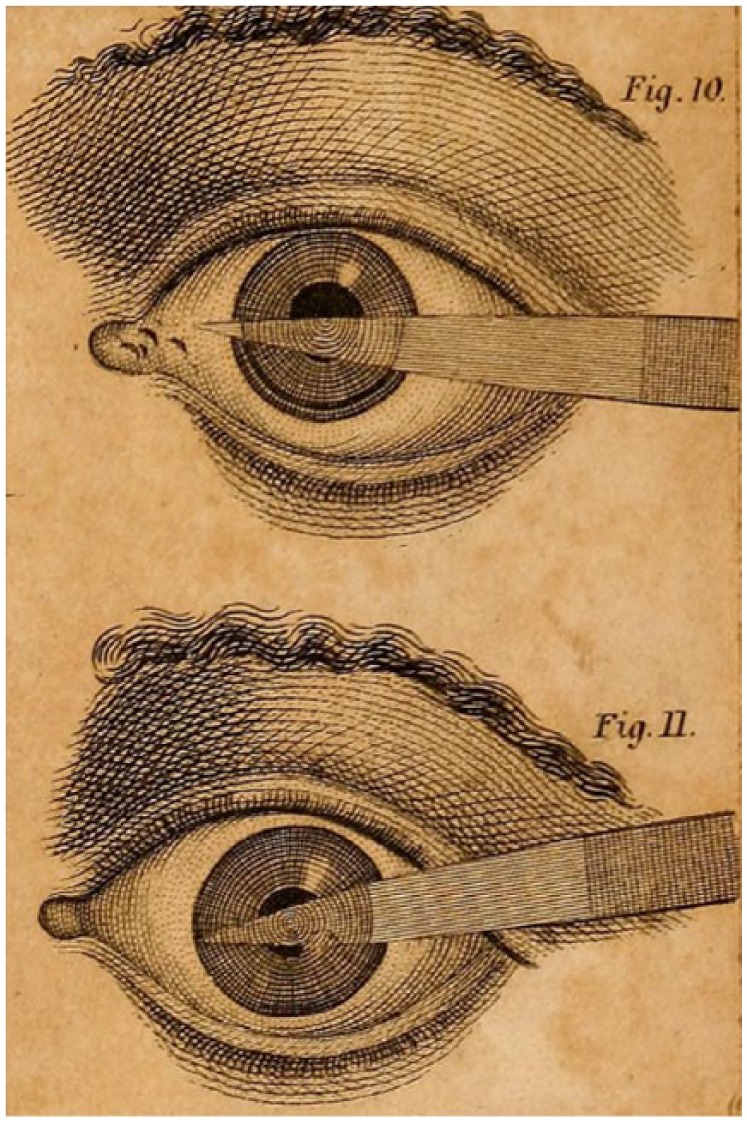
Cataract extraction, typical (upper plate), and with iris protruding anterior to the knife (lower plate), in the works of John Syng Dorsey.^81^

Ten surgeons who performed cataract extraction were identified by 1801 (Tables 4 to 8, Figure 7).^81^ The earliest identified surgeon to perform cataract extraction in the New World was Frederick William Jericho of Germany, who trained in Holland.^3^ Based on the record in Europe, it appears that his initial cataract surgeries in the Caribbean must have been between 1771 and 1776. On his return to England in 1776, Jericho provided recommendations from Antigua, St. Kitts, and Montserrat^86,107^ and noted,

He also performed the operation of the Cataract on a negro girl; who immediately after could distinguish objects clearly, and in a fortnight’s time able to go abroad and about her business . . . He also cured a Mulatto girl, who had long lost the use of both her hands, and was totally deprived of eye-sight, to both of which she was restored, and is now able to do the finest needlework. He has restored several in the Island of Antigua to their Sight by the operation of the cataract . . . .^107^

The incision pioneered by Jacques Daviel for cataract extraction involved a small inferior incision with a knife, followed by extension of the incision with right and left curved scissors. Others made the incision with a single knife which made entry and exit corneal incisions near the limbus, which were then connected by moving the knife. In the Americas, this type of incision was used by Jericho,^3^ Degravers,^95^ and John Warren at Harvard. In 1794, Warren’s student recorded the method of cataract extraction:

As by passing a sharp edged instrument into the cornea about 1/12 of an inch from its edge and out at the opposite side. The edge of the instrument is then passed obliquely downwards & outwards cutting thro’ the cornea which is left suspended like a flap—the lens is the pressed out thro’ the pupil.^105^

As Warren had no technique to evacuate the lens other than simple pressure, it is not surprising that he would have had poor outcomes with extraction and would have favored couching. Warren’s student recorded,

The former of these [i.e. couching, as opposed to extraction] is to be preferred on various accounts—The violence done to the pupil in forcing out the lens often injures it irreparably—and the cicatrix made by the latter is much greater and more injurious than that occasioned by the former. In both cases the dressings should be wet with aqua vegeto mineralis [lead subacetate solution] & the light excluded.^105^

Jericho noted that after the incision, muscular contraction might spontaneously cause lens expulsion.^3^ But if the lens was not expelled, Jericho had strategies beyond simple application of pressure. He elevated the cornea and performed a form of capsulorhexis:

. . . the incised part of the cornea should be raised up by a surgeon’s needle, and when brought forth through the aperture and pupil, the capsule in which the lens is enclosed should be scraped or perforated; although it is much better to use a needle or a thin and sharp instrument ending in a point, which at its end is blunt on either side.^185^

If the lens breaks up, it is removed with a spoon, and posterior synechiae are carefully dissected:

Sometimes it happens that the lens leaves the eye in pieces . . . the surgeon will try to remove the remaining part of the lens from the eye with the thinnest of spoons, which does not always succeed, especially if the lens has coalesced in some part with the posterior surface of the uvea; when this happens the fragments should be carefully freed from their binds by seizing them with a small set of forceps and using the thinnest pair of scissors.^185^

Similar to Jericho, Degravers performed a form of capsulorhexis: using a “kystitome,” the operator “incises inferiorly, transversally, and at one cut, the capsule of the crystalline.”^95^

Philip Syng Physick, at the Pennsylvania Hospital from 1794 onward, performed extraction by placing the knife “1/12 of an inch within the sclerotica . . . and bring it out upon the opposite [side] of the cornea.”^98^ Then, he incised the anterior capsule: “. . . the needle is introduced and the capsule torn as much as possible” before expulsion of the lens.^98^ Physick’s teachings on eye surgery were recorded in Elements of Surgery, written by his nephew, John Syng Dorsey, published in 1813.^98^

### Datura stramonium

*Datura* species native to Mexico fall within the family of nightshade plants which dilate the pupil. The mydriatic properties of the European counterpart, *Atropa belladonna*, had been known for centuries, but belladonna extracts were not widely used in European ophthalmology before the 19th century. Aztec healers used Datura species for a number of medical conditions and in divination rituals because in high doses they induce hallucinations.^186^ In 1676, native *Datura stramonium*, “which resembles the Thorny Apple of Peru” was consumed in a “boil’d Salad” by British soldiers who were attempting to suppress Bacon’s rebellion in Virginia.^187^ The soldiers were incapacitated for 11 days. The anecdote gave the plant its colloquial name “James-Town Weed”^187^ or jimsonweed.

In April 1801, Samuel Brown, a doctor in Lexington, Kentucky, treated a 2-year-old girl who behaved strangely, fell to the floor as if paralyzed, and developed fever, delirium, and “scarlet efflorescence over the whole body.”^188^ She alternated between weeping, screaming, laughter, and incoherence. The pupils were dilated, with “a remarkable squinting of the right eye, which, however, was not constant.”^188^ This observation probably reflects an intermittent esotropia, as partial cycloplegia induced excessive efforts at accommodation and therefore convergence. The doctor induced vomiting, which produced seeds of “the stramonium.” Over the next few days, he prescribed “rhubarb and pot-ash.” The child recovered. Brown proposed that poisoning with stramonium was probably common. He also asked “Would not the topical application of the stramonium, by its power of dilating the pupil, facilitate the extraction of the cataract?”^188^ Dilation with belladonna was just being introduced in Europe. It appears that this frontier American doctor thought of the idea independently.

### Surgical charges

In the available fee schedules, we see no price differential between cataract couching and extraction, even though the latter required more skill and training (Table 9).^189–191^ Several surgeons offered a reduced fee when the surgery failed.

**Table 9.** Surgical charges.

Year	Surgeon(s)	Surgery	Price
1766	New Jersey Medical Society^189^	“Couching or extracting the cataract”	£3
		“Fistula Lachrymalis”	£1, 10s
		Cutting the Iris	£3
1799	William Baynham: Essex, Virginia^92^	Unsuccessful surgery for “tumor in the left Eye . . . film in the right”	$5
1800	Nathan Smith: Cornish	“extracting a tumor near an eye”	$5
		“extracting an eye”	$22
1801	Charles F Bartlett: Newport	“for extracting or couching the cataract”^190^	$50
	Mason Fitch Cogswell: Hartford	Unsuccessful operation for cataract. Probably couching	$20
1804	Medical Society (Charleston)^191^	“The Operation for the Cataract”	$10
		“Fistula Lachrymalis”	$10

## Surgeon Biographies and University Ophthalmology

In this portion of the article, we present the stories of particular surgeons and universities in early North America. The aim is not to present every surgeon’s biography exhaustively. Rather, we present the materials not previously highlighted or reported to our knowledge. Also, the early development of ophthalmology at academic institutions is presented.

### Early American physicians, from 1687

**Figure 8.**
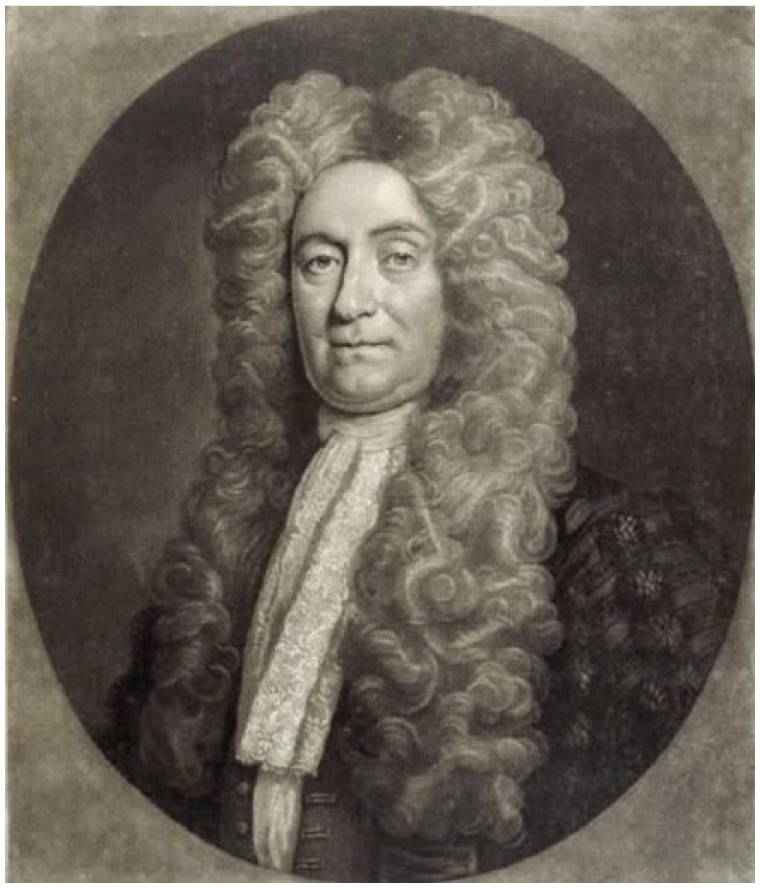
Sir Hans Sloane (1660-1753), an engraving from a portrait by Murray.^192^

Hans Sloane (1660-1753) of England practiced as a young physician on Jamaica in 1687 (Figure 8).^192^ Sloane merged his own medical and scientific observations with those of previous naturalists, such as Francisco Hernández de Toledo. Although Jamaica was claimed by Spain after its exploration by Columbus in 1494, the British captured the island in 1655. Sloane reported that the Spanish abandoned the northern side of the island because in this region “Ants are said to have killed the Spanish Children by eating their Eyes when they were left in their Cradles . . .”^24^

Sloane recounted the histories of many ophthalmic patients on Jamaica.^24^ He had been told that the diseases and treatments would be substantially different in the New World. However, he soon found that his European medicines and cures were quite effective. Moreover, the patients had faith in his methods, complied well with treatment, and refrained from “judging harshly in case the Person died.”^24^

Trader James Adair lived with the Natchez, the Chickasaw, and other native tribes between 1735 and 1750. Adair wrote of one interaction:

Formerly, an old Nachee [Natchez] warrior who was blind of one eye, and very dim-sighted in the other, having heard of the surprising skill of the European oculists, fancied I could cure him . . . I had just drank a glass of rum when he came to undergo the operation. . . he observing my glass, said, it was best to defer it till the next day.—I told him I drank so on purpose.^193^

Adair also gave the patient “several drinks of grogg.”^193^ Then, Adair

applied my materia medica, blowing a quill full of fine burnt alum and Roman vitriol into his eye. Just as I was ready to repeat it, he bounded up out of his seemingly dead state, jumped about, and said, my songs and physic were not good.^193^

### Benjamin Franklin and the Pennsylvania Hospital in 1751

As with so much of early American life, ophthalmology was influenced by the leadership of Benjamin Franklin. Early settlers had to receive spectacles imported from England. Franklin’s Pennsylvania Gazette carried advertisements for these spectacles. Among the earliest to advertise spectacles was Franklin himself in 1738.^194^

Avoidance of indoor smoke was one of the advantages claimed by Franklin for his stove or “Pennsylvanian Fire-Places.”^195^ Franklin noted, “Great and bright Fires do also very much contribute to damage the Eyes . . .”^195^ He also noted, “This Fire-place cures most smoaky Chimneys, and thereby preserves both the Eyes and Furniture.”^195^

**Figure 9.**
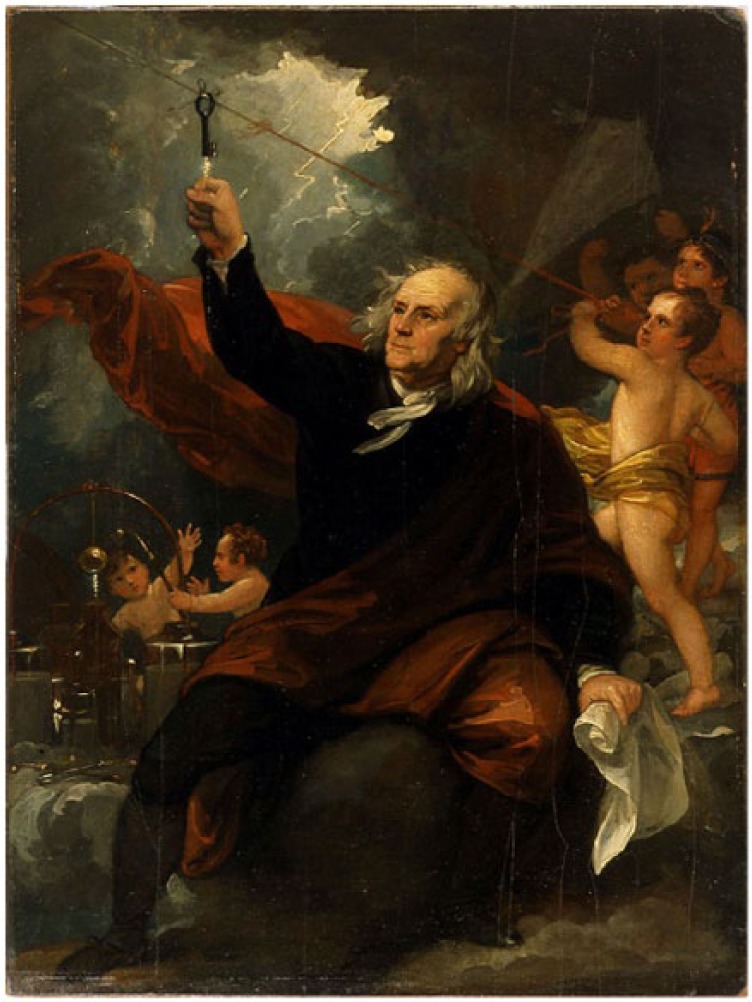
Benjamin Franklin drawing electricity from the sky (by Benjamin West, circa 1816).^196^

One of Franklin’s greatest accomplishments was proving that lightning was electricity (Figure 9).^196^ This realization allowed him to invent the lightning rod. Franklin noted, “Before I leave this Subject of Lightning, I may mention some other Similarities between the Effects of that and those of Electricity. Lightning has often been known to strike People blind.”^197^ Indeed, lightning strike can produce vision loss from cataract, optic neuropathy, or central nervous system injury.^198^ Franklin continued by describing his experiments affecting several animals “by the Electrical Shock.” He noted, “a Pullet [hen] struck dead in like Manner, being recover’d by repeated blowing into it’s Lungs . . . on Examination appear’d perfectly blind.”^197^ It seems that Franklin performed successful mouth-to-mouth resuscitation on a hen.

In 1752, Benjamin Franklin argued to physician Cadwallader Colden that light was a wave, rather than a particle, that proceeds to the eye:

May not all the Phaenomena of Light be more conveniently solved, by supposing Universal Space filled with a subtle elastic Fluid, which when at rest is not visible, but whose Vibrations affect that fine Sense the Eye . . . ? . . . why must we believe that luminous Particles leave the Sun and proceed to the Eye?^199^

Colden replied that as pointed out in “Sir Isaac Newton’s optics,” a particle theory could better explain how an opaque object obstructs the passage of light to the eye.^200^

**Figure 10.**
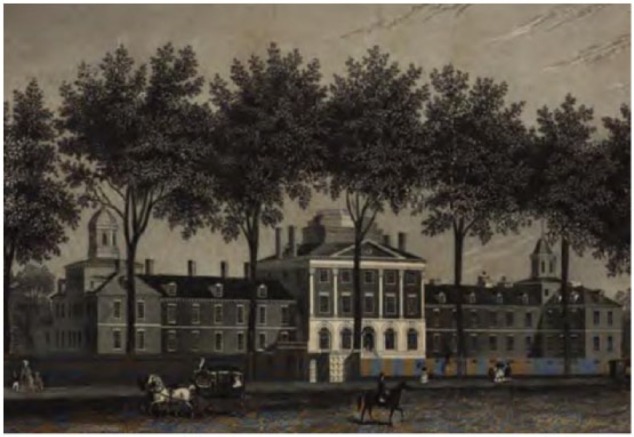
The Pennsylvania Hospital.^201^

A petition presented to the Assembly of Pennsylvania in January 1750-1751 called for the establishment of a hospital, which led to the Pennsylvania Hospital.^189^ The petition was written by Franklin, although he was not a signatory.^189^ The petition noted the need to help those “deprived of Sight by Cataracts.”^189^ The result of these efforts was the establishment of the Pennsylvania Hospital, the first in the English colonies, in 1751 (Figure 10).^201^ Franklin and physician Thomas Bond (1712-1784) are considered the hospital founders.^201^ As the hospital reports from the 1750s mention “Eyes disordered,” “Gutta serena,” and ophthalmia, but not cataract,^108^ it is not clear that the hospital was initially able to deliver on the promise made to the legislature.

In 1749, Franklin had helped to found the Academy of Philadelphia, later known as the College of Philadelphia, a predecessor of the University of Pennsylvania. This institution began offering medical training in the 1760s. The first professor of surgery was William Shippen, Jr (1736-1808, Figure 11).^202^ Shippen had first studied with his physician father and then traveled to Europe for additional training. While in London, the younger Shippen recorded in his diary on August 25, 1759 “. . . saw Mr. Way and Paul couch 2 men in old way by depression.”^203^ Lewis Way was the surgeon to Guy’s Hospital, and Joseph Paul was the surgeon to St. Thomas’ Hospital.^203^ Shippen watched John Hunter “extract a Steatomatous Tumor from [the] upper eyelid.”^203^ Shippen also read Percival Pott’s treatise on the Fistula Lachrymalis.^203^ On his return to Philadelphia, Shippen began offering private medical lectures in 1762. Notes from a student of Shippen’s 1766 lectures do not relate to anatomy or surgery of the eye, but the notes might be incomplete.^204^

**Figure 11.**
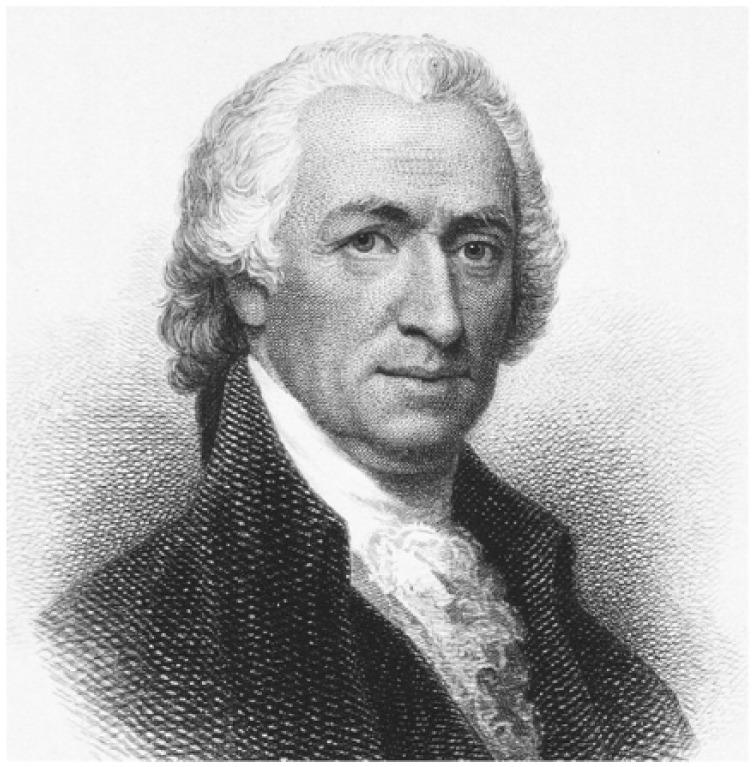
William Shippen, Jr (1736-1808) of Philadelphia.^202^

“Doctor [William] Shippen” was listed as the “Professor of Anatomy and Surgery” on the notice of the first medical graduation ceremonies of the College of Philadelphia on June 21, 1768.^205^ The notice began “This day . . . may be considered as having given birth to Medical Honours in America.”^205^ Ten students who had attended lectures at the Pennsylvania Hospital were awarded the “degree of Bachelor of Physick.”^205^ The ceremonies featured “A dispute, whether the Retina, or Tunica Choroides be the immediate seat of Vision.” One student correctly argued that the retina received the image, but another argued

that the Retina is incapable of the office ascribed to it, on account of its being easily permeable by the rays of light: and that the Choroid-coat, by its being opake, is the proper part for stopping the rays and receiving the picture of the object.^205^

In 1777, Shippen was appointed the Director of Hospitals for the Continental Army. In 1779, he ordered equipment including “1 case of couching instruments” from the apothecary general, Dr Andrew Craigee.^150^ A dispute ensued about whether these were for the use of the Army or him personally.^150^ Shippen was court-martialed for mismanagement but was narrowly acquitted.

Franklin spent much of the prerevolutionary period in England on political assignments. At the end of 1776, he moved to Paris as the American ambassador to France. He mentioned his invention of “double spectacles” (bifocals) while in Paris in 1784^206^ and returned to America the next year.

### Oculists from Europe (1761-1783)

In the colonial period, trained oculists migrated from Europe to the American colonies. One early cataract coucher was William Stork, who was the oculist to Augusta, the Princess of Wales from 1751 to 1754.^3,207,208^ After practicing in Yorkshire for several years,^209^ Stork advertised his services as an oculist in Kingston, Jamaica in 1760.^3,210^ In his later editing of Bartram’s botanical guide appended to his own journal, Stork cited Sloane’s work on Jamaica.^211^ Thus, Stork might have been inspired to follow Sloane’s example as an oculist on that island and would have been familiar with the ocular ailments and treatments described by Sloane. Stork arrived in Philadelphia in 1761 and then practiced in cities between Annapolis and Boston until 1764.^3,212^ Stork then promoted the settlement of Florida.^3^ The William Stork of Florida was indeed the “oculist.”^213,214^ Stork died there in 1768.^3,213^

**Figure 12.**
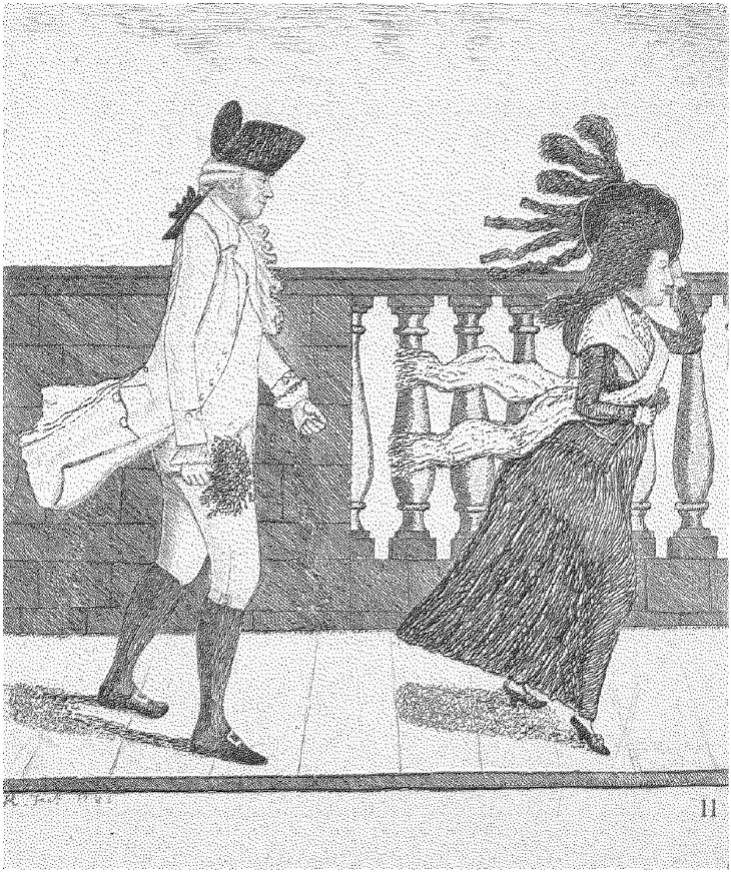
Dr James Graham (1745-1794) going along the North Bridge in a High Wind, by John Kay in 1785.^215^

James Graham (1745-1794) was another prominent European eye surgeon in colonial America (Figure 12).^215^ Graham matriculated at the University of Edinburgh in 1761,^216^ where anatomy and surgery were taught by Alexander Monro, junior and senior.^216^ By the time Graham married in 1764, he had established an apothecary shop in Doncaster, England.^216^ Later in the 1760s, Graham spent time in the hospitals of Dublin and London.^216^

Graham traveled to America, arriving at the end of the summer of 1769.^216^ In January 1770, he advertised a “Lecture on the Eye” in Annapolis.^216^ By August 1770, he was advertising treatment of disorders of the eyes and ears and of “female complaints” in New York.^216,217^

In May 1771, he “cured” 173 patients with “blindness, deafness, and female complaints” in Elizabethtown, New Jersey.^218^ It may seem unusual that a professed oculist and aurist would also attend to female complaints, but, as we will see, on his eventual return to England, Graham specialized in helping couples attain sexual pleasure and fertility. Hints of his subsequent career path can be found during his North American stay.

At the end of the summer of 1771, a theatrical competitor named Anthony Yeldall was attracting a great deal of attention in New York (see below). Perhaps for that reason, Graham relocated to Philadelphia in the fall^219^ and published testimonials of patients successfully treated for “fistula lachrymalis,” “gutta serena,” and “a film over his right eye” from smallpox.^83^ In April 1772, Graham published his first testimonial from a patient he couched, Mrs Mary Rivel, who had been “blind of both eyes for two years.”^220,221^ He next published the testimonial of a man “blind of both eyes with a confirmed Gutta Serena” who was now able to read.^222^ Given his interest in beauty, it is unsurprising he would fit prosthetic eyes:

Those persons whose eyes are utterly perished, or sunk in their head, may have the deformity removed by artificial eyes, so curiously fixed and adapted to the orbits, as to have in appearance the beauties, motion, &c. of the natural eye in its healthy state.^117^

After Graham’s departure, prosthetic eyes were not advertised again until 1790 (Table 3).

Graham’s next advertisement noted that “. . . seven [of his patients] had lost their Sight by Beards of Rye, and other Grain, getting into their Eyes in Time of Harvest, of these, three only have perfectly recovered.”^223^ The report also mentioned 2 patients he couched: “. . . a Gentleman advanced in Years was couched in one Eye, but the Operation proved ineffectual.”^223^ He also couched the “Son of Mr. Thomas Walling . . . aged Eleven, and who, for upwards of three Years, had been totally blind of both Eyes, was instantly restored to the sight of both.”^223^

At the end of the summer of 1772, Graham announced that he was going to return to England in 1773, but that for a fee, he would instruct one student “regularly bred to the profession of physic or surgery” as an oculist and aurist.^224^ He treated “. . . squinting . . . blows or extraneous substances. . . tumours and excressences. . .”^224^ Graham announced visits to Lancaster, York, and Reading.^225^

He also announced a lecture on the eye at “the Doctor’s apartments” and invited the public as well as “the faculty” to attend.^225^ The lecture enjoyed some success because he was able to present it at “the College Hall” of the College of Philadelphia, however, it is not clear that the college endorsed or sponsored his talk.^226^ The lecture had 3 parts with “A Concert of Musick” during the intermissions.^226^ The first part consisted of anatomy, physiology, the pathway of light through the “Coats and humours of the Eye,” with “Refraction and reflection of the rays.”^226^ Graham addressed the “students in medicine and surgery” noting the “College of Philadelphia in the general field of literature, considered as equaling, and in the liberal profession of medicine as far surpassing, any other on the British continent of America.”^226^ The second part described the causes and cure of diseases such as “Cataract—Glaucoma—Gutta Serena—Inflammations . . . Films. . . Squinting . . . Short-sightedness—useful directions concerning spectacles . . .”^226^ Consistent with his ultimate career pathway, Graham provided an “Address to the ladies on the art of managing the eye . . . Beauty of a fine woman composed of numberless lesser beauties, of which the Eye is the chief.”^226^ This section seems to be a course on flirtatious glances and putting makeup on the eye. The third part of Graham’s lecture was about the “dignity and importance of physic and surgery” and noted “. . . the pleasures of the eye as being more refined or spiritual” than those of the other senses.^226^

In the Spring of 1773, he took a quick trip to Annapolis and Williamsburg.^227^ In Williamsburg, on May 5, 1773, a young legislator named Thomas Jefferson purchased “a ticket to Graham’s lecture on the eye.”^228^ Jefferson’s interest was probably intellectual rather than personal. He seemed to suffer little in the way of ophthalmic complaints. He did write in 1763 that “. . . the loss of the whites of my eyes, in the room of which I have got reds, which give me such exquisite pain that I have not attempted to read any thing . . .” and continued “[My] eyes still continue [as red a]s ever, and if they were to begin to mend now . . .”^229^ Not until 1789, did he need to purchase a “set of reading glasses.”^230^ In 1798, Jefferson wrote about “. . . a small cold which brought on an inflammation in the eyes . . .”^231^

The newspapers recorded that

. . . Mrs. Cobb of Williamsburg, aged sixty-six, who for several years had been totally blind with a Cataract in each Eye, was couched by Dr. Graham . . . and in less than five minutes was restored to the blessing of sight in both Eyes.^232^

The report called “Mrs. Cobb . . . the first patient on whom the Doctor has operated in a Cataract.”^232^ In fact, Mrs Cobb was at least the fourth, if the newspapers were to be believed, but perhaps the message is that Graham had not performed many couchings. In addition, “The first patient with a Gutta Serena (a diease hitherto deemed incurable) was Miss Peggy Hay . . . She too was happily restored . . .”^232^

Graham then traveled to Philadelphia and then New York.^227,232^ Here, he delivered his lecture on the eye, again inviting “the Faculty.”^233^ Graham perhaps was tiring of surgical practice, for he published the testimonial of Martha Cooke, of New York, whose cataracts he cured by “inward medicine and outward applications,” done “without cutting or any painful operation.”^234^ He arrived in Baltimore in October 1773.^234^ In December 1773, 4 days after the Boston Tea Party, he stopped off in Philadelphia before returning to England.^235^ Graham later wrote “I returned to England at the commencement of the eternal downfall of European power in America!”^216^

On his return to England, Graham initially resumed practice as an oculist and aurist and republished his testimonials from America in 1775.^236^ Strangely, in the republication of his August 1772 advertisement, Graham claimed that the “Gentleman . . . had the cataract extracted” (rather than couched).^236^ Graham still noted the lack of effect.^236^ It seems that in the retelling Graham lied about the procedure performed, perhaps to appear current with procedures in vogue in Europe. Still, he told the truth about the poor outcome, perhaps because in his return to nonsurgical remedies, he did not want to claim great benefits for any kind of surgery. Perhaps, due to a tighter regulatory environment in England, or a waning interest on his part, it seems that Graham performed no cataract surgery either before or after his stay in America.

In 1776, Graham publicized a budding interest in the curative powers of electricity and magnets.^237^ He claimed that an interest in studying electricity in Philadelphia had motivated his travels to America.^237^ In reality, he spent over a year in Maryland and New York before practicing in Philadelphia. Graham also claimed that he studied electricity in Philadelphia and had benefited from the work of “the great prince of philosophers” (Franklin), who actually was in Europe at that time.^238^ Graham wrote that in America he had constructed an “electrical bed” which enhanced sexual pleasure and which permitted a woman from Lancaster with reproductive issues to become pregnant.^238^ His biographer accepted this case report at face value,^216^ but there is reason to be skeptical. As Graham did not publicize any interest in electricity or magnets prior to 1776, there is no way to know if he was merely trying to appear to have more experience in this area and associate his electrical work with Franklin.

Graham eventually combined his long-standing hobby of reproductive medicine with his newfound interest in electromagnetism and transitioned to specializing in the treatment of sexual dysfunction. By 1780, he had constructed his “magnetico-electrical bed,—the first and only one that now is or ever was in the world.”^239^ In this “Celestial Bed,” couples could “Be fruitful, multiply, and replenish the earth.”^216^

Anthony Yeldall was another early oculist in America. He learned medicine from the lectures and practices of “eminent men” from Europe and from traveling through England, Scotland, and Ireland for 12 years.^85^ On his 1770 arrival in Philadelphia, he advertised as a surgeon who “couches cataracts in the eyes,” “cuts for the stone,” performs “amputation” of limbs or breast cancers, and “cures hair lips” (cleft lip).^240^ He also treated hernias (“ruptures”), dropsy, gout, and syphilis (“French disease”).^240^ He proposed rotating visits to Pennsylvania and Delaware towns near Philadelphia.^240^

Later in 1770, he began to emphasize a business selling “Medicines.”^241^ In 1771, this business was marketed with an itinerant theater production, in which Yeldall “sells medicines from a stage” and the people were entertained by “the odd tricks of his Merry-Andrew” (apparently an acrobat).^242^ At Brooklyn, “several thousand” people witnessed his exhibition daily.^242^ Near the end of the summer of 1771, a boat with 110 people returning from the exhibition began to sink, producing “tears and cries of the women and children, the looks of astonishment and terror of the whole company, at the prospect of immediate death.”^242^ Fortunately, the people were rescued by other boaters. Of course, a theater production hardly seems the most ethical manner for a surgeon to advertise. As far as we know, the businesses of the traveling druggist and the surgeon were separate. We have not seen evidence that Yeldall offered surgical services from the stage.

In early 1772, he returned to Philadelphia.^243^ In the fall of 1773, in Connecticut, Yeldall and his acrobat and apprentice were arrested “as strollers and idlers.”^244^ Only the acrobat was convicted.^244^ Also, while the doctor was on stage “expatiating on the virtue of his medicines,” the parish parson began to harangue Yeldall.^244^ Yeldall debated the parson, with “victory declared in favour of the Doctor.”^244^ After these episodes, we have no evidence of Yeldall trying to revive his theater marketing schemes.

He returned to Philadelphia, now abandoned by Graham, and revived the druggist business there.^245^ He also advertised cataract couching “giving sight to the blind in one minute’s time” and cleaning films from the eye.^85^ He could send to other doctors “An Eye Powder . . . for taking off specks, films, webs, &c. (if not mixt with the horny or outward coat) of the eye, curing watery, bloodshot, or inflamed eyes.”^85^ It is not clear if his own surgical practice merely involved placing the powder or if he would also scrape off the film.^85^

Yeldall advertised a reward for the return of a 19-year-old “negro man.”^246^ In May 1775, he reported an ophthalmic testimonial, of a “film” taken off an eye.^247^ He also had performed an operation for “a Hare-Lip” (cleft lip).^247^ The next year, Yeldall published the testimonial of William Bell, who had been in the care of “a late famous eye Doctor’s hands” (perhaps Graham) without relief, until he saw Yeldall “who in one minute brought me to sight, and I can now see to ride.”^248^ In the Spring of 1777, Yeldall published testimonials of Peter Goslen and John Bell, 2 residents from west of Lancaster whom Yeldall “in one minute brought me to sight.”^249^ Bell had learned of Yeldall’s services when he saw the newspaper reports that his brother had been cured.^249^ We do not know for sure if any of these cures were for cataract or a “film” on the eye, but Yeldall continued to advertise that “the doctor couches cataracts in the eyes and restores sight to the blind in one minute” and claimed to be able to prevent cataracts, through 1780.^250^

Perhaps, the fact that Yeldall still referred to “the United Colonies” in August 1776 (after the Declaration of Independence) was a harbinger of his loyalist sympathies and ultimate fate.^248^ Finally, in November, Yeldall realized he was now in “the United States.”^251^ Some sort of proceedings were attempted against him in the fall of 1778; however, he was “discharged by proclamation.”^252^ He continued to advertise his businesses in Philadelphia through September 1780,^253^ but the next month, Yeldall and Benedict Arnold were proclaimed to be guilty of “high treason” (for completely separate offenses).^254^ Yeldall’s lands were seized by the state.

Yeldall fled to New York and announced a lecture in which “a figure is prepared in wax” showing the nervous system, and “. . . a large Eye is prepared furnished with all the Coats and Humours belonging to that most noble organ.”^255^ He closed with “a dissection of the natural Eye” showing “the Retina, or Expansion of the Optic Nerve over the bottom of the Eye, by which we receive the inestimable blessing—Sight.”^255^

Presumably, Yeldall fled to England before the British evacuated New York in 1783. In 1790, he advertised in New York his “Load-Stone & Magnet” for “speedily removing all curable Diseases,” which he claimed was used throughout Britain^256^ and indeed had been advertised there the previous year. Although not part of the mainstream medical establishment, Yeldall was important as one of the few to advertise and teach about cataract and other eye surgery during the Revolution.

Isaac Calcott of Hamburg practiced in London before arriving in Providence in 1769 at the age of about 30 years.^257,258^ He was the seventh son of a seventh son^257,258^ and treated many conditions, including “sore eyes.”^258^ If patients could not travel, they could send their first morning urine to be evaluated.^258^ Calcott was “decently dressed,” but “the Faculty dispised him as a German Quack.”^257^

Calcott treated a 7-year-old boy named Elizur Belden in about 1770.^257^ The boy had a fever since he was born, which was attributed to worms.^257^ At the age of 2 years, he began to get leg pain, which limited him to crawling, and inflamed eyes, with a conjunctival discharge, and after a year, lost his vision.^257^ About a month before, these symptoms started, he had fallen on the ice, but his parents did not believe the fall to be related.^257^ Around the time of his third birthday, his father took him to a pool at New Lebanon, and also to Boston, where the doctors put a powder in his eyes, which caused him a great deal of pain.^257^ Despite these treatments, he lost all vision.^257^ He had a protruding belly and “the thigh bone at the hip dislocated.”^257^

Calcott treated the boy at the family home. Before the treatment, the boy was fearful as he sat on his mother’s lap because of his previous experience with doctors. Calcott asked his mother “whether she had Faith . . . that God could give more to one Man than to another?”^257^ When she responded in the affirmative, Calcott prayed and “licked the eyes, first putting his Tongue into one Eye & then into the other Eye of the Child” for less than a minute “and instantly the Child saw, & ever after continued to see well.”^257^ Calcott said one eye was perfectly cured and the other partially so.^257^ Calcott licked the boy’s eyes again several times over the next few weeks, but all the effect came with the first treatment.^257^ The boy’s photophobia was resolved. The treatment seemed to make the doctor fall ill.^257^ The day after the first treatment, Calcott provided the boy with a worm powder.^257^ A little over a week later, a 10-ft-long tapeworm “came away” (was excreted) with a reduction in the protrusion of the belly.^257^ The boy was healed, except “Lameness” which required “a staff.”^257^ He still always had “a weakly Constitution,” and in 1786, he “was seized with a Bleeding from his Breast” (perhaps hemoptysis), and soon perished, at the age of 23 years.^257^ His parents recounted his story to Ezra Stiles, who officiated at the funeral.

We may never know what conditions affected the boy. Cysticercosis from tapeworm infection can cause hemoptysis,^259^ intermittent vision loss,^260^ with distribution to muscle tissue or the spine.^261^ Perhaps, he had multiple conditions. This case suggests that treatment with saliva might have been a component of Old World traditional medicine.

Calcott was admonished by Mrs Belden for his alcoholism, and he wept, but said he could not control this “Vice.”^257^ In Middleton, he was “found drunk in the street—the Boys tarred & feathered him,” whereupon he was not seen again in Connecticut.^257^ He advertised in Boston and Portsmouth in 1773.^262^

### John Bartlett in Charlestown in 1769

**Figure 13.**
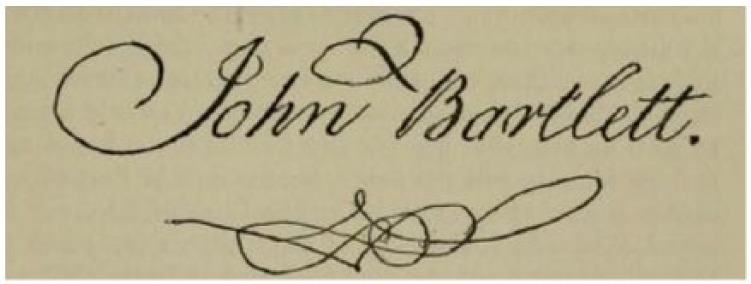
Signature of John Bartlett (1730-1795) of Rhode Island.^263^

An important step in the transmission of medical techniques was the performance of procedures by physicians born and trained in the New World. The earliest native-born and locally trained cataract surgeon identified to date is John Bartlett (1730-1795; Figure 13).^263^ Although others might have preceded Bartlett, he today is the earliest known in some measure of his prominence and pioneering spirit. His biography has never been written.

Bartlett was a fourth-generation Mayflower descendent.^264,265^ His parents, Josiah (1701-1782) of Marshfield and Mercy, moved to Lebanon Connecticut in 1726.^264,266^ Little is known about his father Josiah Bartlett (no relation to the signer of the Declaration of Independence) except that he owned a farm,^264^ was commissioned as a captain in the Goshen trainband (a local militia) in 1750,^267,268^ and provided salt to the Continental Army during the American Revolution.^268^

Medical training was often conducted within an apprenticeship. We do not know with whom John Bartlett received his medical education. The major surgeon in nearby Norwich was Joseph Perkins, who went to college at Yale.^269^ Bartlett spent 4 or 5 years in North Yarmouth (now in Maine) and married Susanna Southworth of that town in 1753.^265,270^

During the French and Indian War, Bartlett served as regimental surgeon in the third regiment of Connecticut from May 11 to November 20, 1758.^271^ The regimental officers serving with Bartlett included Eleazer Fitch, Benjamin Hinman, and Israel Putnam.^271^ Hinman, Putnam, and presumably the entire regiment served in the theater around Lake George.^272^ This theater involved heavy fighting with the French and their Native American allies. Smallpox was rampant.^272^ Putnam himself was captured in August 1758 and nearly burned alive by “the Indians” before being rescued by a French officer.^272^

After Bartlett’s wife died, he married Lucretia Stewart in March 1761 in North Stonington.^273,274^ In December 1767, Bartlett operated on a 50-year-old man with an inguinal hernia, or “Enteroepiplocele,” colloquially “a Burst,” because “His Intenstines and Cawl had been fallen down into the scrotum.”^275^ Bartlett made an incision “in the Groin” which allowed “the Intestines” to return to the abdominal cavity.^275^ Then, Bartlett had “the Wound closed up with interrupted Suture.”^275^ The patient recovered fully. In the era before antisepsis or anesthesia, this operation would have been difficult and risky. Hernia surgery was not generally available. In 1772, a Long Island man perished of inguinal hernia without operation, though one was considered.^276^ Bartlett’s hernia procedure may have been the earliest reported in the colonies, but it was not the first performed. Joseph Perkins had performed a surgery for inguinal hernia (“bubonocele”) on Abiel Stark of Lebanon in 1764.^269,277^ On the death of Stark in 1770 (from an unrelated stroke), the original surgery and autopsy were reported.^269^

In 1769, Bartlett reported a lithotomy on a 7-year-old boy, several amputations, and “by couching Cataracts of the Eyes, has restored several to their Sight, from a State of total Blindness.”^168^ On April 15, 1770, Bartlett and his wife transferred their membership from the Congregational Church in Westerly to that led by Ezra Stiles in Newport.^274^ On April 27, Stiles prayed with Capt. Pollipus Hammond, before “Dr. Jno Bartlett performed upon him the Operation of couching or depressing a Cataract in his Eye.”^274^ Stiles witnessed the surgery.^274^

In January 1771, Bartlett performed the autopsy of a 17-year-old boy who died with calcifications in the kidney.^274,278^ Bartlett bemoaned the rarity of autopsies:

But, from a strange I know not what superstitious Veneration for the Dead, few Persons, in this Part of the World, have hitherto been induced to consent to an Operation of this Kind; and thus many Lives have been early lost . . . .^278^

The boy had passed a kidney stone at 4 years of age but was then asymptomatic until his 17th year of life.^278^ The young man had been “so great a Water Drinker” that he kept water by his bedside at night. As a brother had passed away of the same disorder, Bartlett investigated and found that the well water created a “calculous concretion” on the inside of the family tea kettle.^278^ Bartlett believed that the well water contributed to and accelerated the renal disorder. As we will see later, Bartlett seemed interested in environmental conditions. In 1772, Bartlett sailed with 14 persons to Dodges Island, Connecticut, to perform smallpox inoculation because this treatment was illegal in Newport.^274^

As the American colonies progressed toward revolution, some idea of Bartlett’s antipathy toward British royalty may be indicated by the fact that in 1775 he named his newborn son Oliver Cromwell.^274^ Oliver was 1 of 3 sons who became physicians.^279^ In October 1775, Bartlett traveled to Connecticut, presumably to provide medical coverage for military activities.^274^ In February 1776, Bartlett returned to Newport, where he was “appointed Chief Surgeon of the Brigade” of the Rhode Island militia.^274,280^

Given Bartlett’s leadership in major surgeries, wartime medical experience, and revolutionary fervor, it was natural that in April 1777, he was appointed^281^ “Physician & Surgeon General of the Army in the Northern or Ticonderoga Department.”^282,283^ He reported in July and ultimately served under Maj. General Horatio Gates.^282,283^

By all accounts, he did not succeed in this appointment. On August 18, Bartlett’s flying hospital had 335 patients.^284^ The regimental surgeons complained that Bartlett refused to provide adequate supplies (Bartlett denied this accusation),^284^ and that he claimed to be “too old and infirm” to fulfill his duties (at the age of 47!).^283^ Bartlett wanted 3 more surgeons^284^ and called surgeon Thomas Tillotson “my secret enemy” because he believed Tillotson was plotting against him.^263,283^ At the end of August, the hospital had 192 patients. The regimental surgeons said that General Gates had forbidden them to help Bartlett.^284^

**Figure 14.**
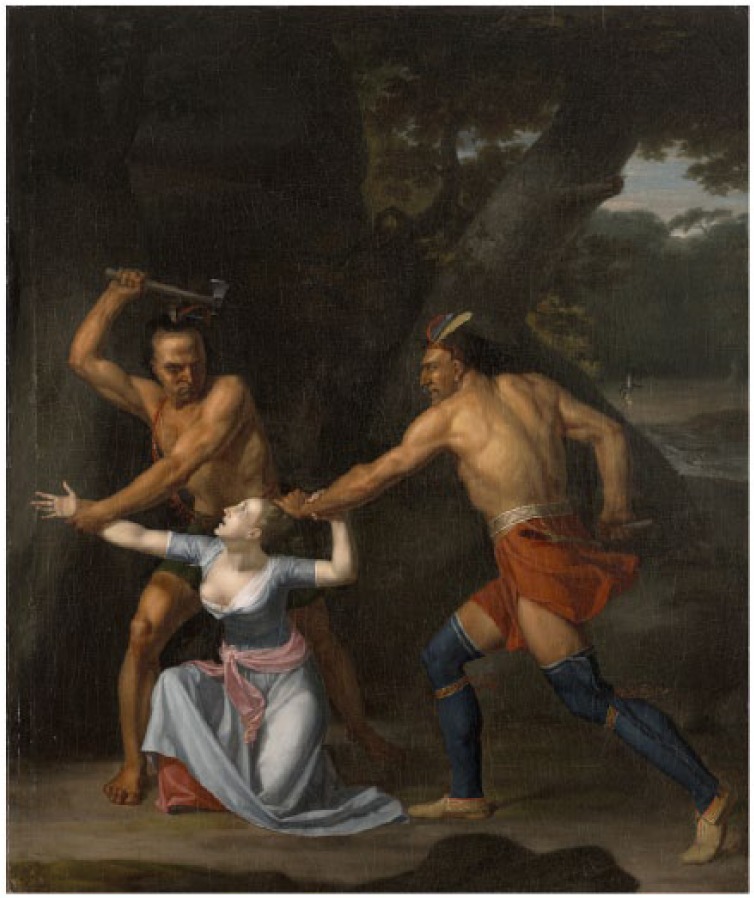
The Murder of Jane McCrea (reported by John Bartlett in 1777), painted by John Vanderlyn in 1804.^285^

Bartlett’s most notable act was to report on July 27, 1777 that the 25-year-old American woman, Jane McCrea had been killed and scalped by Indians allied with the British general Burgoyne (Figure 14):^285^

I have this moment returned from Fort Edward, where a party of hell-hounds, . . . with . . . the British troops, fell upon an advanced guard . . . Poor Miss Jenny McCrea and the woman with whom she lived were taken by the savages, led up the hill to where there was a body of British troops, and there the poor girl was shot to death in cold blood, scalped and left on the ground . . . The alarm came to camp . . . I immediately sent off to collect all the regular surgeons, in order to take some one or two of them along with me, but the devil a bit of one was to be found. There is neither amputating instruments, crooked needle, nor tourniquet in all the camp. I have a handful of lint and two or three bandages, and that is all. What in the name of wonder I am to do in case of an attack, God only knows. Without assistance, without instruments, without anything!^286^

Although scholars have debated the circumstances of her death, the news is believed to have galvanized American resolve.^287^ The subsequent surrender of Burgoyne’s army of over 6000 men on October 17, 1777 following the second battle of Saratoga marked a turning point in the war. Although Bartlett’s son Charles was just 11 years old, he later wrote that he began his medical career with his father during this campaign.^281^ There is no way to verify this claim.

On October 22, 1777, Bartlett left Albany with General Gates’ permission.^263^ He indicated this was “for the recovery of the use of my arm which was badly fractur’d.”^263^ After his recovery, he worked in 1778 at the flying hospital at White Plains.^263,283^ In the summer of 1779, Shippen, the Director General, ordered Bartlett to superintend the hospital at Fishkill, New York.^263,282,283^ However, when the hospital officers refused to follow his orders, he returned home in September 1779.^283^

After his service, he settled in Charlestown.^282^ Yale University, under Stiles’ leadership, granted Bartlett an honorary “Degree of Doctor of Physic” in 1779.^282^ He moved to Nantucket in 1783,^274^ where he lived a quiet life. Although Congress denied him payment of back pay in 1781, Bartlett again wrote in 1792 to William Ellery, a signer of the Declaration of the Independence, requesting additional payment for his military service.^263^ Ellery supported these efforts.

Records provide some indication of Bartlett’s personal circumstances. In 1772, Stiles had “baptized Peter a negro Infant Servant” of Bartlett.^274^ Bartlett was active in the Congregational Church and was “a sensible and firm Believer in Revelation; understanding the Doctrines of Jesus in the sense of the Calvinists.”^274^ He wrote about the waters around Nantucket. He had always had a “Hygrometer” which indicated the humidity.^274^ Bartlett died in 1795.

### Northern New England, 1770-1777

**Figure 15.**
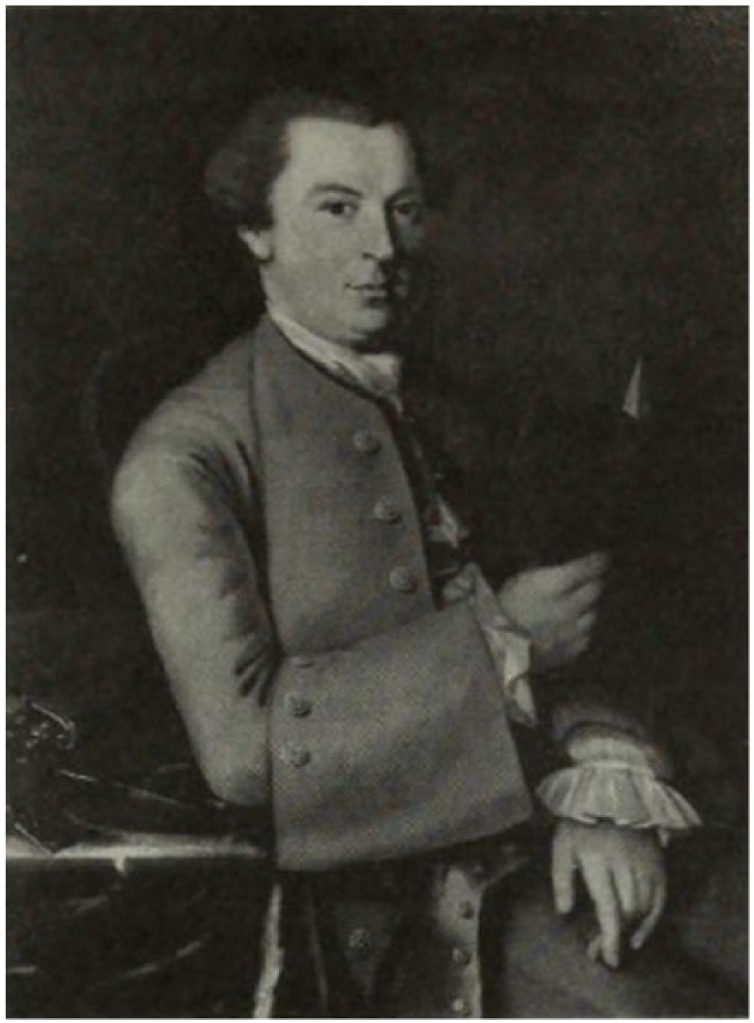
Hall Jackson (1739-1797) of Portsmouth, New Hampshire. Painting attributed to John Singleton Copley.

After Bartlett, several American-born surgeons began to couch cataracts, but their ophthalmic activity ceased during the war years. Hall Jackson (1739-1797)^58^ was born in Hampton, New Hampshire, and learned medicine from his physician father, Clement Jackson, after they moved to Portsmouth. Hall Jackson spent 1762 in London, possibly at Middlesex Hospital.^58^ Jackson then returned to Portsmouth, New Hampshire, to open an apothecary shop (Figure 15).^58^ Jackson seems to have shifted his focus toward medical practice at some point; however, the apothecary shop was still advertising in 1774.^58^ He reported bilateral leg amputations on a 17-year-old boy who fell into a frozen pond in 1768.^58^ Another leg amputation by Jackson was performed the same month.^58^ In September 1770, the newspaper reported that Jackson couched the cataracts of a 60-year-old “Negro Man” who had been “totally Blind” for many years and was “instantaneously restored . . . to Sight.”^58^ A 2-month interval separated the procedure on the second eye.^58^

Jackson reported additional couchings in December of 1771.^58^ A 39-year-old man who lost vision at the age of 6 months was bilaterally couched, with a month between operations. The first eye couched suffered a great deal of inflammation due to a history of trauma. After the couching of the second eye, he “was able clearly and distinctly to see the many Persons and Objects that surrounded him.”^58^

The hiatus between his return from London and multiple publications regarding cataract suggests that Jackson did not immediately begin couching on his return. It is possible that some practitioners would be exposed to a particular procedure in Europe but would not practice the procedure until others in America had led the way.

Jackson performed inoculations at the Essex Hospital. The accidental discharge of a cannon near the hospital in late 1773 severely injured one Captain Lowell, who “. . . having recovered, the Cure merits Notice, and does great Honour to the Physician (Dr. Hall Jackson, of Portsmouth,) who has the Care of the Hospital.” The mangled right hand was amputated below the elbow, his neck and jaw were injured, and “The Coats of the right Eye pierced and its Humours discharged, & the Bone between the Eye and the Nose broken through; the other Eye greatly hurt . . .”^288^ The patient “recovered as to need no further care of a Surgeon.”^288^

Jackson’s father also trained Stephen Little (1745-1800), who married Jackson’s sister Sarah.^58^ Little had gotten his start selling medicines in Portsmouth^289^ and established his medical practice there.^58^ He briefly advertised cataract couching in New York in 1772.^290^ There, he couched the cataracts of a 72-year-old woman who was “instantly restored to sight” after 6 years of being “totally blind.”^291^ However, during the revolution, Little, a loyalist, was banished from New Hampshire.^58^ He escaped to British protection in 1777 and to London in 1778.^58^ In London, he worked first as a surgeon and then as an apothecary.^58^ His wife and children never joined him.^58^

Benjamin Church, Jr (1734-1778) of Boston^58^ was descended from an Englishman who immigrated to Massachusetts in 1630.^169^ Church was educated at Harvard College.^169^ He apprenticed in Massachusetts^169^ and under a “Doctor Pynchon,”^292^ whom we suspect might have been Charles Pynchon (1719-1783) of Springfield, Massachusetts.^293^ (Later sources incorrectly placed “Dr. Charles Pynchon” in London.)^169^ In 1757, Church worked briefly as the surgeon on a sloop-of-war out of Charlestown.^169^ Later that year, he began studying medicine at the London Hospital for 3 years, before returning to Boston.^169^ During a smallpox epidemic in 1764, Church inoculated John Adams and then a attorney.^169^

Church had some interest in the eyes. In June 1773, he performed “couching upon the eyes” of one 56-year-old “Mrs. Hodges” who had been blind for several years, permitting her to distinguish colors.^169^ He also treated the eyes of John Adams. In December 1774, Adams noted “an Inflammation in my Eyes” which prevented him from reading or writing.^294^ In June 1775, while in Philadelphia as a delegate to the Second Continental Congress, Adams wrote his wife “Dr. Church has given me a Lotion, which has helped my Eyes so much that I hope you will hear from me oftener than you have done.”^295^

Readers of Esther Forbes’ historical novel *Johnny Tremain*, set in pre-revolutionary Boston, will know not only of a “Dr. Church” but also of a “Dr. Warren.”^296^ The real physicians of the Warren family played important political and medical roles during the revolution. Joseph Warren had trained his younger brother John.

Church and Joseph Warren served together on a number of revolutionary groups: The Long Room Club of 1762, a committee of correspondence in 1768, another 1768 committee to guide Boston’s representatives to the general court, the 1774 and 1775 delegations to the Provincial Congress, a 1774 committee to inspect Boston commissaries for surgical and other supplies, a 1775 committee to collect taxes, and another in 1775 to furnish medical supplies for the province.^169^ But the paths of Church and Warren would ultimately diverge.

The lead-up to war began with the Boston Massacre of 1770, when British soldiers fired on a crowd, killing 5.^169^ Dr Church performed the autopsy of Crispus Attucks, the first killed.^169^ Two soldiers were found guilty of manslaughter and were branded with an “M” for murder on the right thumb.^169^ An annual oration to commemorate the massacre was given by Joseph Warren in 1772 and by Church in 1773.^169^

On June 17, 1775, the British captured Bunker Hill (and neighboring Breed’s Hill) from the American militia, led by Putnam. Joseph Warren was killed when shot with a musket ball in the head. His brother John treated the wounded from the battle. Some scholars doubt the traditional story that John went to the area to find his brother and was bayonetted by a British sentry as a warning to stay away. John Warren served as a military surgeon at the Battle of Long Island and elsewhere during the war.

Church took command of the hospitals of the Continental Army in August 1775.^58^ He oversaw hospitals which had been the homes of loyalists who had fled to British protection in Boston,^58^ such as the Hallowell House in Roxbury.^169^

On June 19, 1775, Jackson traveled to Cambridge to treat some of the 271 wounded.^58^ Jackson wrote,

Dr. Church having got notice of my being at Mistick, the best surgeon on the Continent being obliged to supply poor [Joseph] Warren’s place at the Congress forced the principle of the wounded on me, I went on with this fatigue 15 days, when a violent inflammation in my eyes forced me to return to Portsmo.^58^

Jackson “amputated several limbs and extracted many balls.”^58^ Jackson was concerned that in his absence, his hometown would see “. . . Doctors Cutter and Brackett & Little running away with all my business at Portsmouth.”^58^

It turned out that the ostensible patriot Church had been spying for the British. An encrypted letter from Church to the British was intercepted. Putnam brought the woman caught with the letter to Gen. George Washington, who threatened her with hanging, after which she confessed that Church was the letter’s source.^169^ Ezra Stiles offered to decrypt the letter, although this task was ultimately performed by others.^169^ Surgeon James McHenry (1753-1816) informed Church that his code had been broken.^169^

In October 1775, the American General John Sullivan wrote of “. . . Dr. Church’es having been detected having a Treasonable Correspondence with the Enemy . . .”^58^ Sullivan had long suspected Church of disloyalty based on his poor medical care: in Sullivan’s opinion, Church’s outcomes were substantially worse than those of his peers. He described,

Mr. Simpson, who was shot in the foot—an amputation was necessary—Doctor Jackson, who every one must allow to be Infinitely his Superior, was there, & had every thing prepared to take off the Limb—Doctor Church happened to come in—forbid him to proceed . . . .^58^

Ultimately, the patient died. Another patient had an amputation performed by Jackson, and 4 days later, Jackson objected to the patient being moved, but Church insisted, and the patient died.^58^

Jackson, who had served without an official appointment or salary, himself described the betrayal by Church:

Church pretended to be my friend, was mighty sorry that I was left out of the general hospital . . . all the time the dirty dog was plotting against me, and was as great an enemy to me, as he was to his own Country . . . .^58^

Gen. Washington turned Church’s fate over to the Provincial Congress, which voted for imprisonment. McHenry had himself been imprisoned by the British after the capture of Fort Washington in Manhattan. A 1777 proposal to exchange Church for McHenry made some progress but was ultimately nixed.^169^ Eventually, in 1778, Church was simply exiled. He sailed for Martinique, but the ship was lost at sea.^169^ John Adams lobbied for Church’s replacement to be Jackson, but political considerations resulted in Dr Morgan of Philadelphia being selected.^58^

Jackson’s greatest medical contribution was the introduction of digitalis therapy to America.^58^ After 1774, Jackson does not appear to have resumed cataract couching, based on his private journals covering 1774 to 1795,^58^ perhaps because of the establishment of cataract surgery in the Northeast by other physicians. One such physician was John Warren, as discussed below.

### Francis Mercier (Couching in 1774)

In 1772, a French surgeon named Francis Mercier was observed near London walking 2 horses.^297,298^ In addition to England, Ireland, Portugal, and his native France, he had traveled through and acquired some familiarity with the languages of Germany, Spain, and Italy.^298–300^ He was the son of the postmaster of Toul, France.^301^

Mercier wore “a light coloured coat and waistcoat, a pair of new buckskin breeches, a pair of boots, a new brown great coat and red collar, and he had very much the appearance of a gentleman.” An observer was suspicious because one horse was unshod, except for a partial shoe on one foot. The Frenchman claimed that he was buying horses for the French king and explained that he would have already returned home, except that “he had met with two hussies last night who kept him up all night.”^298^ The observer’s suspicions grew when Mercier could not describe the steep descent of the road from the town of Harrow.^298^ At the subsequent trial for thievery, the French surgeon claimed that a friend from Ireland had sold him the horse. The horse had only one functioning eye, but Mercier claimed he made the purchase “in hopes I could give him the sight of one eye; I tried but could not do it.” Mercier was sentenced to death.^298^ His jailers remembered that

. . . he artfully contrived to cut a Kind of Channel round the lock of his Cell-door, in such a Manner that it might have been pushed out all together, and had filled it up with chewed Bread, and the Dust of the Floor, to prevent its being observed.^302^

Despite these efforts, Mercier did not escape, however. Instead, he was granted clemency by King George III and sentenced to exile in America, with 7 years of servitude.^303,304^ In August 1773, he arrived in Maryland on a convict ship sailed by one Captain McCullough (McCulloch).^305–308^ On board, he distinguished himself by providing medical care.^307,308^ He befriended not only the captain but also Daniel Chamier, a passenger and Maryland merchant.^308^ Captain McCullough appreciated the medical care provided by Mercier. The authorities released him from the required period of servitude, and the captain provided him with the essentials to start his career as a surgeon.^307,309^ Soon afterward, the captain fell sick, and, despite their friendship, many suspected that the captain had been poisoned by Mercier, a fact recalled even by his friend Chamier.^307,308^ After McCullough’s recovery, some of McCullough’s belongings were found in Mercier’s possession.^307,309^ Mercier went into medical practice, prescribing medicines he had invented in England to cure a merchant Mr Kaiser of kidney stones.^299^

But the captain’s support must not have been enough. Mercier was soon accused of nighttime robberies of Doctor John Boyd’s house and Patrick Kennedy’s apothecary shop in Baltimore.^306–309^ To enter Boyd’s house, he (and possibly unknown accomplices) “. . . forced their Way thro’ a Window, by first boring a Shutter with a Gimlet . . . introducing a small Saw” to make a hole “large enough for the Admission of a Finger, by which the Key that secured the Window, was pushed out . . .” Mercier stole clothing, surgeon’s instruments and pistols.^306^ From Kennedy, he stole “Keyser’s Pills, . . . British Oil, Turlington’s Balsam, Jesuits Drops . . .”^310^ He was found guilty and again condemned to hang.^307,308^ But Mercier wrote a petition asking for clemency, which Chamier passed on to Royal Governor Sir Robert Eden.^307,308^ The Governor exiled Mercier from the province.^308^ At first, Mercier tarried and threatened his prosecutors, but he ultimately fled the authorities.^307^

Mercier practiced in cities up and down the East coast over the next few years. One account listed Halifax among his destinations.^307^ On his arrival in Philadelphia in January 1774, Mercier changed his name and announced that “Doctor Louis” from Paris was establishing a practice.^87,299^ Although he claimed expertise in surgery, his emphasis seemed to be medical.^299^ He advertised a “worm-repulsing powder for children.”^299^ Although ophthalmology was just a fraction of his practice, he treated “some ailments of the eye [Gebrechen in den Augen]” and had “an eye-water [Augenwasser] that will quiet some types of eyes.”^299^

Unfortunately, Mercier’s tenure in Philadelphia ended in April 1774 when he stole a horse:

The thief, who is the noted Doctor Louis a Frenchman is a tall well made man, has a smooth and full face, wears his own hair, curled on both sides of his head, and tied behind. He wears a suit of light blue cloth.^300^

Mercier had

several very good French books of physic and surgery, likewise a fine set of surgeon’s instruments. He has the scar of a cut over his left eye, and another small scar in his upper lip, and dresses gentleman-like . . . He . . . speaks Latin very well.^300^

In April 1774, “Doctor Louis” advertised in Newport that he had just come from Philadelphia and “practices Physic and Surgery.”^311^ He cures “cataract, gout, serena [gutta serena], fistula lacrimalis, purls, unguis, optalmies, redness, and any disorders in the eye . . . hair lips, scall on the head, scurvy in the gums . . .”^311^ “Doctor Louis” also sold “teeth powder, which makes them as white as snow . . .”^311^ He noted that “. . . since he has come into North America he has given sight to 27 persons in Philadelphia, 7 in Baltimore, and 5 in New York”^311^ but we have no way to confirm these numbers.

Mercier advertised as “Doctor Louis” in Newport in early May 1774.^312^ Also, in May 1774 in Providence, he advertised,

Doctor Louis . . . will..couch the Eyes of Elizabeth Donaldson . . . any Person inclined to see the Operation may attend. A Negro . . . that has been two Years blind, was operated upon by the Doctor last Thursday; and there is great Hopes that he will recover his Sight, as he could the next Day perceive some Objects.^313^

Later that month, he published testimonials in Boston and noted that after Donaldson’s surgery, she “recovered the sight of an eye.”^314^ In June 1774, “Doctor Louis, Oculist and Dentist” advertised in Salem.^315^ Unfortunately, the next month, “the well known Quack, Dr. Louis” was “committed to Gaol” for “breaking and entering” in Cambridge.^316^

The Americans later claimed that through the ministrations and advocacy of Chamier (the merchant, who ultimately became the Commissary General of the British Army in North America), Mercier secured a position as a surgeon for the British army.^307^ Two days after the Battle of Bunker Hill on June 17, 1775, Mercier was conducting wagons with wounded soldiers when he was captured by the Americans and held as a prisoner in Cambridge.^307^ Mercier asked for laudanum to help him sleep, but he used it instead to incapacitate the “centinels” and escaped.^307^

In August 1775, the New Haven advertisement of “Doctor L. Boduin, Occulist and Dentist from France . . .” was essentially identical to that of Doctor Louis.^74^ But in September 1775, “Dr. Boduin” was forced to “decamp” after he stole from an apothecary shop.^317^ The townspeople realized that “He is the same person who advertised in the last New-London paper, by the name of Louis; he is supposed to be an old offender, and will change his name wherever he goes.”^317^

He turned up in New York in October 1775 as “Doctor Dubuke, occulist and dentist, just arrived from Boston,” with an essentially identical advertisement.^318–320^ He was able to continue practicing through early January 1776.^320,321^ He advertised “stomachic pills.”^321^ Again, he was caught stealing because in March of 1776, the papers noted,

The famous Dr. Dubuke, a Frenchman, who was branded here last January term, for stealing indigo &c. departed . . . in the Amboy stage boat, to visit Philadelphia . . . He professes himself a denist, and has travelled through the Eastern colonies under various names.^322^

Branding, sometimes on the hand, was used to permanently mark criminals.

“Doctor L. Butte. . . Surgeon Dentist” advertised beginning in mid-March, 1776, in Philadelphia.^87,323^ He “sets artificial teeth” and has “tooth powder, which cures the scurvy in the gums, and makes the teeth as white as snow . . .”^323^ Butte remained in Philadelphia for several months^87,324^ and advertised his practice there on July 4, 1776.^325^ According to later (possibly apocryphal) American accounts, Mercier claimed that in the American flying camp, established by Gen. Washington near Philadelphia in the latter half of 1776, the well water was poisoned, leading to the death of 3000 rebels.^307,326^

The Americans later claimed that Mercier was a co-conspirator at meetings in New York for a conspiracy to kill Gen. Washington and Israel Putnam, and that Mercier attended the execution of one of the conspirators. According to this account, Mercier was recognized as a surgeon who had worked for the British and was imprisoned.^307^ Indeed, a soldier and member of Washington’s guard, Thomas Hickey, was executed in New York on June 28, 1776. The rebels suspected that Hickey was part of a conspiracy to kill Washington and Putnam by poisoning.^327^ It cannot be determined whether Mercier could have participated in the plot. It is always possible that the publication of advertisements could be arranged in advance. For instance, the July 4 advertisement in Philadelphia could have been arranged before June 28.

This question brings us to the curious matter of the “French Doctor Blouin.” Blouin began advertising in New York in September 1775, just before Mercier began advertising as Dubuke. Sometimes the advertisements of Blouin and “Dubuke” appeared right next to each other in the paper.^328^ Although Dubuke was an oculist and dentist, Blouin’s emphasis was initially medical, and he prescribed “Keyser’s pills,” which of course had been stolen by Mercier in Maryland. After Dubuke was branded for thievery in January 1776, Blouin complained that “evil minded persons” were suggesting that he (Blouin) was actually Dubuke.^329^ Blouin began to address ophthalmic complaints. Blouin had a “Universal Powder” which cured “sore eyes” and “worms in children” and “a pearl in the eye and dimness of sight.”^329^ Blouin also had “stomachic pills” and an eye water.^329,330^ Blouin advertised in New York more regularly after Dubuke was branded for stealing. However, shortly after Dubuke was evicted from New York in March 1776, Blouin’s New York advertisements stopped until May.^331,332^ Shortly after June 28, when the Americans claimed Mercier was arrested, the advertisements of Dubuke and Mercier stopped forever. Could Blouin have been an alternate identity of Mercier? In this scenario, Mercier initially assumed the identity of a medical practitioner when the hunt for an escaped surgeon was intense. He adopted a second, more surgical persona (Dubuke), once the heat died down. After Dubuke was evicted to Philadelphia, Blouin, of course, would have stopped advertising. But Blouin’s advertisement in May would suggest that Mercier could have appeared in New York intermittently that spring and formed part of the conspiracy against Washington. Ultimately, we do not know if Blouin was Mercier. All we can say is that a French doctor who was rumored to be Mercier and who used similar medicines did advertise in New York that Spring.

The Americans later alleged that Mercier escaped from them and helped to guide the British during their invasion of Manhattan.^307^ But by most accounts, he was still in the custody of the Continental Army for his crimes when the British captured Fort Washington.^308,326^ The British held Mercier and needed to determine his fate.^308^ Chamier, a loyalist who served as the commissary general in North America (logistics), admitted to seeing Mercier in British custody but denied advocating for him.^308^

What is agreed by all accounts is that the British installed the French doctor Mercier as director of the hospitals for American prisoners.^307,326,333^ The British directors in New York justified this appointment by saying that they found “Louis Debute” among the “rebels” and assumed that he was one of them.^333^ The Americans alleged that he killed numerous prisoners and stole their property.^307^ Routinely, Mercier would predict that 5 or 10 soldiers under his care would be dead by morning, and they invariably died.^326^ That the “physick” he gave to patients was poison was proved by soldiers giving it to a dog, who died immediately.^326^ Another American account held that on “Jan. 4, ’77 . . . .The doctor gave poison powders to prisoners, who soon died.”^334^ Ethan Allen also reported the high prisoner mortality was “in consequence of a slow poison.”^335^ Allen estimated that “two thousand perished with hunger, cold, and sickness, occasioned by the filth of their prisons, at New York.”^335^ Another American, John Pintard, stated that “the prisoners . . . were all indiscriminately huddled together, by hundreds and thousands, large numbers of whom died by disease, and many undoubtedly poisoned by inhuman attendants for the sake of their watches, or silver buckles.”^335^ Rumors of poisoning were not restricted to Mercier’s tenure. When Thomas Stone and his fellow prisoners were sickened after eating bread in 1778, they assumed it was poisoned^336^ because they recalled that “. . . the prison that the prisoners taken at Fort Washington had been poisoned in the same way.”^335^

The prisoners were given just a single shirt in the middle of winter.^307^ When they died, “the infected cloaths” were given to subsequent prisoners, which “encreased the pestilential disorder” so that 573 prisoners died in 5 months.^307^ The prisoners were denied adequate water.^333^ According to the Americans, Mercier exclaimed “The Rebels died like fun.”^307^ Presumably, the high mortality in the hospital was due to the overall conditions of crowding, clothing, food, etc, which were determined at a higher level than this single French doctor. However, in the American account, the British General Howe was surprised at the high prisoner mortality rate and sought answers from Mercier.^307^

The British also took prisoner James McHenry, a 23-year-old surgeon. McHenry railed against the treatment of prisoners by doctor “Louis Debute” (Mercier) in a letter to Gen. Washington.^333^ McHenry noted that Debute was “notorious for crimes” even before the Continental army evacuation of New York.^333^ McHenry successfully lobbied to prevent Debute from prescribing medicines but was initially unable to get him dismissed altogether.^333^ Finally, a British officer reported that Debute struck an American prisoner with his stick (cane), resulting in the man’s death 15 minutes later.^333^ Soldier Jabez Fitch of Connecticut also reported that “Doctr Debuke . . . often made application of his cane among the sick.”^337^ Due to the exigencies of war, the British were unable to try Louis Debute for murder, but they were able to get him dismissed from the hospital.^333^

In early 1777, Mercier assumed at least one more identity in New York: John Dupuis, a surgeon and dentist “lately from France.”^338,339^ Then, he returned to England and served as an interpreter to a fellow French speaker, a jeweler named Mondrey.^340^ At the home where the jeweler lodged, there was a party, with drinking, singing French songs, and playing cards.^340^ After the inhabitants had retired for the evening, Mercier took from his coat “a kind of tomahawk” of his own design which a cutler had constructed for him, approached the sleeping Mondrey, and “shattered his skull to atoms.”^341^ He stuffed the friend’s body in a small trunk, stole some money and valuables, and departed.^340,341^ With the money, he was able to set off for the suburb of Richmond “accompanied by a woman of the town.”^341^ Mercier then called on the home every few days, asking if Mondrey had returned from the country.^340^ The family became suspicious, and got a ladder to climb through the window, and found the body in the trunk.^340^

Mercier confessed to murder at a preliminary hearing in October.^301,342,343^ He was tried on December 3, 1777.^344^ The night before his trial, Mercier drank wine and said that if his health had been what it was 3 months previously, he would have been able to present his own defense in court.^344^ The next day, when asked how he pled, Mercier refused to answer. Testimony was offered that Mercier could hear and could speak English. A surgeon in the court examined Mercier and reported that “his tongue is rather moist.”^344^ The jury was asked to determine whether Mercier “stood mute through obstinacy, or by the visitation of God.”^344^ The jury settled on the former. Mercier was sentenced to death, with his body to be “dissected and anatomized.”^344^

Just before his scheduled execution, he sent a messenger to request an amount of opium “in Quantity to have destroyed six persons” but the apothecary refused to make the sale.^345^ He requested a shave from a barber, but was unwilling to have his hands tied, so the barber was sent away.^345^ Mercier was granted a visit from a priest, but when Mercier was told he could not travel to the chapel, he declined to speak to the priest.^345,346^

On December 8, he was taken to the gallows. One account stated,

His Behaviour . . . was decent and composed . . . The strongest Marks of sincere Repentance were visible in his Countenance; and he seemed to die with such a calm Tranquility of Bodey and Mind, as bespoke a modest Assurance that his Sorrow and Sufferings would attone for his Guilt . . . .^302^

According to another account, he proclaimed his adoption of the Protestant faith and admitted to robbery of the jeweler but not murder.^345,347^ After a cover was placed over his head, he begged, and was granted permission to pray for a quarter of an hour.^347^

The news of Mercier’s execution returned to the American colonies. An account mixing truth with speculation was the lead story on the front page of the Pennsylvania Gazette and then traveled through the rebelling colonies.^307,348-350^ The British, for their part, reported that their records showed that they employed no one by the name of Francis Mercier in their hospitals.^308^ This statement proves nothing because of course their records would have listed him as Louis Debute. In death, Mercier (Dr Louis Debute) had become the symbol of royalist cruelty to the rebelling colonies.

### New York ophthalmology, 1775-1801

**Figure 16.**
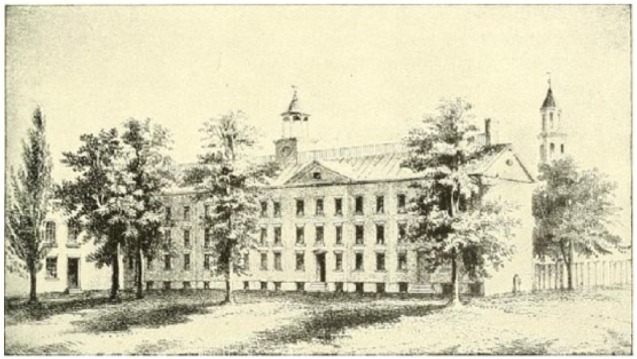
King’s College in New York in 1770.^352^

The notice establishing the medical school at King’s College, New York, in 1767 listed Samuel Clossey as the Professor of Anatomy and John Jones as the Professor of Surgery (Figure 16).^351,352^ In 1773, when James Graham delivered his lecture in New York on eye anatomy, physiology, and diseases, he invited “the Faculty.”^233^ Perhaps they attended because Clossey’s subsequent anatomy lecture of 1775 attributed cataracts to “the chrystalline humour” becoming opaque under the influence of solar radiation.^353^ Understanding the anatomy of the eye was essential to perform “the operation of couching.”^353^ He also discussed an operation to cure “obstruction of the lachrymal ducts.”^353^ The summary does not reveal whether Clossey actually performed these surgeries.^353^ Jones’ 1775 text, dedicated to his teacher Thomas Cadwallader, is considered the first American surgical text.^354^ However, this text does not describe eye surgery.^354^

The Hessian surgeon Friedrich Carl Pflug, who performed couching, advertised in New York in 1778. As mentioned above, New York was at least briefly a refuge of the loyalist Yeldall beginning in 1781 when he lectured on the eye.

Still, even in the loyalist bastion of New York, cataract surgery may have been difficult to obtain during the war. Hessian officer Wilhelm von Knyphausen, who had been instrumental in the British capture of Fort Washington, developed cataracts and requested dismissal in 1778 due to infirmities, including unilateral loss of vision.^355^ He left North America in 1782 when this request was granted.^355^

Another who sought travel to Europe due to cataract was New York attorney Peter van Schaack (1747-1832). In June 1778, at age 31 years, he wrote, “The disorder in my eye is now become so confirmed, as to exclude all hope of relief, but from the hand of an oculist.”^114^ He had “the continual apprehension of its communicating to the other eye” a prospect he found “more distressing to me than the terrors of immediate death.”^114^ He wanted to travel to England but was concerned others might attribute the request to loyalist sympathies.^114^ He formally requested of Governor Clinton of New York and was granted “permission to go to England, on account of a cataract in one of his eyes, and for the purpose of having an operation performed upon it by an oculist.”^114^ In London, in May 1779, he consulted with “Mr. Birch, surgeon” who performed “electrical operations” on his eye.^114^ In the fall of 1780, he developed “symptoms of a cataract in my left eye.”^114^ The symptoms consisted of “first motes and flitting clouds passing before the eye, and afterwards a dimness, which makes the atmosphere appear hazy, print considerably diminished, insomuch as to require a magnifying glass . . .”^114^ He returned from the country to London “to counteract so alarming an attack.”^114^ Birch resumed electrical treatments and placed a seton on the neck.^114^ He also saw Baron Michael Johann Baptist de Wenzel, who offered to perform an operation, but surgeon John Hunter dissuaded Van Schaack from surgery and agreed with the electrical and mercury therapies.^114^

**Figure 17.**
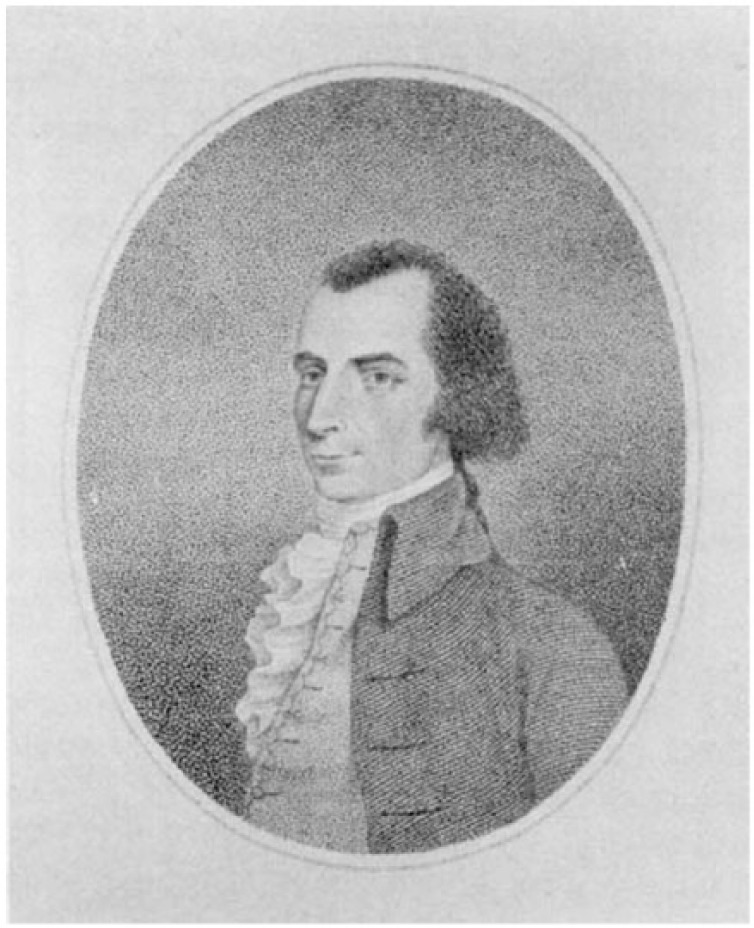
Charles McKnight (1750-1791) of New York.^284^

Ultimately, the development of ophthalmology at King’s College (later Columbia University) began under Charles McKnight (1750-1791) and Richard Bayley. McKnight began his medical training under Shippen and then directed hospitals during the Revolution, serving first under Church and then under Shippen (Figure 17).^284^ Returning to New York after the war, McKnight was appointed a professor of anatomy and surgery at the university in 1785. About the year 1788, McKnight trained William Stillwell, of New Jersey,^184^ who subsequently performed a successful eye surgery in Middletown in 1792.^163^ McKnight’s obituary called him “a Physician of very extensive practice . . . as a surgeon and oculist, perhaps unequalled in this country—this the many uncommonly skillful and difficult operations he performed in New-York, and in this city [Baltimore], strongly attest.”^160^

**Figure 18.**
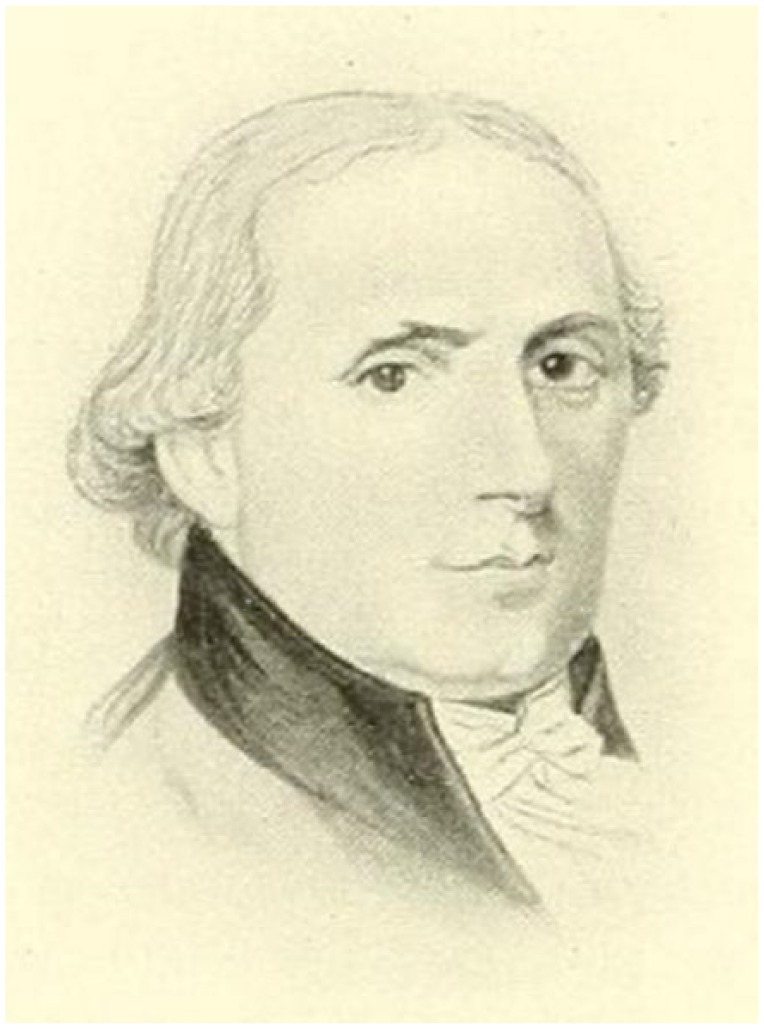
Richard Bayley (1745-1801) of New York.^352^

**Figure 19.**
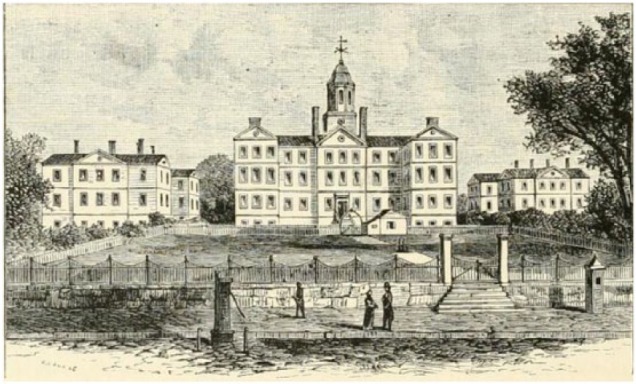
New York Hospital.^358^

Richard Bayley (1745-1801) of New York trained in London under “The Anatomist Dr. Hunter” from about 1769 until his return to New York in 1772 (Figure 18).^269,352^ Bayley performed an operation to cure John Lamb of a “hydrocele” or “watery rupture” (perhaps an inguinal hernia) in 1773.^356^ Bayley again studied in London during 1775, but in the Spring of 1776 returned as a surgeon in the English army under General William Howe.^269^ “Dr. Richard Bailey” attended the wounded after the Battle of Brooklyn in August 1776.^335^ In the fall of 1776, he served as the surgeon for the British in Newport, Rhode Island.^269^ He resigned his commission and returned to New York in the Spring of 1777 and was able to see his wife just before she died.^269^ Bayley began lecturing at the New York Hospital in 1786 (Figure 19).^357,358^ As a physician who remained in British-held New York throughout the war, Bayley’s wartime treatment of prisoners was questioned in the early post-war period, but he seems to have weathered the scrutiny. In 1788, the “Doctor’s mob” enraged by rumors of illicit procurement of the deceased destroyed his anatomical specimens.^269^ In the spring of 1792, Bayley was appointed a Professor at Columbia College.^269^

The letters of attorney Van Schaack indicate that Bayley had begun performing cataract surgery by 1783 when Van Schaack wrote to his brothers in New York: “. . . still am I halting between two minds about the operation. How kind would it have been, had you told me Colonel [Moses] Philips’s fate, and that of the others Mr. Bailey has operated upon.”^114^ The same year, Van Schaack wrote,

Still, the advice given me was against an operation, until it became absolutely inevitable by the total loss of sight. It may be said that the operation might be performed in America, and it was several times hinted to me, on account of the eminence of an operator at New-York; but when, after some difficulty, I obtained a history of the cases in which he had performed, I found no great room for confidence: indeed my own opinion of him never was very high.^114^

Van Schaack had still not had a cataract surgery by 1825 and perhaps never took that step.

Despite this sentiment, Bayley was generally well regarded. Bayley had a “general preference of extraction above depression of the lens in cataract.”^269^ We do not know when Bayley would have begun performing extractions (as opposed to couching). The handwritten diary from 1792 of medical student Jotham Post mentions many lectures and surgeries with Bayley without clarifying the subject matter.^359^ In 1793, Gabriel N Phillips, of Edenton, North Carolina, announced that “Having studied under the most eminent Professors in New-York,” he performed “different operations on the eye.”^178^ In 1793, in New York, the earliest identified advertisement in America for aphakic spectacles specified “James Rivington has . . . for the accommodation of persons, with couched & weak eyes . . . spectacles.”^164^ Post’s company in 1794 advertised couching instruments.^159^ In 1797, the New York Hospital treated 5 patients with ophthalmia and 2 with cataract, both of whom were cured.^360^ In August 1801, Bayley died after contracting yellow fever while inspecting ships in the Port of New York.^269^

The eye surgeries continued in his absence, though perhaps not with the same success rate. At the end of 1802, the hospital surgeons included Wright Post and Richard Kissam. Jotham Post was listed as the steward. That year, the hospital treated 7 patients with ophthalmia (1 of whom was “disorderly”), and 4 patients with cataracts, 1 of whom was “cured,” and 3 of whom were merely “relieved.”^361^

### University-trained ophthalmologists from Europe

As noted above, the first surgeon known to have performed cataract extraction in America was Frederick William Jericho of Germany. Jericho trained under Petrus Camper of Groningen and graduated from the University of Utrecht.^3^ His 1767 thesis was on cataract extraction and a case of orbital malignancy.^185^ Jericho appears to have imported the technique of cataract extraction to the New World between 1771 and 1776. With the outbreak of the revolution, Jericho returned to London, where he advertised in 1776 that he had just returned from the Caribbean. He performed not only cataract extraction but also treated lacrimal fistula. He also treated films on the eye and performed the earliest enucleation in the New World. Jericho was in England through 1777. He practiced in Bonn in 1780.^362^ Jericho returned to America, arriving in Philadelphia in October 1783, just 1 month after the Treaty of Paris. He demonstrated his technique of cataract extraction for both Thomas Bond in Philadelphia (who approved) and for physician Charles Wiesenthal of Baltimore (who implied that Jericho was a “quack”). Jericho practiced between Charleston and Boston, and then returned to the Caribbean, practicing in Jamaica and perhaps Santo Domingo. Jericho then returned to Europe.^3^

In 1782, Lewis Leprilete (1750-1804) of France settled in Providence, Rhode Island, and advertised cataract surgery, noting, “Cataract is an Operation by which the Eye is delivered of the crystalline Humour, become opacous.”^3^ Leprilete had moved to Norton, Massachusetts, by 1785,^363^ and by 1786, he had “performed the Operation for the Cataract on a Person at Warren with Success.”^364^ In 1791, Leprilete purchased the Hallowell House in Jamaica Plain,^365^ which was one of the wartime hospitals supervised by Church. That year, Leprilete was the first American author to publicize Franklin’s invention of bifocals.^3^

**Figure 20.**
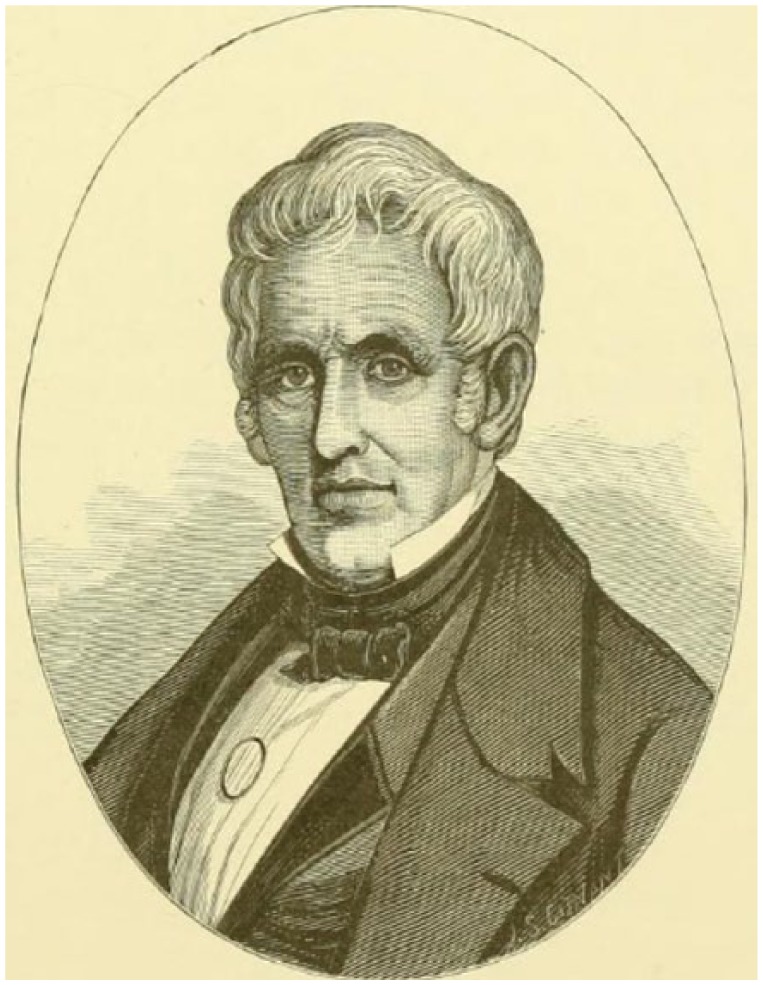
Nathaniel Miller of Massachusetts (1771-1850).^367^

Leprilete trained numerous medical students, including Nathaniel Miller (1771-1850), from 1790 to 1792 (Figure 20).^366,367^ Leprilete probably taught Miller how to perform cataract extractions, which Miller preferred to depression.^368^ In 1792, Miller went into practice in Franklin. “Within the first eight weeks, he performed successfully two capital operations;—one for cataract by extraction, upon a patient from Long Island.”^366^ He then went to Guadaloupe,^366^ or perhaps Santo Domingo, for a few months, but “found little sympathy and even less employment.”^368^ He returned to Franklin where

His operations for hernia and cataract were quite numerous . . . In May and June, 1798, he operated upon twenty-one eyes by extraction: all recovered sight, save one, in from three to six weeks. Artificial dilatation of the pupil was not then in use. In operating, he used either hand with equal facility, and always adhered to the practice of not operating while only one eye was diseased.^366^

**Figure 21.**
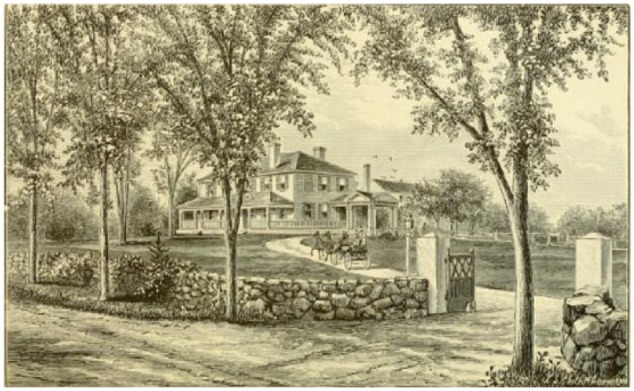
Hospital in Franklin, Massachusetts established by Nathaniel Miller in 1816.^367^

Leprilete returned to France for about 7 years in the 1790s. He had been in Guadeloupe before returning to Franklin in 1801. Leprilete practiced with Miller until his death in 1804. In his will, Leprilete called Miller “my beloved and adopted son.”^369^ Leprilete’s body was donated to John Warren for dissection.^3^ Miller established a hospital in Franklin in 1816 (Figure 21).^367^

George Kiesselbach landed in Charleston in 1790, where he performed cataract operations, and from 1795 to 1798 proceeded through Norfolk, Boston, Newport, New York, and Philadelphia.^370–374^ He trained with many oculists in Europe and had studied at the Universities of Gottingen and Marburg.^371^ We suspect that he performed extraction, rather than the standard couching of the period because he wrote,

Should any patients present themselves with Cataracts on their Eyes, who cannot be cured but by the operation (which he performs in a much lighter and more improved manner than heretofore practiced) they shall be at liberty to bring any Doctor with them, in whose presence he will perform his operations.^374^

It is doubtful other doctors would be interested in witnessing the standard couching procedure.

### Mid-Atlantic oculists from France, 1780-1800

The “French Physician, Surgeon, and Botanist” Joseph Goss studied in Paris and Montpellier and was “sworn in the society of the Doctors of Nismes in France.”^375^ He began practicing in the 1760s^376,377^ and arrived in Philadelphia in about 1780.^376^ Perhaps he was influenced by the success of Jericho’s visit because in 1785 (and only that year), he advertised as an eye surgeon:

. . . said Doctor will restore the sight to any person unhappily afflicted by blindness from a cataract on the eye, performed by a delicate operation. He has a marvelous eye-water of his own composition to cure or prevent cold in sore eyes.^152^

Goss might not have been satisfied with his outcomes because in subsequent years, he failed to mention eye surgery.^377^

The Haitian Revolution, which began in 1791, resulted in French physicians leaving the island of Saint-Domingue (Santo Domingo). “Mr. Raymond Frederick, Surgeon . . . Oculist” had studied in Paris, Montpellier, Madrid, and Rome, and then fled from Santo Domingo “on account of the sad troubles which now prevail in those parts” to New York in 1792.^378^ He was one of only a handful who advertised artificial eyes.^378^ Later that year, he moved to Baltimore, but strangely, he reversed the order of his name:

Mr. Frederick Raymond . . . lately arrived here from the Island of St. Domingo . . . As an Oculist . . . after a study of thirteen years, in the principal academies in Europe . . . He treats all disorders of the eyes . . . and puts in artificial eyes resembling the natural, and which give no pain nor lay the patients under any constraint.^120^

A set of his “Dentist, Oculist, and Physician’s Instruments,” containing 63 “silver mounted” pieces went missing^379^ but was later returned.^380^

Jean Devèze (1753-1829) trained in France and moved from Santo Domingo to Philadelphia in 1793.^381^ In 1795, his patient

. . . lost the use of one eye by a cataract . . . another pellicle growing over his second eye deprived him entirely of his sight . . . thanks to the care and skill of M. Deveze, he was operated in presence of several Physicians and Surgeons with all possible dispatch and success.^382^

A yellow fever epidemic broke out in Philadelphia in 1793. During the epidemic, Devèze treated patients at the hospital at Bush Hill.^383^ He wrote a treatise describing his cases, in which he advocated moderate bleeding, blistering, wine, and decoction of bark.^381^ The disease spread through “miasmata” retained in the air.^381^ By 1821, Devèze had returned to Paris, where he declared that yellow fever was not contagious, and quarantines were unnecessary.^384^

Goss treated 60 patients during the 1793 epidemic.^385^ He recommended 12 hours of sweating or a hot bath and consumption of ditiny tea and a decoction of turnips, endive, and carrots.^385^ This decoction could also be used as a clyster (enema)^385^ Vomiting could be suppressed with “ippecacuana.”^385^ Goss also reviewed the history of burning fires outdoors to eradicate the epidemic and offered a “perfume” which could prevent the disease.^386^

### Southern eye surgeons, 1785-1800

**Figure 22.**
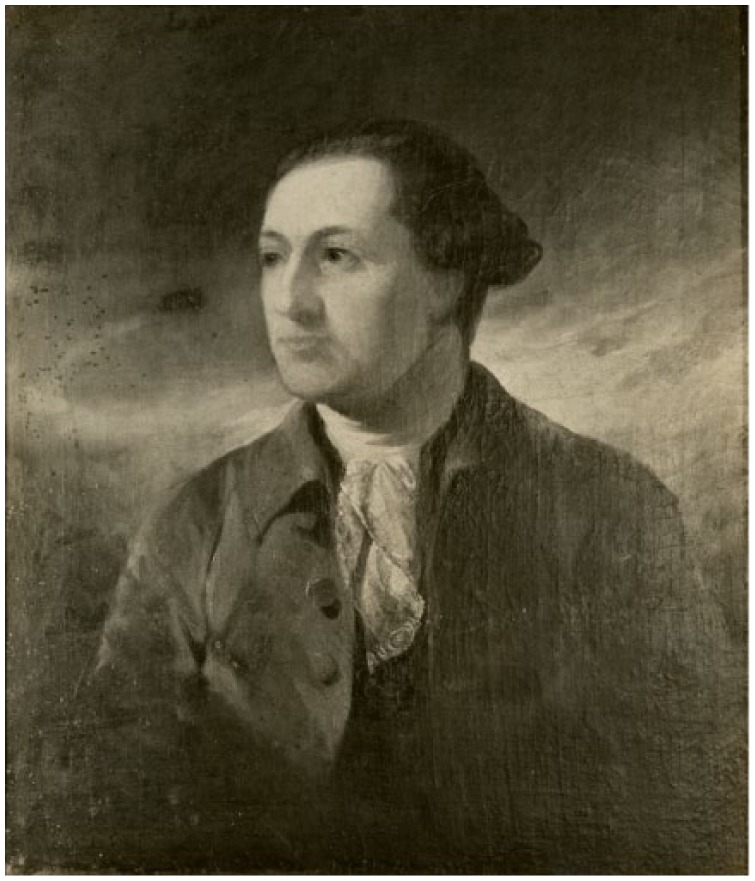
William Baynham (1749-1814) of Essex, Virginia. Courtesy of the Virginia Historical Society.

Surgeon William Baynham (1749-1814) of Virginia, studied at St. Thomas Hospital in London beginning in 1769 and returned to Essex County in 1785 (Figure 22). Baynham performed “operations for . . . cataract”^387^ and was friends with George Washington. In July 1799, Washington asked Baynham to treat a 28-year-old servant:

my Ploughman, has, for (some months) past, been afflicted with a tumour which has occasioned partial, and threatens . . . total blindness. He has been under the care of Doctor [James] Craik . . . without receiving much, if any benefit.^388^

Baynham replied, “I had operated on your Servant Tom’s Eyes . . . The tumor in the left Eye is . . . incurable; and a growing film in the right threatens to overspread the transparent Cornea.”^92^ Given the lack of surgical success, Baynham offered a discount:

In the operations which I performed, as I was prompted rather by a wish than an expectation of relieving the poor fellow, I hope you will not take it amiss if I claim no more than a consultation fee of five dollars.^92^

In 1786, John Tyler of Frederick, Maryland, returned from training in London and Edinburgh and advertised that he had seen “extracting or depressing the cataract.”^389^ Within a few months, he had performed eye operations on at least 3 patients.^390^ Subsequent biographies suggest that he settled on couching as his procedure of choice.^391^

Tyler was an elector who supported Thomas Jefferson in the presidential election of 1800. In October 1801, Tyler described a complex ophthalmic case in a letter to President Jefferson:

At the request of [Jefferson’s physician] Doctor [Edward] Gantt of George Town I have examined the eyes of a young man, said now to be in your service . . . it would be improper to attempt the operation for the removal of the Cataract at this time . . . there is a partial paralysis of the Optic nerves in both eyes, an entire opacity in the Chrystalline lens of the right eye, a small opacity in the lens of the other, that the nervous affection is the primary disease, and that when the Cataract is removed, vision will be very imperfect until the energy of the nerves can be fully restored.^392^

Tyler recommended deferring

the operation until the next spring, when the Cataract will probably acquire a firmer consistence more favourable to its removal and when the ensuing warm season will enable us to pursue an alternative mercurial course for the removal of the paralytic affection . . . .^392^

The differential diagnosis of a bilateral optic neuropathy in a young adult with bilateral, but asymmetric, cataracts is long and includes uveitis.

This patient was probably John Christopher Süverman, whom Gantt treated medically in March 1802, when the medical bill indicated “Christopher lotion.”^393^ An associate noted in May,

Christoph is at my house. The doctor began a major treatment that he hopes will destroy the thing that is affecting his better eye, the one without the cataract. In two weeks the doctor will know if the unfortunate man can recover his sight. If he cannot see in two weeks with his better eye, the operation would be useless, since after removing the cataract, the same cause that affects the better eye would still exist in the other one, which does not see at all. If the treatment does not succeed, this poor unfortunate will have to go to the hospital or poor house.^394^

The conservative treatment was unsuccessful. In 1810, Jefferson wrote that Süverman has “become blind, and gets his living by keeping a few groceries which he buys & sells from hand to mouth.”^395^

Joseph Brevitt (1769-1839) trained in London and then practiced on Guadeloupe (in the British Army) and Antigua from 1796 to 1797.^396,397^ On his arrival in Baltimore in 1798, he advertised that he “extracts the cataract and treats every disease of the eyes.”^396^ In 1799, Brevitt published a very brief account of the history of anatomy.^398^ Brevitt’s treatise of 1810 noted that “Opthalmia, or sore eyes” in young children could be treated with “leeches applied to the eyelids or temples . . . setons or issues applied to the back of the neck . . .”^399^ Newborns might be born with the eyelids “grown together,” but the connection can be “put upon the stretch and carefully cut through.”^399^

### Ophthalmology at Harvard, 1783-1801

**Figure 23.**
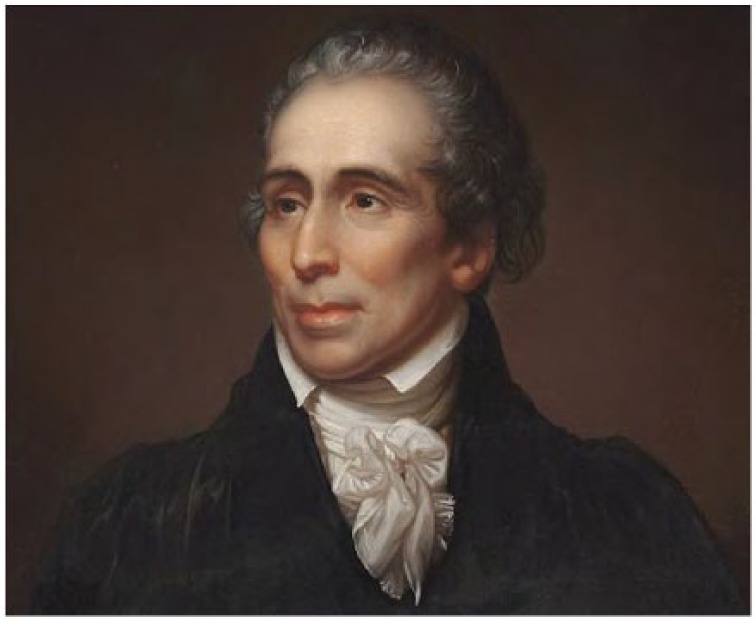
John Warren (1753-1815) by Rembrandt Peale.^400^

In the fall of 1782, the Harvard Medical School was established in Cambridge. John Warren was appointed Chair of Anatomy and Surgery (Figure 23).^400^ One can see a dramatic progression in ophthalmology at Harvard over the course of Warren’s career. Initially, Warren’s ophthalmic understanding was quite limited. In his anatomy lecture notes of 1783 to 1784, Warren relayed a story originally told by Robert Boyle about a man confined in a dark dungeon in Madrid for several weeks who eventually was able to perceive the mice eating crumbs on the floor but when freed could not initially tolerate daylight.^401,402^

Warren also lectured that “the first defect in vision is in seeing subjects inverted.”^401^ Indeed, because the retinal image is inverted, some scholars assumed that “all Objects, to a Person who is first brought from Blindness to Sight, do appear inverted.”^403^ This idea was (incorrectly) believed to have received empirical support in 1751 in England when an 8-year-old boy with congenital cataracts was couched and “having a Pin held up before him . . . being bid to touch the Head, he laid his Finger upon the Point of it.”^403^

Warren lectured about the eye anatomy of birds.^401^ He also recounted his sensations during his first moments of life. These passages were intended to be interpreted literally, rather than a guess about what a baby might experience. Warren wrote,

I well remember that joyful . . . moment when I first became acquainted with my own existence . . . I opened my eyes, what an addition to my surprise! The light of the day, the azure vault of heaven . . . .^401^

These sights “filled me with incomprehensible delight.”^401^ Then, “I turned my eyes to the sun. Its splendor dazzled and overpowered me.”^401^ Six pages recount his first experience of each sense: vision, hearing, touch, smell, and taste.^401^ He concluded that touch was more reliable than vision.

It appears that Warren accepted some of the more fanciful European speculation about vision and was willing to indulge in philosophical musings of his own. However, these early notes provide no indication that he had begun performing cataract or other eye surgery.^401^

Warren’s interest in eye surgery might have been stimulated by the arrival of both Jericho and Leprilete to Massachusetts in 1785. In 1788, George H Hall, 1 of 2 students in the first class of “the University of Cambridge” to receive “the degree of Bachelor of Physick” read his “Dissertation on the Cataract” which discussed its history and “its cause and cure.”^404^ John Adams attended the graduation ceremonies held in “Harvard Hall.”^404^

The teaching of couching at Harvard in the late 1780s is also suggested by the story of surgeon Nathan Smith, who trained with Warren at Harvard from 1789 to 1790.^405^ After Smith went into practice, he requested couching instruments of Warren in February 1791, though nothing came of the request at the time.^405^ In 1794, Warren lectured on lacrimal fistula and on both cataract couching and extraction.^105^ Warren preferred couching, with which he had better results perhaps because he did not perform a capsulorhexis along with extraction.^105^

Some time before 1806, Warren “had operated for cataract with perfect success” on the eyes of the son of a Mr Gilpin of Newport.^406^ This gentleman might be the diplomat who arrived in 1803.^407^

His son John Collins Warren, who returned from training in Europe in 1802, preferred extraction to couching or division (dividing the lens into pieces which remained in the eye).^97^ The elder Warren was undoubtedly influenced by his son, and in 1806 recommended cataract extraction, and assisted his son in the operation, for angle closure glaucoma (see above). Thus, over the career of John Warren, Harvard Medical School progressed from teaching almost no practical ophthalmology to being at the forefront of the field.

### Nathan Smith in New England in 1798

**Figure 24.**
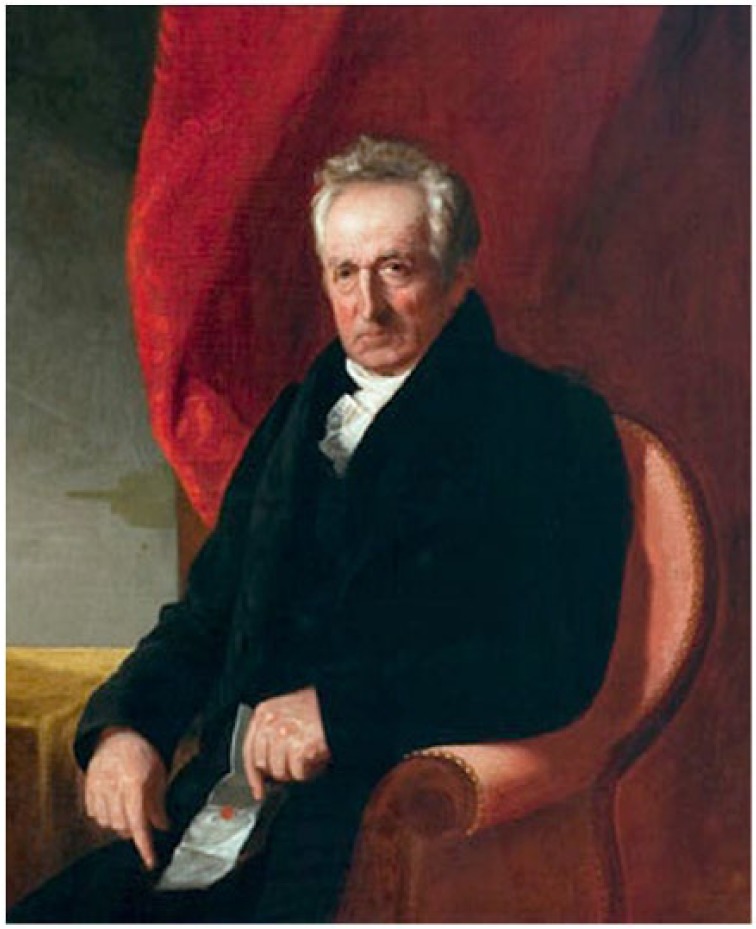
Nathan Smith (1762-1829) of New Hampshire, painted by Samuel Morse.^408^

After an apprenticeship with Josiah Goodhue in Putney, Vermont, Nathan Smith (1762-1829) began practicing medicine in Cornish, New Hampshire, in 1787 (Figure 24).^408^ After his training at Harvard Medical School in 1789 and 1790, where he earned a Bachelor of Medicine degree, Smith returned to Cornish.^405^ He attempted twice in 1791 to obtain couching instruments from John Warren of Harvard.^405^ Smith spent the first half of 1797 observing medical lectures and surgeries in Edinburgh, Glasgow, and London.^405^ Later in 1797, he began lecturing students in Hanover.^405^ His efforts lead to the founding of the Dartmouth Medical School and his appointment as a professor in 1798.^405^ Perhaps, his journey abroad or his role as a medical educator reinvigorated his interest in eye surgery. In June 1798, he paid 12½¢ to his neighbor, a silversmith named Jedediah Baldwin, for “setting a couching needle.”^405^ His accounts for 1800 included charges for “extracting a tumor near an eye” ($5) and “extracting an eye” ($22) but no cataract surgeries.^405^

**Figure 25.**
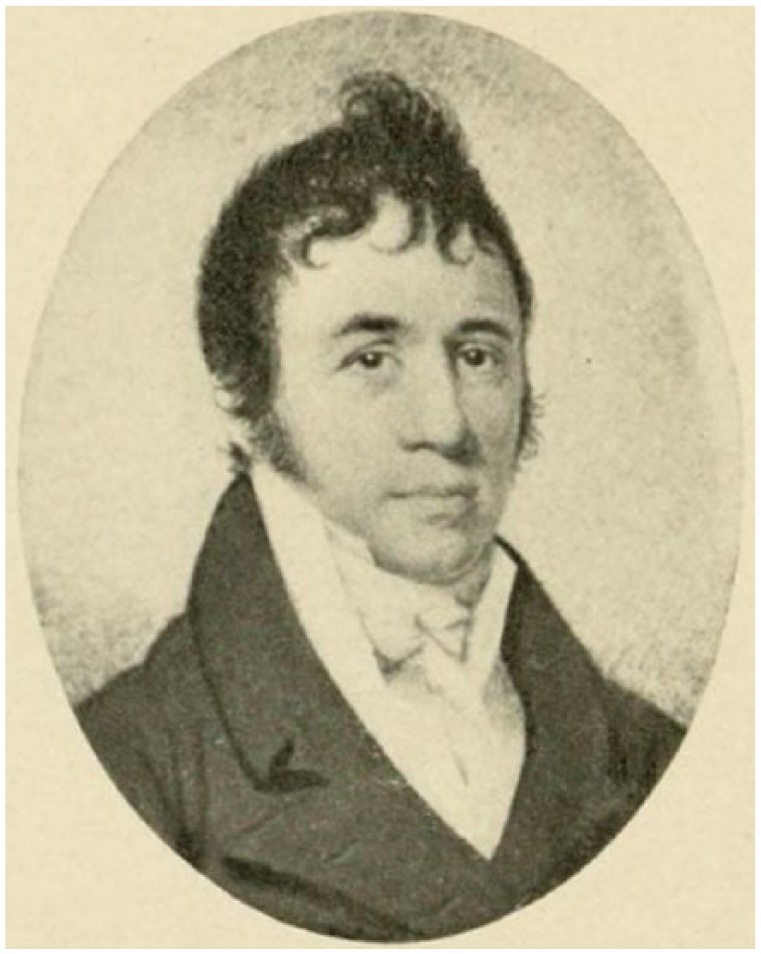
Lyman Spalding (1775-1821) of New Hampshire.^409^

Smith had an apprentice named Lyman Spalding (1775-1821) who subsequently began training at Harvard Medical School in 1794^409^ and covered Smith’s practice during his 1797 voyage (Figure 25).^410^ Spalding set up his own practice in Portsmouth, New Hampshire, later in 1797.^408^

From antiquity, surgical texts had recommended ambidexterity so that the operator could approach the sitting patient’s eye temporally while facing the patient. The right hand operated on the left eye and vice versa. An alternate approach for the right-handed surgeon operating on the right eye was to stand behind the patient. Smith tried that approach. In September 1807, he wrote his colleague Spalding that only once had he (Smith) attempted to extract the cataract of a right eye:

Respecting extracting the cataract on the right eye, I have performed once only on that eye. I stood behind the patient and introduced the knife in the usual manner excepting the edge was turned in an opposite direction, so as to cut the flap upward, which is preferable to cutting it downward, as the cicatrix is apt to produce some obstruction to vision in looking down on the ground . . . .^410^

By December of that year, Spalding himself had begun cataract surgeries, although he performed couching, as a fellow doctor wrote, “I sincerely congratulate you on your success in couching.”^410^

Smith operated on the cataract of a Mr Darby of Boston in October of 1809.^411^ In February of 1810, Smith asked Spalding, who was studying in Philadelphia, to “make diligent inquiry of Dr. [Philip Syng] Physic[k] respecting his mode of operating on the eyes & what kind of instrument he uses.”^405^ One of Smith’s students recorded in November of 1810 that

Doct. Smith has performed the operation of Couching five times within these Six weeks. They report to him from all parts of the Country, one person from the vicinity of Boston Came here Completely blind and had both Eyes operated upon about three weeks since, She can now see to read tolerably well by the assistance of Glasses.^405^

Smith subsequently couched many cataracts.^405^ Therefore, it seems that although he tried extraction, he settled on couching as his procedure of choice.

Smith later began to teach surgery at several newly formed medical schools: at Yale in 1813, and, for a brief period, at the Maine Medical School in Brunswick in 1821.^405^ Notes from his lectures at Yale for 1824 and 1825 addressed removal of eyes with cancer, ptosis operations, eyelid tumors, “Coagulated Substances on the Cornea,” and “Dropsy of the Eye,” defined as vision loss from “a collection of fluid within its coats.”^405^ In 1826, a patient consulted him regarding “a prolapsus of the iris.”^405^

### University of Pennsylvania, after 1779

A medical student from Maryland noted that to “cure ophthalmia” associated with inoculation for smallpox, one should bathe the eyes with cold water, sometimes with the addition of “saturnine [lead] preparations.”^412^ This remedy he had learned from his professor, Benjamin Rush (1746-1813), professor of medicine.^413^ Another of Rush’s students recommended blistering the temples for “ophthalmia” and the top of the head for “amaurosis.”^414^ Blistering for ophthalmia could be accomplished with a type of cantharis beetle which was discovered in the United States.^415^

**Figure 26.**
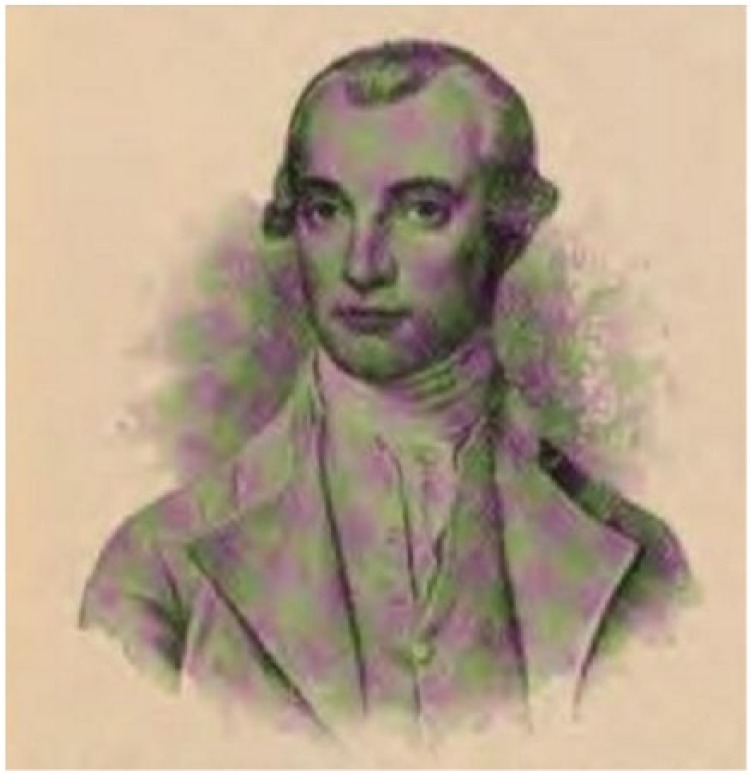
John Foulke (1757-1796) of Philadelphia.^201^

The era of couching had come to the Pennsylvania Hospital by the 1790s and perhaps earlier. As noted above, William Shippen Jr had received couching instruments in 1779.^150^ Another physician, John Foulke (1757-1796), studied toward a medical degree at the College of Philadelphia in 1780 (Figure 26).^201^ Foulke studied medicine in Holland, Germany, and France from 1780 to 1783 and was a Lecturer on Anatomy from 1784 to 1796 at the newly named University of the State of Pennsylvania (which eventually was named the University of Pennsylvania). He also practiced at the Pennsylvania Hospital from 1784 to 1794.^201^ Foulke’s estate listed couching instruments.^151^

**Figure 27.**
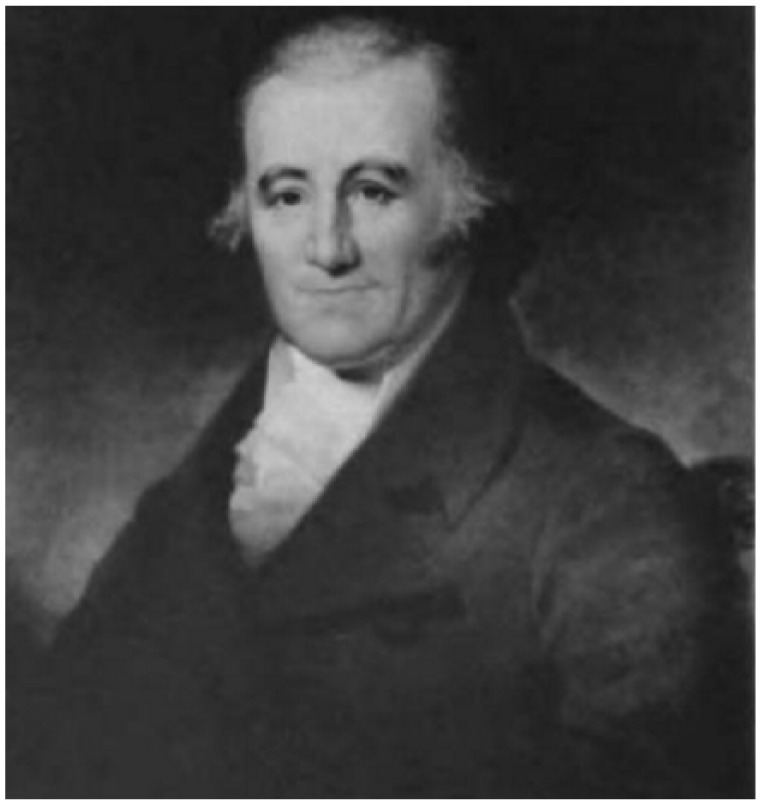
Portrait of Caspar Wistar (1761-1818) of Philadelphia.^416^

Caspar Wistar (1761-1818) was a physician and botanist born in Philadelphia (Figure 27).^416,417^ He began his medical training locally, under John Morgan, William Shippen Jr, and Benjamin Rush. Wistar received a medical degree at Edinburgh University, after studying under William Cullen. Wistar’s thesis of 1786 was dedicated to Benjamin Franklin.^418^ On his return to Philadelphia, he practiced with John Jones (1729-1791). Wistar was the Professor of Chemistry and Physiology in the College of Philadelphia after 1789, a physician at the Pennsylvania Hospital from 1793 to 1810, and Professor of Anatomy in the Medical School of the University of Pennsylvania after 1808.^416^

In 1793, Wistar couched the cataract of the 71-year-old botanist Humphrey Marshall.^416^ The surgery was a “partial success, for the old man was at least enabled to distinguish his favorite plants as he walked in his garden.”^416^ In 1815, Wistar gave his set of eye instruments to his trainee Charles Wilkins Short when the latter returned to his native Kentucky.^416^

Wistar published a text on anatomy and described the anatomy of the ethmoid bone.^416^ The flowering vine Wisteria is generally believed to be named after Wistar, to acknowledge his botanical work.^416^

Perhaps Shippen, Foulke, or Wistar taught Bildad Beech, who studied first with his brother Elnathan Beech and then studied medicine and surgery at the hospital in Philadelphia.^419^ In his 3 years of practice in Whitestown, New York, Bildad Beech performed “Trepaning, Amputation, the Operation for the Hernia, Couching, . . .”^419^ But “finding the Western Country unfavorable to his health,” he settled in Cheshire, Connecticut, in 1795.^419^

Another student of the period who might have been exposed to eye surgery at the university was Elisha North (1771-1843). North attended the University of Pennsylvania in 1793 and 1794.^420^ North’s illustrations show that he performed couching.^420^ However, we have no evidence that North performed couching until his 1814 advertisement in New London of the “operation for a cataract.”^420^ North is credited with opening the first eye infirmary in the United States in New London in 1817.^420^ He also presented an eye speculum of his own invention in 1821.^420^

**Figure 28.**
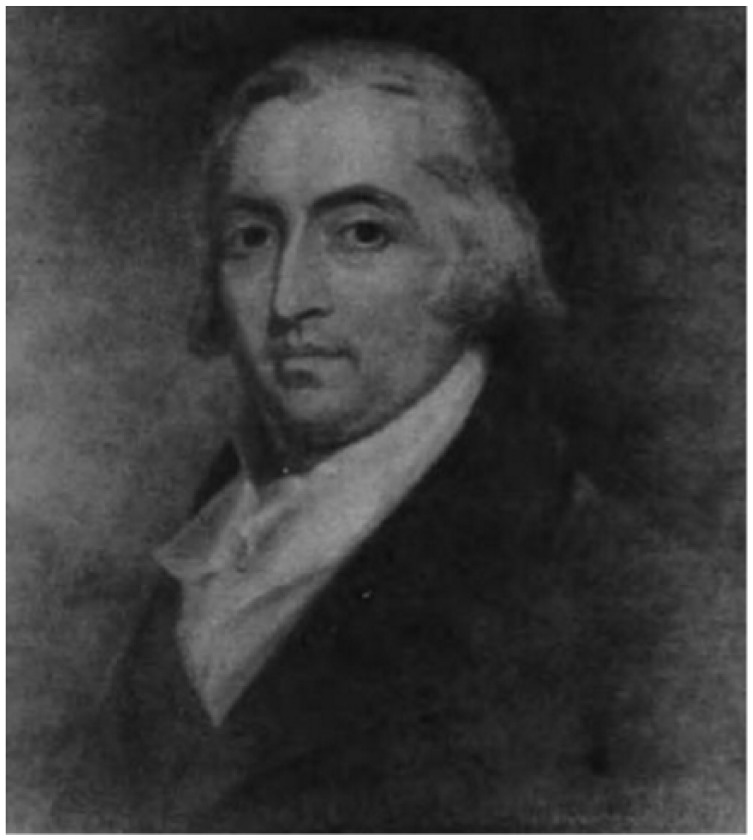
Philip Syng Physick (1768-1837) of Philadelphia.^416^

In Philadelphia, the era of cataract extraction began in earnest with the practice of Philip Syng Physick (1768-1837; Figure 28).^417^ A native of Philadelphia, Physick first studied medicine under Adam Kuhn at the University of Pennsylvania.^98^ In 1789, Physick traveled to London to study under John Hunter and was a house surgeon at St. George’s Hospital.^98^ He then trained at Edinburgh and in 1792 received the Doctor of Medicine degree.^98^ Physick then returned to Philadelphia. He was appointed to the Pennsylvania Hospital in 1794, the Medical Department of the University of Pennsylvania in 1800, and became the Professor of Surgery there in 1805.^98^ His surgical journal begins in 1795, and the first recorded case is of a woman whose vision he restored with cataract surgery.^98^

Over the course of Physick’s tenure, University of Pennsylvania graduates would carry eye surgery techniques throughout the new republic. Among the earliest was Joseph Glover who graduated in 1800 and settled in Charleston in 1801. His biographer wrote that Glover performed eye surgery from the beginning of his career and “monopolized” eye surgeries in that area, attracting business even from the neighboring states.^421^ His biographer wrote, “For cataracts, he operated either by extracting, or by depressing the lens.” Glover operated even on “‘very elderly’ individuals, i.e. 65, 70, and 83 years.”^421^

John Syng Dorsey (1783-1818), Physick’s nephew, studied medicine with his uncle from 1798 to 1802 when he graduated from the University of Pennsylvania.^422^ Dorsey then studied medicine at St. George’s Hospital in London, and in Paris, and returned to Philadelphia in 1804.^422^ His surgical text of 1813 combines his observations with those of Physick and European authors.^81^ Dorsey reviewed surgery for ectropium, entropium, pterygium, lacrimal fistula, artificial pupil (iridectomy), anterior chamber paracentesis, couching, cataract extraction, congenital cataract, and extirpation (enucleation) of the eye.^81^

### Mason Fitch Cogswell, 1793

Mason Fitch Cogswell (1761-1830) studied medicine with his older brother, James Cogswell, in Stamford and New York,^421^ before practicing on his own in Hartford in 1789.^421^ The younger Cogswell might have become interested in ocular health because he had problems with his own eyes. In March 1787, his friend Mary Ann Moore wrote to him in New York (apparently before he had begun medical training), “Poor blind creature. I sincerely pity you for I have experienced the pain of weak eyes can feel for you. Will you allow me to prescribe for you an eye water that never fails of curing mine.”^423^ In September of that year, she wrote again, “I may conclude that you have commenced physician . . . I had imputed your not writeing to the weakness of your eyes.”^424^

**Figure 29.**
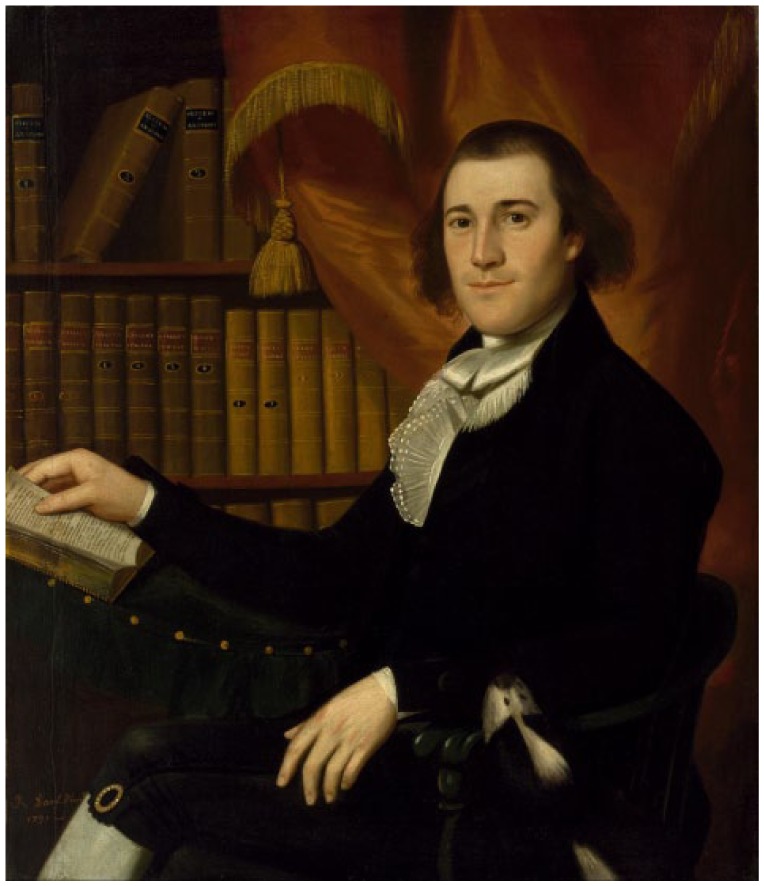
Portrait of Dr Mason Fitch Cogswell, painted by Ralph Earl in 1791.^425^

Cogswell showed an early interest in surgery of the eyes (Figure 29).^425^ In 1793, a woman from Brooklyn wrote to request “the removal of a film or an opaque tumor from the eyes.”^90^ Also, in 1793, surgeon Eldad Lewis of Lenox, Massachusetts, wrote to Cogswell about a 60-year-old man with white cataracts bilaterally.^426^ After Lewis performed phlebotomy, “I operated upon the right eye, as being of the longest standing, & succeeded perfectly in depressing the Cataract, as low as the inferior margin of the pupil.”^426^ Milky fluid burst from the lens and the patient had postoperative inflammation.^426^ Lewis asked Cogswell whether he should operate on the same eye again, operate on the other eye, or just observe.^426^ This letter suggests that Cogswell was viewed as a regional authority on couching very early in his career.

At some point, Cogswell began to perform cataract surgery by extraction.^421^ In December 1801, Cogswell wrote to Elias Graves, a resident of Guilford, about a cataract surgery: “I am truly sorry for the failure of the operation.”^427^ With the postoperative care of Doctor [Eli] Todd, Cogswell wrote, “you had every advantage for the recovery of your eye.”^427^ Before the surgery, it had appeared to be “a simple cataract,” but in retrospect, there had probably been “disorders in the humours of the eye.”^427^ Cogswell proposed a reduced payment of $20, but wrote that if Graves still thought the price too high, he should send what he could afford.^427^

In 1803, a surgeon who performed extraction, Charles F Bartlett, wrote that cataract extraction was not performed in Hartford or listed on Cogswell’s fee schedule, which Bartlett had obtained from Cogswell.^428^ This statement suggests that Cogswell did not perform extractions before 1803.^3^ Perhaps Cogswell learned of the procedure from Bartlett.

Cogswell’s description of an 1807 epidemic of “spotted fever,” known today as cerebrospinal meningitis,^90^ listed ophthalmic implications of the disease.^429^ Patients had eye inflammation and the pupils could be either dilated or constricted. Cogswell thought bleeding was used excessively.^429^ Cogswell’s daughter Alice was infected during this epidemic and lost her hearing.^90^ Cogswell noted the lack of training for the deaf in this country and petitioned the legislature for funding which permitted Thomas Hopkins Gallaudet to travel to Europe to study the educational methods there. After Gallaudet returned, the Connecticut Asylum for the Education of the Deaf and Dumb was founded in 1817.^421,430^

The announcement of the opening of the Hartford Dispensary and Surgical Infirmary in 1830 with Cogswell as a consulting surgeon noted, “Diseases of the eye and ear will receive particular attention.”^431^ Richard Kissam trained with Cogswell 1827. Kissam wrote a thesis on iritis at the College of Physicians and Surgeons in New York in 1830 and performed the world’s first corneal transplant with a human recipient in 1838.

### Charles F Bartlett, 1801

Charles Frederick Bartlett (1766-1806), the son of John and Lucretia Bartlett, was born in Westerly, Rhode Island.^432^ As noted above, Charles claimed to have “first commenced his medical career” when he accompanied his father during the revolution at the age of 11 years “and was present at the capture of General Burgoine [John Burgoyne] and his army” after the second Battle of Saratoga.^281^ It is conceivable that an 11-year-old could accompany and assist a surgeon father in war; however, no evidence confirms the statement. Charles Bartlett received additional medical training at the Hotel Dieu in Paris in the early 1780s.^281^ Bartlett landed in Bermuda in 1785 and established a surgical practice there.^433^ Bartlett had performed “Lithotomy, or cutting for the Stone in the Bladder” first while at the Hotel Dieu and several times subsequently in the West Indies.^93^ He married a local woman in 1786.^434^

In 1787, his physician half-brother John Bartlett, Jr (born 1755),^265^ settled on Turks Island and wrote that he “professes the Cure of the Cancer, in a late discovered method.”^435^ This cure must have been a family secret because in 1790, when Charles moved to Charleston, South Carolina, he advertised “Dr. Bartlett also professes the secret art of curing Cancers . . .” and announced the establishment of a hospital for “sick negroes.”^436^ In June 1790, “Dr. C. Fredck Bartlett” asked the Medical Society of South Carolina to admit him, although it appears this request was rejected.^191^ In June 1792, Bartlett performed the autopsy on the infant of “the celebrated miniature painter” Peter (Pierre) Henri of France. Bartlett diagnosed “a perforation of the gall bladder.”^437^ The fee schedule signed in Charleston in July 1792 by Charles F Bartlett, Nathan Brownson (whose subsequent estate sale listed couching instruments^179^) and other physicians included no eye operations.^438^ Prices depended on whether the patient was a “white person” or “a slave,” the weather, whether it was dark, and “for [the physician] rising out of bed.”^438^ Not until 1804 were “The Operation for the Cataract” ($10) and for “Fistula Lachrymalis” ($10) added to the fee schedule in Charleston.^191^ Later that year, he was declared insolvent, and notice of the sale of his estate was posted by the sheriff. The estate included “negroes,” a horse, furniture, books, and medicines.^439^

Bartlett returned to Bermuda in January 1793 and announced the resumption of his medical practice.^440^ Always ambitious, he announced in March of 1793 that he would open a “large and commodious hospital” for smallpox inoculation.^441^

Next, Bartlett decided to change careers. He no longer needed his medicines and advertised their sale in June of 1793.^442^ Working with his wife’s family as a privateer was possible because the January 1793 execution of Louis XVI of France had led to war between Britain and France.

In the waters near Port-au-Prince, the brigantine *States General* of Charleston, captained by Peter Dardelie, was captured by the privateer schooner *Kate* captained by Joseph Brownslow of the firm Jennings and Brownslow and was taken to St. George, Bermuda, arriving in July 1793. After landing, “a certain doctor Charles Frederick Bartlett . . . came on board the brigantine . . . in the character of agent.”^443^ Bartlett asked Dardelie the time. When Dardelie took out his gold watch, Bartlett took it, saying “if the brigantine . . . was a good prize, the watch would be so likewise.”^443^ The Bermudans confiscated the wine.^443^ In the fall, Bartlett had wine for sale.^444^

In October, he announced that he would command “The beautiful Sloop *Bermudiana*, a 14 Gun Privateer.”^445^ He set out in early November.^446^ Although Bartlett claimed to be a lawful privateer, the American newspapers labeled Bartlett’s actions “Piracy.”^447^ On December 3, 1793, the *Bermudiana* under Bartlett plundered the ship *Eliza*, which was stranded on Philips Reef, off East Caicos.^448^ The Bermudians confined the ship’s officers, and several of the *Eliza*’s crew ultimately died on Turk’s Island.^448^ The *Eliza*’s crew made a vow “for bringing the perpetrators of such barbarity to punishment.”^448^

The *Bermudiana* also captured the ships *Swallow* and *Sally*.^449,450^ The schooner *Mercy* set out from Charleston and was ultimately seized by Bartlett and his privateers in Jean-Rabel (Haiti) in early 1794. On reviewing the ship’s documents, Bartlett declared that he knew the owners to be “damned rascals” from his time in Charleston.^447^ Bartlett took the ship first to Turk’s Island^447^ and then to Bermuda.^451^ The South Carolinians reported, “This pirate is the same Doctor Bartlett, who defrauded so many people in this city of their property . . . The owners of the privateer are Jennings, Tucker & Co. of Bermuda, and possess large property in Charleston.”^447^

The news of his switch to privateering traveled quickly to America via the Tucker family, natives of Bermuda. St. George Tucker of Williamsburg described Bartlett’s career switch in a letter on March 1, 1794: “Dr. Bartlett, the spermaceti doctor . . . has turned privateersman, and commands a vessel out of Bermuda.”^452^ Spermaceti was not listed among Bartlett’s medical supplies, although he did sell vitriol, ether, ginseng, opium, and digitalis.^93^ Spermaceti candles were sold by a Bermuda store with which he was affiliated.

Bartlett’s wife gave birth to a son in March 1794, with the baptism in May.^453^ Shortly thereafter, his ship left for Tortola under another captain. Bartlett established himself as a physician on Tortola. There, he treated a “gentleman” named Charles Wills Walrond who died of yellow fever in January 1795.^454–456^ In July 1796, he treated soldiers of the 88th British regiment who carried an epidemic of yellow fever from Grenada in the ship *Betsy transport*.^454,457^ One soldier seemed to be doing better, but on hearing that his uncle had died, “immediately renounced ideas of life, and expired in a few hours.^454^

But by 1798, on Tortola, Bartlett had reverted to his career as a privateer. In September 1798, even those who noted “Bartlett’s Character was notorious as a rapacious privateer owner” still referred to him as “Dr. Bartlett.”^458^ Privateering was considered socially acceptable by some. In October 1798, Bartlett met an American officer at a tavern and procured a horse for him and brought him to the President of the Island. The officer noted, “Doctor Bartlett appears to be a Man of Confidence with the president of the Island, and to all appearance a Man [of] Respectability.”^458^ In 1798, a Danish brig was seized by about 25 sailors on the *Little Arch*, a privateering schooner owned by Bartlett and taken to Tortola.^459^ The owner of the Danish cargo asked Bartlett for permission to board the brig but was denied. The owner went to Bartlett’s house at 7:00 pm the next evening. Bartlett was having “a large party” and told the owner to leave. The owner recounted,

I took off my coat and waistcoat, and walked into the hall where the company was assembled . . . the lady of the house cried out, “my dear Dr. Bartlett, that man is crazy or drunk!” “No, I beg your pardon, Madam! . . . but your husband wishes to rob me of my property, so I will give him all my clothes too.” The Doctor rose up in a great passion, drew out his sword, and . . . said he would split my head.^459^

When Bartlett was restrained by one of the gentlemen present, Bartlett threw down his sword, asked his servant for paper and ink, wrote permission for the owner to retrieve only his personal effects, and then invited the owner to sit and have a glass of wine. The owner refused and recalled that the next day, the townspeople “all pointed at me and said, that is the gentleman who refused yesterday to drink a glass of wine with Doctor Bartlett.”^459^

In June 1800, Bartlett returned to Newport, explaining that his health had suffered from his time practicing in the West Indies, and that friends of his father should see him for care.^460^ In July, the town suffered an outbreak of yellow fever after the arrival of the United States frigate *General Greene*. Bartlett wrote that the town council asked him to examine the sailors, and afflicted townspeople^454^; however, the ship surgeon, not wanting to appear as if the town doubted his authority, responded that the town council only asked Bartlett to attend to 3 particular patients.^461^ The town council and the health officer confirmed the ship surgeon’s account. Bartlett wrote that because the fever was infectious, it was necessary to move the ship away and sink it in sea water for 2 weeks, but the town council did not heed his advice.^454^ Yellow fever produced loss of eyesight and pain above and in the eyes, as they become “inflamed, watery, protruded.”^454^

Unfortunately, Bartlett’s move from the Caribbean did not help his health. Bartlett became severely ill with the “pestilential fever” and experienced bleeding from the mouth and nose, headache, loss of memory, vertigo, and “a disposition to coma.”^454^

In Newport, he first “performed many operations in the Eyes—such as removing the Cataract by depression and extraction . . . also Films, Gutta Cerena, Ophthalmia’s, &c.”^93^ His fee schedule of August 1801 listed the operations “for extracting or couching the cataract 50 dollars” and noted these had not been previously performed in the area.^190^ In December of 1801, Bartlett submitted for copyright “A Treatise on Rules of Health” which prevention and cure of diseases for all ages. The cures included “the effect of electric influence.”^462^

Bartlett was present in the New York yellow fever epidemic of 1801, which took the life of Bayley, and judged it to be similar to his previous experiences.^93^ Bartlett returned to Newport at the end of 1801 and took umbrage at the claims of a fellow surgeon named Horace Senter. Bartlett was

astonished at the presumption of a Mr. Senter, in . . . saying “that Operations in the Eye by extracting the Cataract; and cutting for the Stone in the Bladder, had not been performed by any Person residing in this State.”^93^

In February 1802, he was admitted to the First Congregational Church of Stonington,^463^ but later that month he announced that his time visiting family in Connecticut was over, and he was returning to practice in Newport.^464^

He began practicing in Hartford, and in May 1803, responded in print to his critics, by explaining that although 4 patients had died after seeing him, they were under the care of other doctors at the time, and this was out of 1067 total patients that he had seen around Hartford.^428^ He indicated that he had received a fee schedule from surgeon Mason Fitch Cogswell (1761-1830) of that city. Bartlett noted, “Lithotomy . . . and extracting the cataract from the eyes, Dr. Bartlett is informed are not practiced in this neighborhood.”^428^

In August 1803, Bartlett left his family in Hartford and traveled to New York to assist with a yellow fever outbreak.^281^ He was doing poorly financially and left shop furniture for sale in Hartford. He hoped to earn enough in New York to satisfy his debts. In New York, he noted that he “has recently recovered from the effects of the Pestilential fever.”^281^ Even though he was declared an insolvent debtor in early 1804,^465^ he remained in New York and highlighted his talents as “. . . an experienced operator in the eyes, by removing blindness occasioned by cataracts, films, &c.”^94^ He requested patients come to his house for treatment, as his “present ill state of health” made his travel difficult.^94^ Also that Spring, he advertised that he “will take six Pupils, . . . operations . . . couching and extracting cataracts.”^466^ He also published the idea that steam from a bellows forced into the lungs could reanimate the dead after drowning, strangulation, or hanging.^467^ He had intended to publish the idea in a book but believed that medical necessity required immediate publication in the newspaper.^467^

Cataract surgery, lithotomy, and treatments “to cure cancers” continued to be his specialties when he moved to New Bedford, Massachusetts, in later in 1804.^468^ He also announced that he was about to publish a 700-page medical book.^468^

Bartlett probably never fully recovered from the yellow fever, and his finances were poor. He had burned his bridges in the Northeast, in Charleston, and in the West Indies. He traveled to what was then the Southern-most port in the United States—Darien, Georgia. When he finally succumbed to his illness at the age of 40 years in July 1806, the obituary stated “He has left a truly distressed family—a widow and three orphan children.”^469^

### Horace Senter, 1801

Horace Gates Senter (1780-1804) was the son of Isaac (1753-1799) and Elizabeth Senter of Newport.^470^ The elder Senter was a surgeon most remembered for serving during the failed assault of the Continental army on Quebec in 1775. When Benedict Arnold’s leg was pierced with a musket ball, Senter recorded “I easily discovered and extracted it.”^470^ The family had moved to Newport by 1787.^471^

Horace Senter was “possessed of a mind uncommonly active and promising, from his genius, to follow in the bright train which the example of his venerated father marked out for him.”^472^ At his graduation from Rhode Island college in 1796, he debated the merits of establishing a uniform national educational system.^473^ He received a “Master of Arts” there in 1799.^474^

Senter was close with John Collins Warren when the 2 trained at Guy’s Hospital in London. Warren wrote “No one comes to see me but Senter.”^475^ Colleagues remembered Senter as “a most zealous anatomist.”^476^ Senter helped Warren dissect a hypertrophied heart, which was preserved in the museum of the Medical College.^475^ John Collins Warren remembered that the preparation of anatomic specimens was “a primary occupation and a pleasure.”^475^ He enjoyed “nicely injecting a delicate piece of anatomy . . . and of enclosing it in an elegant glass vessel of perfectly transparent liquid.”^475^ He performed “blood-vessel injections” of the specimens.^475^ Typically, pigments suspended in resin or glue were injected.^203^ But Warren remembered “Senter was ahead of me in this art, and had made a collection, which, though small, contained many beautiful pieces.”^475^ Senter performed “minute injection of the vessels of the eye” when preparing a collection of 10 specimens of the choroid, retina, ciliary processes, vortex veins, and iris.^476^

Senter returned to Newport in November 1800^477^ and soon announced his intention to succeed his father in the practice of medicine.^478^ In November 1801, he wrote that his services were available “As the Operations for the Stone in the Bladder, and the Extraction of the Cataract from the Eye, have never been performed by Surgeons residing in this state . . .”^479^ As Charles F Bartlett had already advertised these types of surgeries in August of that year,^190^ Bartlett registered a protest with the paper. Of course, Leprilete had advertised cataract extraction in Rhode Island 20 years before both of them.

In the summer of 1802, Senter’s recently widowed mother died unexpectedly.^480^ Also that summer, John Rutledge of South Carolina vacationed in Rhode Island. Senter was alleged to have had an affair with Rutledge’s wife, however, it was not immediately discovered.^481^

Around New Year’s Day of 1804, Rutledge learned that Senter was in Charleston.^481^ By that point, Rutledge knew about the alleged affair, having discovered “amorous letters.”^482^ Senter was conversing with Mrs Rutledge in her home when her husband burst in and fired at Senter, shooting off one of his fingers.^482^ Senter ran away, and hid in the woods all night while Rutledge’s “negroes” pursued him.^482^ Senter made his way to Charleston, where Rutledge challenged him to a duel, which took place in Savannah a few days later.^481,482^ Senter fired first, merely grazing Rutledge.^481^ Rutledge’s shot passed through the bone just below the knee, and the duel was terminated.^481^ Senter’s leg was amputated, but he died 2 days later, on January 19, 1804.^483,484^

**Figure 30.**
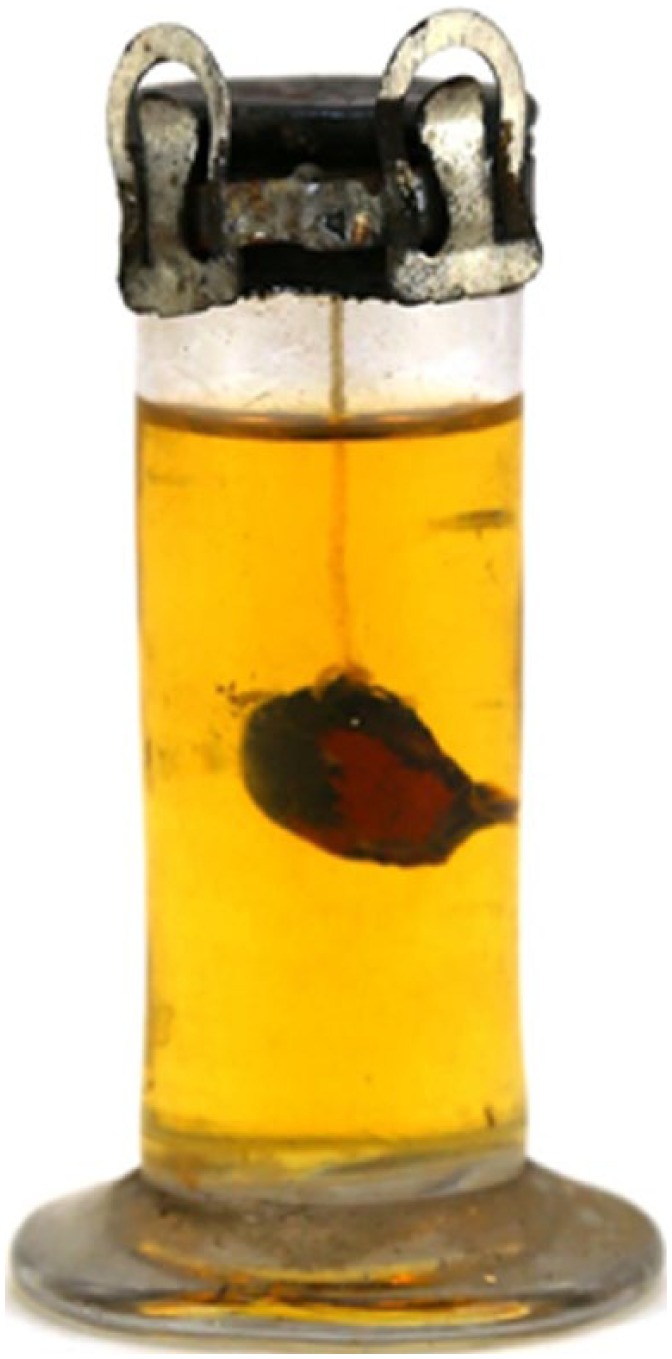
Eye specimen prepared by Horace Senter, after injection of the “Venae vorticosae,” specimen 513 of the Warren Anatomical Museum.^476^

With both his parents having recently preceded him in death, his hometown paper lamented “Dr. Senter was the only remaining hope of his once esteemed family.”^472^ A fellow student of Senter’s wrote “Poor Senter! I am not surprised at his end. I think he was a little too impetuous in his manner.”^475^ Warren acquired the anatomic specimens prepared by Senter^475,476^ and donated them to the permanent collection of Harvard University (Figure 30). Although Warren would go on to become one of the greats of American medicine, Senter’s potential was never realized due to his personal shortcomings.

## Overview of European Ophthalmology in North America

One limitation of our review is that some eye surgeries might have been performed at a very low level in the early Colonial period without leaving specifics in the written record about the type of surgery performed. For instance, a Trenton newspaper reported in 1735 that “a certain person who lives near the Yardley ferry has lately turned oculist,” and that “an experiment upon Mr. Benjamin Randolph has caused him to become quite blind and in great pain.”^485^

Although the native American healers we uncovered were predominantly women, the European healers were almost all men. In fact, we did not discover any female surgeons in this group.

Although the medical communities in Europe and in Latin America were tightly controlled (by the royally appointed protomedico in the latter),^4^ the oculists in the English colonies appeared to be relatively unregulated. Any man who did an apprenticeship or attended lectures could call himself “Doctor.” (The few women practitioners we uncovered did not use this title.)

It would be easy to imagine that the early oculists were divided into unethical itinerant quacks who did not further American medical development and ethical surgeons who planted roots in their local medical community and had university training or affiliation. However, such a simple distinction misses many nuances. Itinerants have occasionally been accused of moving along before the bandages came off postoperatively. In fact, the itinerants typically stayed at least a few months, if not years, in a given city. Often their movements can be explained by war, local competition, or the need to find unoperated patients. Some of the itinerants had more university training than community surgeons who completed an apprenticeship.

As for medical and surgical skill, the university-trained physicians applied treatments such as copious bleeding and mercury. The superiority of this approach over placebo medicines is not obvious. We might imagine that skill would be related to surgical volume. Sometimes the mainstream physicians merely observed a few cases in their training and then performed a handful throughout their careers. In fact, they may have learned by watching an itinerant. An itinerant who focused on eye surgery over a long period, such as Stork or Jericho, probably would have better results than a mainstream credentialed nonspecialist who only did a few cases.

Some of the itinerants offered therapies such as electricity, magnetism, or reanimation of the dead. For Graham and Yeldall, the electrical and/or magnetic treatments actually came after their American tours. Moreover, even mainstream scientists and physicians were experimenting with these treatments.

Certainly many of the itinerants committed crimes and other misdeeds of varying severity. Mercier was convicted of murder. Charles Bartlett was a privateer and was accused of piracy. Graham lied about performing a cataract extraction. But these lapses (as serious as some of them were) do not automatically render all of their medical care meaningless. Graham probably had a good understanding of eye physiology and pathology for the time and lectured on topics such as glaucoma not generally discussed in the colonies. Bartlett might have exposed community surgeons in the Northeast to the technique of cataract extraction. Mercier’s descriptions of the results of his couching were more realistic and circumspect than those of the well-respected Jackson.

Moreover, the mainstream surgeons were not without their own lapses. They were accused of improper acquisition of anatomic specimens (Bayley), disloyalty to country (Church), and misappropriation of public resources (Shippen).

In England, surgeons, with the title “Mr.”, and physicians, with the title “Dr.”, were (and still are) separate.^486^ The lack of regulation in the British colonies meant that this distinction did not apply. One practiced any aspect of medicine or surgery with which one felt comfortable. Therefore, patients were not adequately protected. Anyone could advertise, perform medical and surgical treatments, and acquire a good (or bad) reputation.

The Revolution delayed the development of ophthalmology in America (Figure 6). Loyalist surgeons were expelled. Those who favored independence became preoccupied with both wartime medical care and political activities. Practitioners from abroad refrained from migrating to the war-torn colonies.

In the half-century following Daviel’s introduction of planned cataract extraction, couching was still the predominant technique in America (Figure 6). Even many who had exposure to extraction, such as Tyler, Warren, and Smith, preferred couching. Given the lack of anesthesia, an understanding of antisepsis, preoperative pupillary dilation, or corneoscleral suturing, it is easy to see how cataract extraction would be difficult to accomplish successfully.

Certain advances in the American colonies rivaled the innovations in Europe. In 1801, Samuel Brown, a doctor in the frontier territory of Kentucky, observed poisoning by *Datura stramonium* and suggested that this agent be applied topically to dilate the pupil before cataract extraction. After the return of his son from European training, John Warren at Harvard recommended treating angle closure glaucoma by lens extraction. Other eye procedures described or advertised in America before the 19th century included enucleation, resection of conjunctival lesions or periocular tumors, treatment of lacrimal fistula, and fitting of prosthetic eyes.
